# Phylogeny of Echinoderm Hemoglobins

**DOI:** 10.1371/journal.pone.0129668

**Published:** 2015-08-06

**Authors:** Ana B. Christensen, Joseph L. Herman, Maurice R. Elphick, Kord M. Kober, Daniel Janies, Gregorio Linchangco, Dean C. Semmens, Xavier Bailly, Serge N. Vinogradov, David Hoogewijs

**Affiliations:** 1 Biology Department, Lamar University, Beaumont, Texas, United States of America; 2 Department of Statistics, University of Oxford, Oxford, OX1 3TG, United Kingdom; 3 Division of Mathematical Biology, National Institute of Medical Research, London, NW7 1AA, United Kingdom; 4 School of Biological & Chemical Sciences, Queen Mary University of London, London E1 4NS, United Kingdom; 5 Department of Ecology & Evolutionary Biology, University of California Santa Cruz, Santa Cruz, California, United States of America; 6 College of Computing and Informatics, University of North Carolina at Charlotte, Charlotte, North Carolina 28223, United States of America; 7 Marine Plants and Biomolecules, Station Biologique de Roscoff, 2968 Roscoff, France; 8 Department of Biochemistry and Molecular Biology, Wayne State University School of Medicine, Detroit, Michigan 48201, United States of America; 9 Institute of Physiology, University of Duisburg-Essen, Essen, Germany; Laboratoire Arago, FRANCE

## Abstract

**Background:**

Recent genomic information has revealed that neuroglobin and cytoglobin are the two principal lineages of vertebrate hemoglobins, with the latter encompassing the familiar myoglobin and α-globin/β-globin tetramer hemoglobin, and several minor groups. In contrast, very little is known about hemoglobins in echinoderms, a phylum of exclusively marine organisms closely related to vertebrates, beyond the presence of coelomic hemoglobins in sea cucumbers and brittle stars. We identified about 50 hemoglobins in sea urchin, starfish and sea cucumber genomes and transcriptomes, and used Bayesian inference to carry out a molecular phylogenetic analysis of their relationship to vertebrate sequences, specifically, to assess the hypothesis that the neuroglobin and cytoglobin lineages are also present in echinoderms.

**Results:**

The genome of the sea urchin *Strongylocentrotus purpuratus* encodes several hemoglobins, including a unique chimeric 14-domain globin, 2 androglobin isoforms and a unique single androglobin domain protein. Other strongylocentrotid genomes appear to have similar repertoires of globin genes. We carried out molecular phylogenetic analyses of 52 hemoglobins identified in sea urchin, brittle star and sea cucumber genomes and transcriptomes, using different multiple sequence alignment methods coupled with Bayesian and maximum likelihood approaches. The results demonstrate that there are two major globin lineages in echinoderms, which are related to the vertebrate neuroglobin and cytoglobin lineages. Furthermore, the brittle star and sea cucumber coelomic hemoglobins appear to have evolved independently from the cytoglobin lineage, similar to the evolution of erythroid oxygen binding globins in cyclostomes and vertebrates.

**Conclusion:**

The presence of echinoderm globins related to the vertebrate neuroglobin and cytoglobin lineages suggests that the split between neuroglobins and cytoglobins occurred in the deuterostome ancestor shared by echinoderms and vertebrates.

## Introduction

Although genomic information accrued over the last decade has demonstrated the existence of globins in all three kingdoms of life [1], far beyond the familiar myoglobin (Mb) and α- and β-globins, it has also unexpectedly expanded the repertory of vertebrate globins. Over a decade ago, Burmester, Hankeln *et al*. identified neuroglobin (Ngb), expressed primarily in neurons of the central and peripheral nervous systems and eye tissue [2]. Concomitantly, three groups discovered another major globin, cytoglobin (Cygb), expressed mostly in fibroblasts and internal organs [3–5]. Additional globins with more limited distributions were also found, including globin E (GbE), an eye-specific globin present only in birds [6], globin X (GbX) found in amphibians and teleost fish [7], and globin Y (GbY) found so far only in the genome of the frog *Xenopus tropicalis* [8]. GbX was shown more recently to be present in lampreys, several protostomes, a shark and in reptiles [9], and to also represent a family of membrane-bound globins [10]. Despite more than a decade of intensive research, the functions of Ngb and Cygb remain unclear [11, 12]. Molecular phylogenetic analyses of vertebrate globins agree that GbX is closely related to Ngbs, while Cygbs are related to cyclostome hemoglobins (Hbs), Mb, GbE, GbY and the Hb α- and β-globins [13–17]. The latest addition, is the large chimeric androglobin (Adgb), that appears to be phylogenetically related to Ngb [18]. We report below the results of a molecular phylogenetic analysis of 52 echinoderm Hbs and representative vertebrate Hbs designed to test the hypothesis that Ngb and Cygb-like globins are also present in echinoderms.

Together with chordates and hemichordates, echinoderms, animals with “spiny skin”, form the deuterostome superphylum, one of the two major divisions of animals [19, 20]. All echinoderms share the following characteristics: (a) a pentameral radial symmetry in adults, imposed on a bilateral larval symmetry, (b) a unique water vascular or “ambulacral” system, comprising a network of fluid-filled canals, that serves functions in feeding, gas exchange, and also in locomotion, and (c), a stereom, a mesh-like endoskeleton comprised of calcite (calcium carbonate) plates and pores covered by a thin epidermis [20]. Echinoderms are classified into the free-moving Eleutherozoa, which include the Asteroidea (starfish, ~1750 species), the Echinoidea (sea urchins and sand dollars, about 900 species), the Holothuroidea (sea cucumbers, ~1430 species), the Ophiuroidea (brittle stars, ~2,300 species), and the sessile Pelmatozoa, encompassing the Crinoidea (feather stars and sea lilies, about 1430 species) [21]. Although the approximately 7,000 species of extant echinoderms occur in marine habitats extending from shallow intertidal zones to the deep ocean floor, the majority live in the benthic zone, starting from the shoreline, and extending downward along the continental shelf to the latter’s edge, at a depth of about 200m [21]. Echinoderms also subsume nearly 13,000 extinct species, some dating back to the Cambrian period, that lasted from about 540 to 490 Mya (million years ago), and coincided with a rapid expansion in multicellular life forms, the Cambrian explosion [22].

Although Hbs were recognized to occur in echinoderms early on [23, 24], very little is known about them. Intracellular Hbs occurring in coelomocytes present in the water vascular system (WVS), have been reported in two of the five classes of echinoderms: the holothurians (sea cucumbers) and ophiuroids (brittle stars) [25, 26]. The biochemical properties of the Hbs from the ophiuroids *Hemipholis cordifera (elongata)* and *Ophiactis simplex* have been investigated [27–29], as well as of the coelomic Hbs from several sea cucumbers, including *Caudina arenicola*, *Thyonella gemmata* and *Paracaudina chilensis* [27, 30–39]. Furthermore, the amino acid sequences of the latter and the crystal structure of two *C*. *arenicola* coelomic Hbs have been determined [40–43]. The gene structures for the Hbs of *C*. *arenicola* and the brittle *star O*. *simplex* have also been reported [44, 45].

The first annotation of the genome sequence of the echinoid sea urchin *Strongylocentrotus purpuratus* [46] revealed the presence of a very unusual chimeric, multi-domain globin [47, 48] as well as a single domain globin, that was shown to be characteristic of a new globin family, the chimeric androglobins, which are present in all chordates [18]. The advent of a revised annotation of the sea urchin genome sequence using next generation transcriptome sequence data [48] has necessitated a further analysis of its Hbs.

## Results

### Sea urchin Hbs

The first annotation of the *S*. *purpuratus* genome sequence [46] revealed the presence of a highly unusual, large, chimeric multidomain Hb and of a single domain globin, that was shown subsequently, to define the androglobin family [18], consisting of large (>1500 residues) chimeric proteins with a cysteine proteinase N-terminal and a central, circularly permuted globin domain. Subsequent analysis showed that, in addition to the androglobins, the sea urchin possessed a 16-domain Ngb-like globin [47]. The advent of a more recent annotation of the sea urchin genome, Spur_v3.1 (2011/06/10), informed by next generation transcriptome sequence data [48], has necessitated a revised understanding of its globin genes (see Table 1). The 16-domain protein identified earlier as a Ngb [47] is now understood to comprise a 14-domain protein (XP_001199205.2), and a 416 residue protein (XP_003725467.1), the latter consisting of the two N-terminal globin domains. Furthermore, a new 166 residue globin (XP_003729167.1) was identified, and observed to be homologous to Cygb. The two androglobin isoforms are still present in the new annotation, as is the unique 267 residue single Adgb (XP_001195639.2) (see Table 1).

**Table 1:** Globins identified in the latest annotation of the *S*. *purpuratus* genome sequence, Spur_v3.1 (2011/06/10).

Identifier	No. of residues	Remarks
XP_001199205.2	2146	14 covalently linked globin domains
XP_003725467.1	416	2 covalently linked globin domains and an unknown N-terminal
XP_003729167.1	166	New, single domain globin
XP_001195639.2	267	Single androglobin domain
XP_001186225.2	1765	Androglobin isoform 1
XP_003724134.1	1841	Androglobin isoform 2

A MAFFT L-INS-i alignment of the 14 domains of the 2146-residue multidomain protein, the 2 globin domains of the 416 residue protein and sperm whale Mb is provided in S1 Fig, together with the canonical Mb-fold [49]. It is evident that all the globin domains adhere to the Mb-fold and contain the required distal His at position F8. Only the second globin domain of the 416 residue protein, missing helices G and H, can be regarded as defective.

### Other echinoderm Hbs

We sought to identify homologs of the *S*. *purpuratus* globins in the genomes and transcriptomes of other echinoderms. S1 Table lists the echinoderm species whose genomes/transcriptomes yielded hits in BLASTP and TBLASTN searches using the *S*. *purpuratus* globins as queries, including additional echinoid genomes [50–54]. Overall, some 52 echinoderm globin sequences were identified in recently available genomes and transcriptomes (http://echinodb.uncc.edu/), including sequences found in the literature [40, 41, 43, 55]. A MAFFT L-INS-i alignment of the 52 sequences was subjected to the ExPASy Decrease Redundancy Tool (web.expasy.org/decrease_redundancy/‎), set at 90% identity. The resulting similarity matrix and the remaining 38 sequences are provided in S2 Fig. S2 Table lists the observed locations of introns in selected echinoderm Hbs. The presence of conserved intron positions B12.2 (between positions 2 and 3 of codon 12 in helix B) and G7.0 (between the codons for amino acids 6 and 7 in globin helix G), found in most metazoan globins, provides additional support for their globin identity.

### Molecular phylogeny of echinoderm Hbs

**Fig 1:**
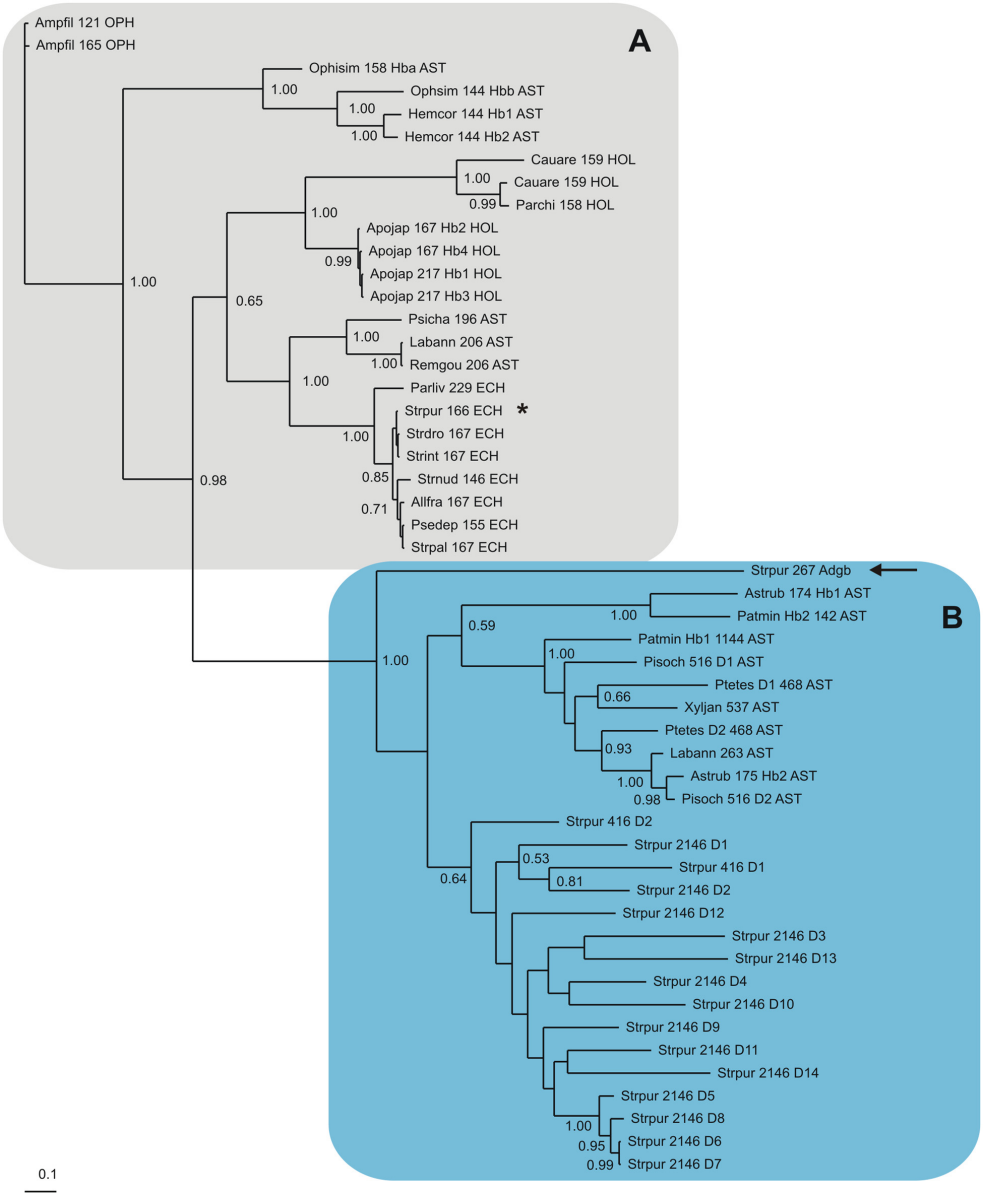
Bayesian phylogenetic tree based on a MAFFT L-INS-i MSA of 52 Echinoderm Hbs.

An unrooted Bayesian tree of a MAFFT L-INS-i MSA (GUIDANCE score 0.955) of the 52 echinoderm globins is shown in Fig 1. It demonstrates that the sequences cluster in two approximately equally-sized groups. Group A (labeled in grey) comprises the *S*. *purpuratus* 166 residue Hb (XP_003729167.1), and its ophiuroid, holothuroid, asteroid and other strongylocentrotid orthologs. Group B (shaded light blue) is supported with highest Bayesian posterior probability, and includes the domains of the *S*. *purpuratus* 2146 residue multidomain (XP_001199205.2) and the 416 residue 2-domain Hb (XP_003725467.1), several homologous asteroid Hbs and the single domain Adgb (XP_001195639.2).

### Relationships between vertebrate and echinoderm Hbs

**Fig 2:**
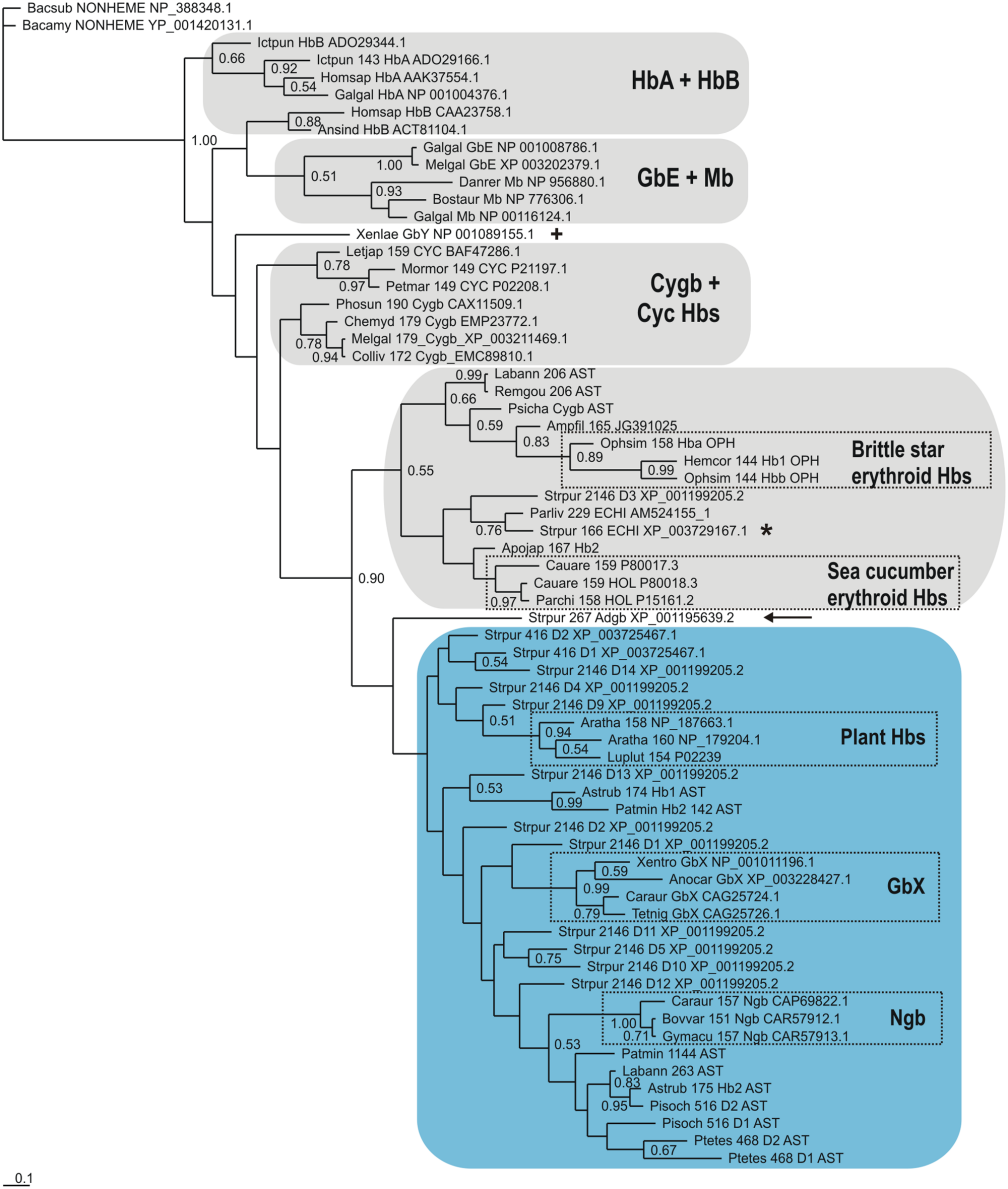
Bayesian phylogenetic tree based on a MAFFT L-INS-i MSA of 36 Echinoderm Hbs and 30 Vertebrate Hbs, using the *Bacillus* nonheme globin sequence [56], as outgroup.

**Fig 3:**
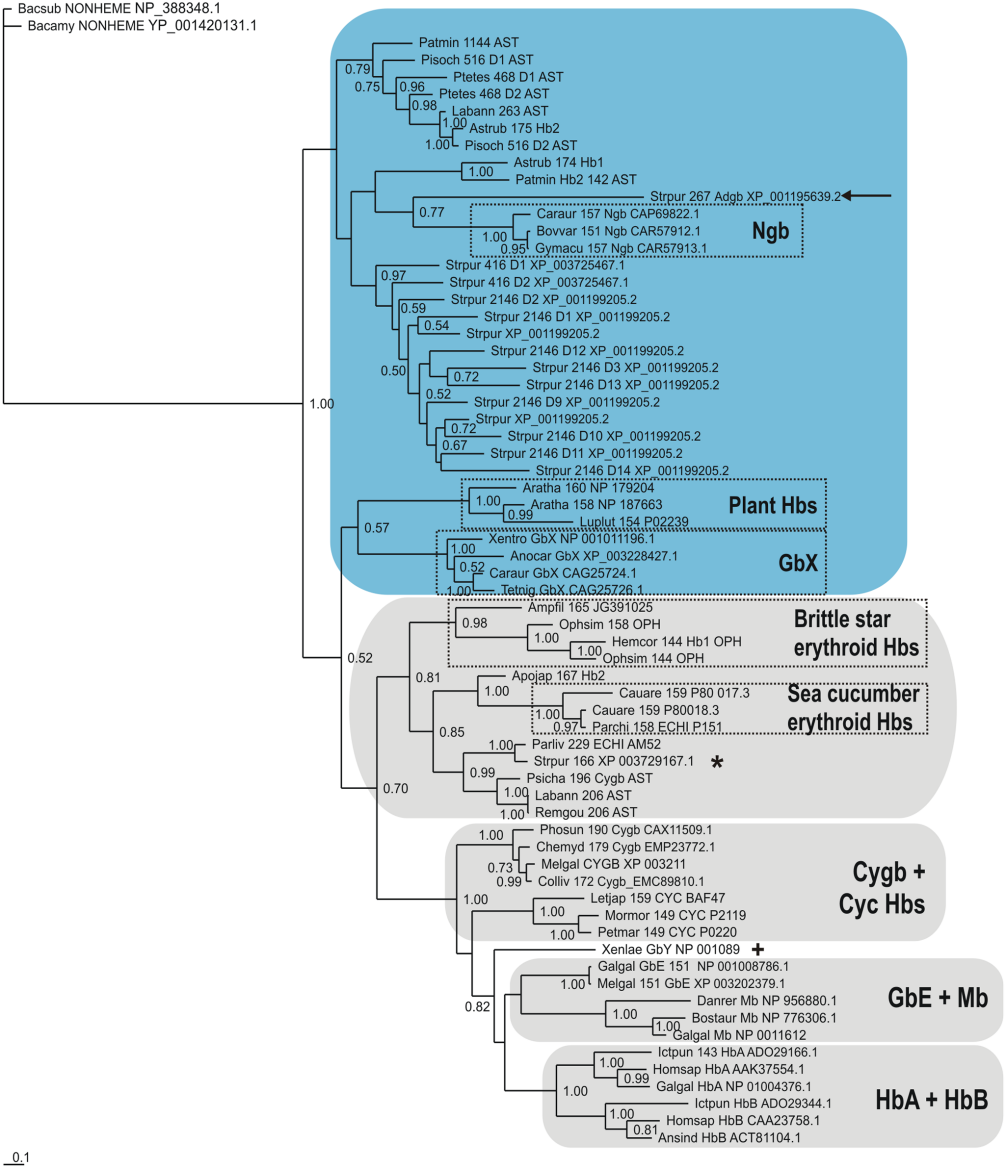
Bayesian phylogenetic tree based on a T-Coffee Expresso MSA of 36 Echinoderm Hbs and 30 Vertebrate Hbs, using the *Bacillus* nonheme globin sequence [56], as outgroup.

**Fig 4:**
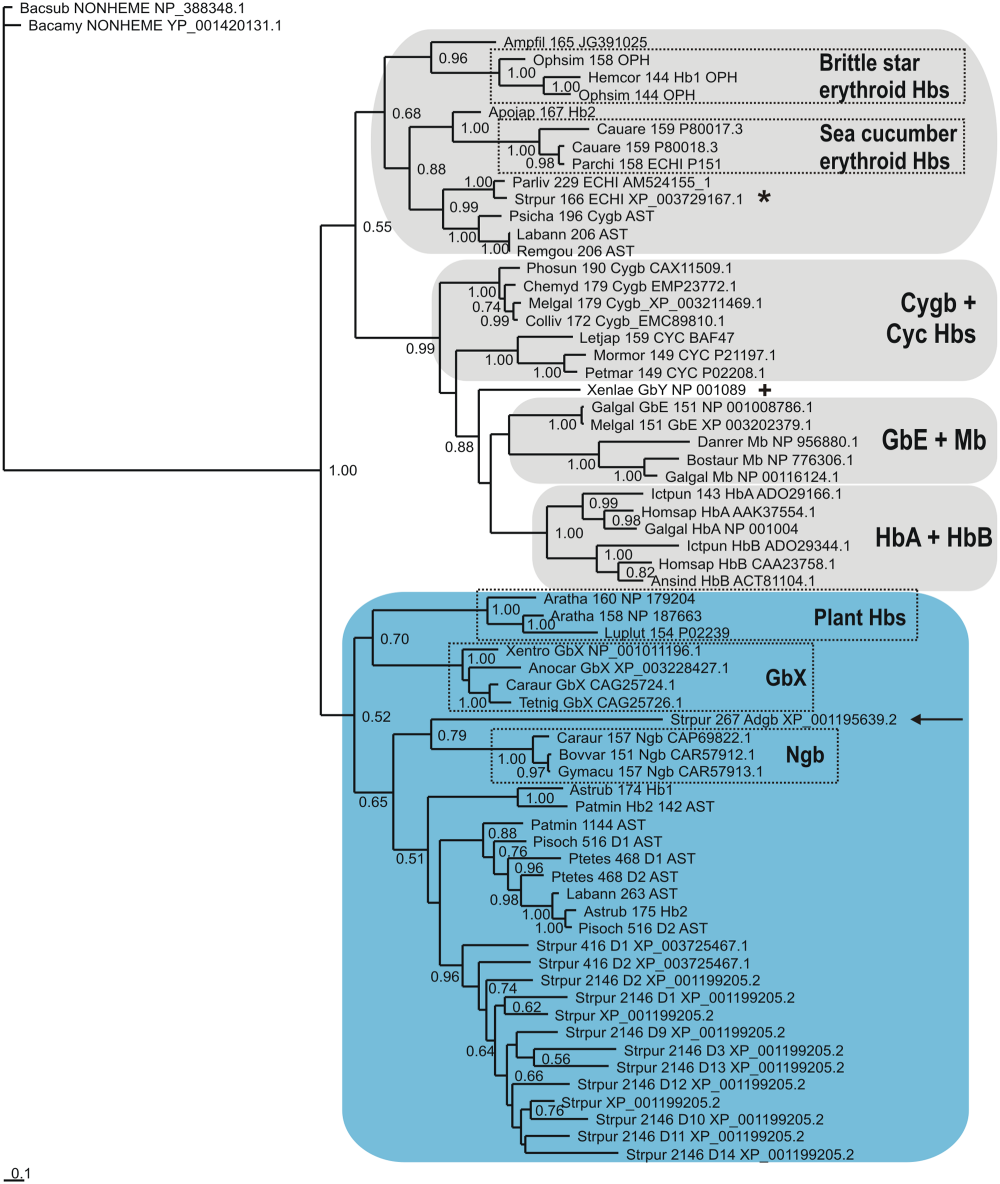
Bayesian phylogenetic tree based on a Clustal Omega MSA of 36 Echinoderm Hbs and 30 Vertebrate Hbs, using the *Bacillus* nonheme globin sequence [56], as outgroup.

A Bayesian tree based on a MAFFT L-INS-i MSA of 36 echinoderm globins (a reduced set selected upon exclusion of redundant sequences) with 21 vertebrate Hbs (representing the 8 subfamilies) is shown in Fig 2. The sequences are identified by the first 3 letters of the genus and species parts of the binomial, the number of residues, globin subfamily if known, a 3 letter abbreviation of the class and the GenBank identification. We used the nonheme globin from *Bacillus subtilis* [56] as an outgroup, a successful strategy employed earlier [57, 58]; although this globin exhibits a 3/3 Mb fold, its heme binding cavity is defective due to excessive separation of helical strands. The Cygb branch including GbE, HbA, HbB and the two cyclostome Hbs is clearly separated from the Ngb branch, the latter also including GbX. The Adgb (marked by an arrow) occurs between the GbX’s and the Ngbs, consistent with previous observations [18]. The echinoderm Hbs comprising Group B in Fig 1 cluster next to the Ngbs (shaded light blue), while the echinoderm Hbs from Group A in Fig 1, including the 166 residue globin, XP_003729167.1 (marked by a star), cluster with the Cygbs (shaded grey). This pattern is also observed in trees obtained using alignments generated by T-Coffee Expresso and Clustal Omega, shown in Figs 3 and 4, and the results of analyses obtained using Neighbor Joining and Maximum Likelihood methods were in broad agreement with those of the Bayesian inference method. All our trees reproduce the previously-reported phylogeny of the vertebrate globins, illustrated in S3 Fig. However, due to the high levels of sequence divergence, and relatively short sequence length, sequence-based phylogenies of globins typically feature high statistical uncertainty, irrespective of the alignment method employed, the type of phylogenetic analysis, and the evolutionary models used.

### Structural alignments

**Fig 5:**
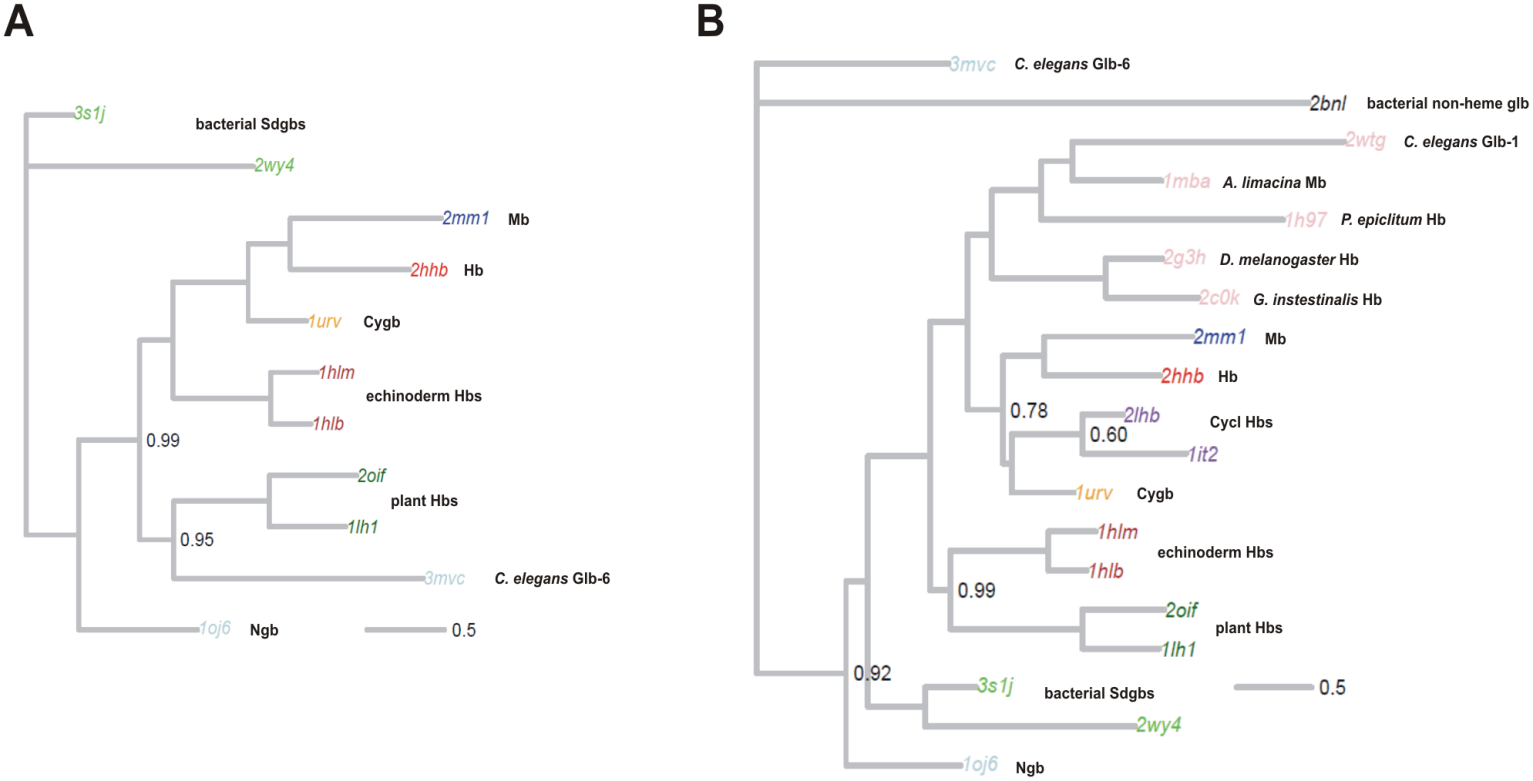
(A) Consensus tree generated by StructAlign, which carries out joint Bayesian inference of alignments and trees under a joint model of sequence and structure evolution. The structures used here correspond to echinoderm coelomic Hbs (1hlb,1hlm), vertebrate Ngb (1oj6), Cygb (1urv), Mb (2mm1), HbA (2hhb) *C*. *elegans* Ngb (3mvc), plant Hbs (2oif, 1lh1), and two bacterial SDgbs (3s1j,2wy4). (B) Tree generated using a larger dataset, consisting of the aforementioned structures augmented with cyclostome Hbs (2lhb, 1it2) *A*. *limacina* Mb (1mba), *D*. *melanogaster* Hb (2g3h), *G*. *intestinalis* Hb (1c0k), *C*. *elegans* Glb-1 (2wtg), *C*. *lacteus* Ngb (2xki), and an bacterial non-heme globin (1bnl) as an outgroup.

In order to better resolve the uncertain regions of the tree we also generated trees using the StructAlign package [59, 60] which performs joint sampling of alignments and phylogenies and structural superpositions, under a joint evolutionary model for sequences and structures. Since the globin structures are highly conserved, this method allows for more accurate and reliable alignments to be generated, and reduces the uncertainty associated with the resulting trees. Due to the additional computational complexity associated with this approach, StructAlign inference was carried out on smaller datasets containing a selection of representative structures. The two *C*. *arenicola* coelomic Hb structures (PDB: 1hlb, 1hlm) were initially combined with a set of 9 additional Hb structures, representing vertebrate Ngb (1oj6), Cygb (1urv), Mb (2mm1), HbB (2hhb), plant Hbs (1lhl, 2oif), bacterial SDgbs (2wy4, 3s1j), and an invertebrate nerve globin (3mvc). In order to assess sensitivity to the choice of dataset, analysis was also re-run after adding in a set of protostome Hbs (1mba, 2wtg, 3g3h, 2c0k, 1h97), as well as two cyclostomes (2lhb, 1it2), and a bacterial nonheme globin (2bnl) as an outgroup. The consensus trees are shown in Fig 5. In both cases the *C*. *arenicola* globins are placed between the plant and Cygb clades, recapitulating the pattern seen in the sequence-based trees.

**Fig 6:**
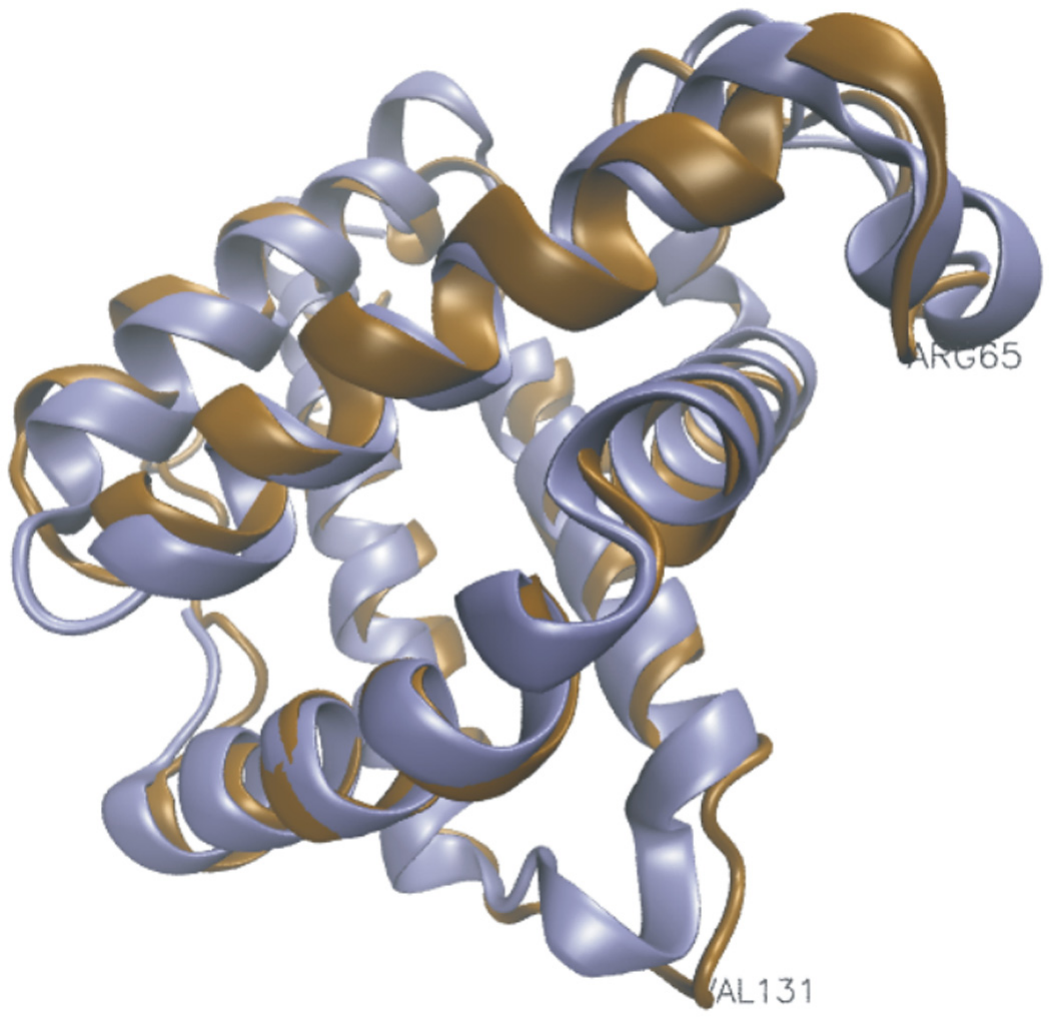
Structural superposition of the echinoderm structure 1hlm (brown) with the cytoglobin structure 1urv (blue). The two structures show a very close correspondence, with some localized deviations, such as at helix D, which is disordered in 1hlm (marked by Arg65), and at the C-terminal end of helix G (marked by Val131).

Fig 6 displays a maximum likelihood structural superposition of the echinoderm structure 1hlm (brown) with the Cygb structure 1urv (blue), as generated by StructAlign. In most places there is a very close correspondence between the structures, with some noticeable deviations at helix D, and the C-terminal end of helix G (as labelled). A maximum likelihood structural superposition of the 1urv structure (red) with the extension containing Cygb structure 2dc3 (cyan) essentially results in similar results (S4 Fig).

**Fig 7:**
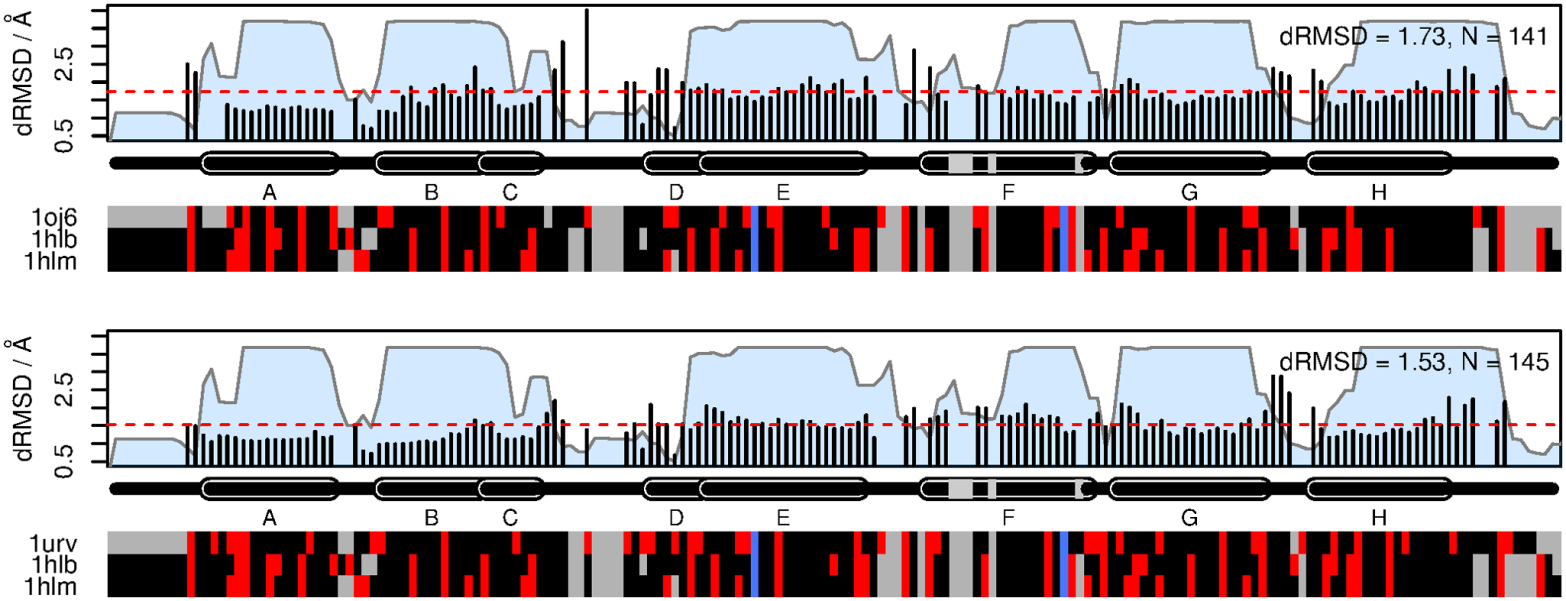
Analysis of average per-site root mean square deviation (RMSD) between the echinoderm structures (1hlb, 1hlm) and the Ngb (1oj6) and Cygb (1urv) structures. Structural deviation was computed from each of the echinoderms to the target structure of interest as described in the Methods section, using the consensus alignment generated on the larger structural dataset; the mean of these two values computed for each column. Colored blocks at the bottom indicate charged (red) and non-charged (black) residues. Gaps are shown in grey, and the proximal and distal histidines are shown in blue. Helix locations are annotated above, and named using standard conventions. Overall RMSD for each plot is computed as the mean of the squared contributions from each site, and is indicated by the dashed red line. The blue areas underneath indicate the confidence associated with each column in the multiple alignment, as outputted by StructAlign.

In order to further investigate the structural features influencing the placement of these two echinoderms closer to Cygb, we conducted an analysis of the per-site root-mean square deviation (RMSD) for pairwise comparisons between the echinoderm structures and Ngb (1oj6) and Cygb (1urv) structures. As shown in Fig 7, the average pairwise RMSD is lower when comparing the echinoderms with 1urv (1.53Å vs. 1.73Å with 1oj6), and the length of the aligned region is also longer (145 residues, versus 141 for 1oj6). There are also some localized regions of higher structural deviation when comparing to 1oj6, for example in between helices C and D, where there is a 2-residue insertion (N45, G46) in 1oj6. The latter difference could have functional implications for the flexibility of the CD corner and formation of the hexacoordinate state. It can also be seen that there are some regions of higher structural deviation with respect to 1urv, especially at the ends of helix G.

**Fig 8:**
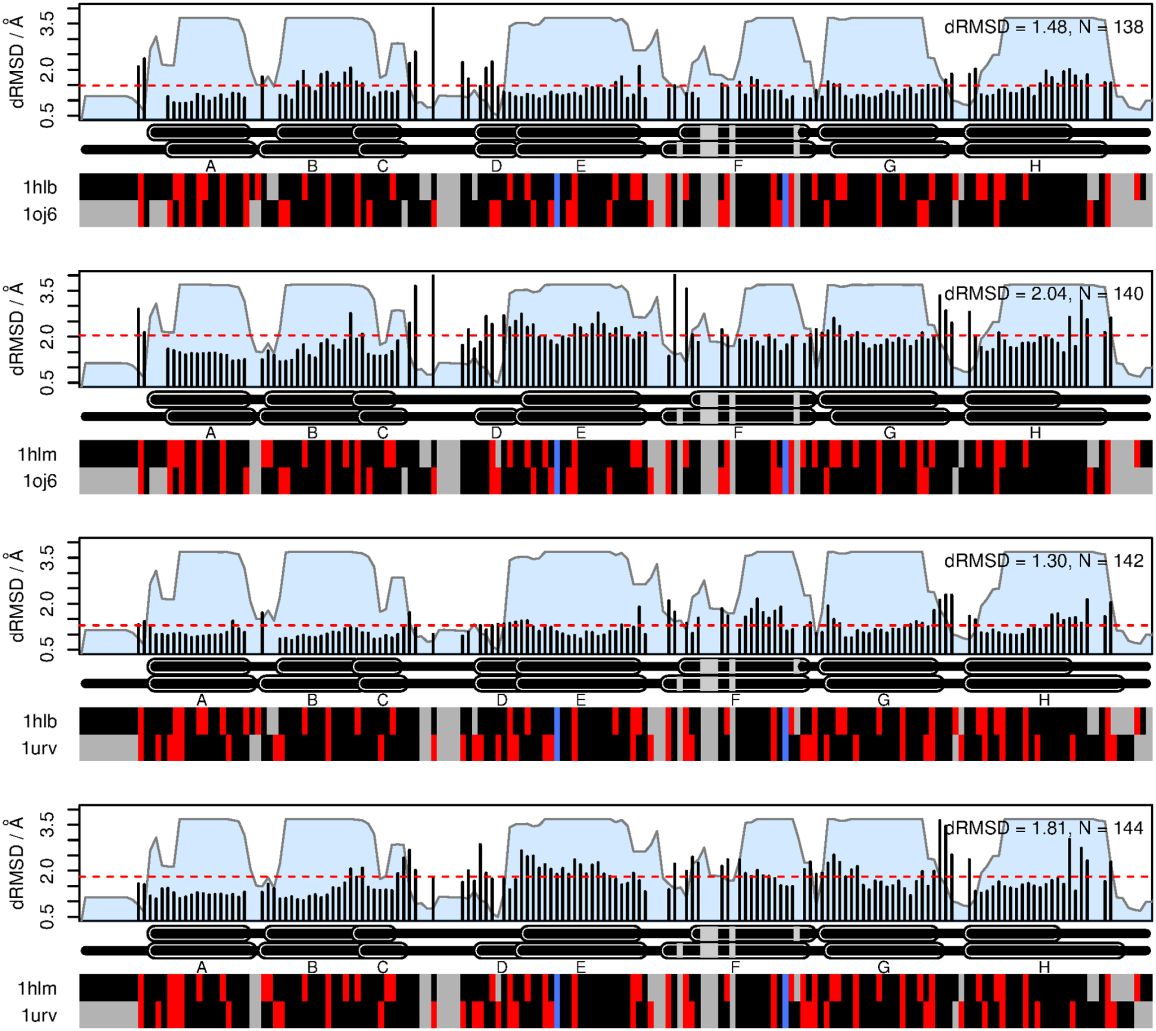
Analysis of pairwise per-site root mean square deviation (RMSD) between the echinoderm structures (1hlb, 1hlm) and the Ngb (1oj6) and Cygb (1urv) structures. Colored blocks at the bottom indicate charged (red) and non-charged (black) residues. Gaps are shown in grey, and the proximal and distal histidines are shown in blue. Helix locations are annotated above, and names using standard conventions. Overall RMSD for each plot is computed as the mean of the squared contributions from each site, and is indicated by the dashed red line. The blue areas underneath indicate the confidence associated with each column in the multiple alignment, as outputted by StructAlign.

To examine these features in further detail, we conducted individual pairwise comparisons with the two echinoderm structures (Fig 8). These analyses illustrate that the two structures differ significantly in their similarity to Ngb and Cygb, with the structure 1hlm showing a higher overall deviation to both structures (2.04Å and 1.81Å vs. 1.48Å and 1.30Å for 1hlb). There are also more localized differences, such as the large divergence in 1hlm with respect to 1oj6 at the N-terminal end of helix F, and the divergence with respect to both 1oj6 and 1urv at the C-terminal ends of helices G and H. It should also be noted that the D helix is missing in 1hlm, due to the shifted position of the heme group [42].

In summary, while the sequence-only trees place these two echinoderms very close to each other in the tree, there are clear differences in the structures of these proteins, which affect their relative structural similarity to other clades in the tree. Nevertheless, the joint sequence-structure model in StructAlign consistently places these globins between the plant and cytoglobin clades with high posterior probability, providing additional evidence to support the placement of the Group A echinoderms at this position in the tree.

## Discussion

### Properties of echinoderm hemoglobins

The only echinoderm Hbs that have been studied previously are the coelomic Hbs of sea cucumbers (*C*. *arenicola*, *P*. *chilensis*) and of brittle stars (*H*. *cordifera [elongata]*, *O*. *simplex*). It is worth noting that only 3 species out of some 2,000 existing brittle star species have been comprehensively investigated and shown to possess coleomic Hb [28]. The coelomocytes circulate in the water vascular system (WVS) [42, 44–46], and are anucleate in the brittle star *H*. *cordifera*. Although at least 3 Hbs all having a mass of ca. 16,000 were observed in *H*. *cordifera* [34], only two Hbs were found in the coelomocytes of another brittle star *O*. *simplex* [27]. The moderate oxygen affinity and cooperativity of oxygen binding of both are consonant with a proposed oxygen transport role.

In holothurians (sea cucumbers), the Hbs are contained within nucleated coelomocytes present within the body cavities, including perivisceral coelom, WVS, and hemal system [37, 44]. In general, the sea cucumbers appear to have several Hbs, with *C*. *arenicola* expressing as many as a dozen [38, 44]. Holothurian Hbs exhibit ligand-linked association, existing as homo- or heterodimers when oxygenated and aggregating into tetramers and possibly higher order polymers upon deoxygenation [30, 37, 38], leading to minimal cooperativity, whose physiological role remains unclear. The moderate oxygen binding affinities are again concordant with a transport/storage function [37]. Nothing is known about the biochemical properties of sea urchin and starfish Hbs.

### Relationships between vertebrate and echinoderm Hbs

The Bayesian tree based on a MAFFT MSA of echinoderm Hbs (Fig 1), shows them to split into two approximately equal groups of sequences. The trees based on MAFFT, T-CoffeeExpresso and Clustal Omega MSAs of echinoderm and vertebrate Hbs, provided in Figs 2, 3 and 4, respectively, clearly demonstrate the approximately equal division of echinoderm sequences into vertebrate Ngb-like and Cygb-like groups. The previously reported phylogeny of the vertebrate Hbs [14] is reproduced in all our trees, and the sea urchin single domain Adgb (marked by an arrow) is consistently placed next to the Ngb, as found previously [18]. Along with its homologs in the other echinoderm genomes, the *S*. *purpuratus* 166 residue Hb (XP_003729167.1), marked by a star in Figs 2, 3 and 4, is placed in the Cygb lineage (shaded grey), while the 2146 residue multidomain Hb (XP_001199205.2) and the 416 residue 2-domain Hb (XP_003725467.1) cluster with the Ngbs (shaded light blue). The two multidomain Hbs are reminiscent of some arthropod Hbs, such as the 9-domain Hb of the brine shrimp *Artemia* and the 2-domain Hbs of the cladoceran *Daphnia* [61], whose affiliation to vertebrate Hbs remains to be determined. Recent studies of vertebrate globin phylogeny by Hoffman, Storz and Opazo have shown that in addition to Ngb, there are four distinct vertebrate-specific lineages: (A) Cygb and cyclostome Hbs (Cyc Hbs) (B) Mb and GbE, (C) GbY, and (D), the Hb α- and β-globins [13–17]. We recover the latter four clades in our trees (Figs 2, 3 and 4). GbY is marked by a cross, and the remaining clades are shaded in grey. The brittle star coelomic (erythroid) Hbs cluster separately from the sea cucumber coelomic Hbs, suggesting an independent evolution from a Cygb-like globin lineage. Storz et al. have suggested a convergent cooption scenario for the evolution of erythroid oxygen transport in cyclostomes and jawed vertebrates, i.e. independent evolution of red cell Hbs in both groups [14]. The coelomic Hbs of *C*. *arenicola* and *P*. *chilensis* are placed close to Cygb in all the trees we generated, supporting the hypothesis that the erythroid oxygen binding function may have evolved independently in echinoderms from a hexacoordinate Cygb.

Although the Ngb-like echinoderm globins are clearly identified, the remaining echinoderm globins appear to be affiliated only with lineage A of Hoffman et al, the Cygb + Cyc Hb globin lineage. No echinoderm globin clusters with any of the remaining three lineages, Mb + GbE, GbY, or the Hb α- and β-globins. Hence, it remains to be established whether the four lineages identified in vertebrates and cyclostomes occur also in echinoderms. The presence of echinoderm globins related to vertebrate Ngbs and Cygbs, evidence for the Ngb and Cygb lineages in echinoderms, suggests that the emergence of the Cygb and Ngb lineages occurred in the ancestor shared by the vertebrates and echinoderms.

## Methods

### Identification of globin sequences

Putative globins and globin domains were identified from the SUPERFAMILY globin gene assignments (http://supfam.mrc-lmb.cam.ac.uk) [62], and via BLASTP and psiblasT [63], and tblastn searches of the GenBank protein and nucleotide databases. All putative globins were subjected to a FUGUE search [64] (http://www-cryst.bioc.cam.ac.uk), a stringent test of whether a given sequence is a globin [65–68]. Given a query sequence, FUGUE scans a database of structural profiles, calculates the sequence-structure compatibility scores for each entry, using environment-specific substitution tables and structure-dependent gap penalties, and produces a list of potential homologs and alignments. FUGUE assesses the similarity between the query and a given structure via the Z score, the number of standard deviations above the mean score obtained by chance: the default threshold Z = 6.0 corresponds to 99% probability [64].

### Multiple sequence alignment and myoglobin-fold criteria

MSA’s were carried out using some of the following algorithms: PROBCONS [69], MAFFT 6.833 [70], Clustal Omega [71], T-Coffee Expresso [72], and MUSCLE 3.7 [73], available at EMBL-EBI (www.ebi.ac.uk/Tools/). The resulting alignments were checked manually for the conservation of the F8 His. Their quality was assessed using GUIDANCE [74]. This algorithm assesses the quality of the MSA via a GUIDANCE score that reflect the robustness of an alignment to guide-tree uncertainty [75]. Furthermore, it also allows the removal of columns from the MSA below a cutoff score (default 0.93). We subjected our MSA’s to several rounds of column trimming until the MSA Guidance scores converged.

All known globin sequences exhibit the Mb-fold [49], the pattern of predominantly hydrophobic residues at 36 conserved, solvent-inaccessible positions, including 33 intra-helical residues defining helices A through H (A8, A11-12, A15, B6, B9-10, B13-14, C4, E4, E7-8, E11-12, E15, E18-19, F1, F4, G5, G8, G11-12-13, G15-16, H7-8, H11-12, H15, and H19), the two inter-helical residues at CD1 and FG4, and the invariant His at F8. Our criteria for a satisfactory globin required a FUGUE Z score >6, a His at the proximal F8 position and presence of helices BC through G.

### Molecular phylogeny

Bayesian inference analyses were carried out employing MrBayes version 3.2.2 [76], and the WAG model of amino acid evolution [77], assuming a gamma distribution of evolution rates, as indicated by a ProtTest analysis of the alignment [78]. Two parallel runs, each consisting of four chains were run simultaneously for up to 10x10^6^ generations and trees were sampled every 1000 generations. The final average standard deviations of split frequencies were stationary in all analyses and posterior probabilities were estimated on the final 60–80% trees. The CIPRES web portal was used for the Bayesian analyses [79]. In all Bayesian trees (Figs 1, 2, 3 and 4) support values at branches represent Bayesian posterior probabilities (>0.5). The sequences are identified by the first 3 letters of the genus and species parts of the binomial, the number of residues, globin subfamily if known and a 3 letter abbreviation of the Echinoderm class (AST—Asteroid; ECH—Echiuroid; HOL—Holothuroid; OPH—Ophiuroid).

Maximum likelihood-based (ML) phylogenetic analyses were performed using RAxML version 7.2.3 [80] assuming the WAG model and gamma distribution of substitution rates. The resulting trees were tested by bootstrapping with 100 replicates. Neighbor joining (NJ) analyses were performed using MEGA version 5.2 [81]. Distances were corrected for superimposed events using the Poisson method. All positions containing alignment gaps and missing data were eliminated only in pairwise sequence comparisons (pairwise deletion option). The reliability of the branching pattern was tested by bootstrap analysis with 1000 replications.

In carrying out analyses of echinoderm sequences with globins from other metazoans, we used at least 3 sequences as representatives of each of the Hb subfamilies. For example in the case of vertebrate globins we used at least 3 sequences to represent the Ngbs, Cygbs, Mbs, HbA, HbB, etc: primate, rodent, bird, reptile, amphibian, fish (ray-finned and sharks) and the cyclostome Hbs (lamprey, hagfish) (S3 Table).

### Structural analysis

Analyses using the Bayesian model for the joint evolution of sequence and structure were carried out using the StructAlign plugin [59, 60], as part of the StatAlign package [60]. Four independent MCMC chains were run, starting from different initial configurations. The smaller dataset was run for a burn-in period of 1m iterations, followed by a sampling period of 1m iterations, generating 5,000 samples at intervals of 200 iterations for alignments, trees, and model parameters; the larger structural dataset was run for a burn-in period of 10m iterations, and a sampling period of 50m iterations, generating 250,000 samples at intervals of 200 iterations. The sampled trees were used to generate majority consensus trees.

Per-site root-mean square deviation was computed based on the summary alignment generated by StructAlign. For two structures *A* and *B*, this is calculated using the following expression

$$dRMSD_{i}\left(A,B\right)=\sqrt{\frac{\text{1}}{n-\text{1}}\sum_{j}{\left[d_{ij}\left(A\right)-d_{ij}\left(B\right)\right]}^{\text{2}}}$$

Where *d*_*ij*_(*A*) is the distance between alpha carbons for residues *i* and *j* in structure *A*, and *n* is the number of aligned residues, with the sum running over all residues *j* that are aligned between the two structures. Overall RMSD was computed as the root of the mean of the squared contributions for each alignment column. Use of interresidue distances rather than coordinates allows for the key structural differences to be highlighted without requiring a specific choice of rotational superposition for the structures.

## Supporting Information

S1 Fig A MAFFT L-INS-i alignment of the 14 domains of the multidomain Hb (XP_001199205.2) and the two domains of the 416 residue Hb (XP_003725467) from the sea urchin *Strongylocentrotus purpuratus* with sperm whale Mb (1A6M). The Mb fold template [49] consists of predominantly hydrophobic residues at 37 positions, defining helices A through H: A8, A11, A12, A15, B6, B9, B10, B13, B14, C5, CD1, CD4, E4, E7, E8, E11, E12, E15, E18, E19, F1, F4, F8, FG4, G5, G8, G11, G12, G13, G15, G16, H7, H8, H11, H12, H15, and H19. The distal His at E7 and the proximal His at F8 are in red.
(DOC)

S2 Fig A similarity matrix for 52 echinoderm Hbs, based on a MAFFT L-INS-i alignment. (DOC)

S3 Fig A diagrammatic representation of vertebrate phylogeny from ref [16]. (PDF)

S4 Fig A Structural superposition of structures 1urv (red) and 2dc3 (cyan) using StructAlign. (BMP)

S1 Table Echinoderm species with genomes/transcriptomes showing hits with homologs of the *S*. *purpuratus* globins. (DOC)

S2 Table Intron locations in echinoderm Hbs. (DOCX)

S3 Table Vertebrate, plant and outgroup sequences used in the phylogenetic analyses. (DOC)
