# Supplementary material for: Phylogeny of Echinoderm Hemoglobins
Source: PLoS One. 2015 Aug 6;10(8):e0129668. doi: 10.1371/journal.pone.0129668 (PMC4527676; doi:10.1371/journal.pone.0129668)
Supplement: S1 Fig — The Mb fold template [49] consists of predominantly hydrophobic residues at 37 positions, defining helices A through H: A8, A11, A12, A15, B6, B9, B10, B13, B14, C5, CD1, CD4, E4, E7, E8, E11, E12, E15, E18, E19, F1, F4, F8, FG4, G5, G8, G11, G12, G13, G15, G16, H7, H8, H11, H12, H15, and H19. The distal His at E7 and the proximal His at F8 are in red. (DOC) [file pone.0129668.s001.doc]

**aaaaaaaaaAaaAAaaAa..bbbbBbbBBbbBBbbcCcccccDddD....dddddddeeeEeeEEeeEEeeEeeEEe............FffFfffFff...ggggGggggGggGGGgGGggg**

**Mb fold ...|..||..|.....|....||..||...|.....|..|..............|..||..||..|..||.............|..|...|.........|....|..|||.||...**

**1A6M : -VLSEGEWQLVLHVWAKVEADVAGHGQDILIRLFKSHPETLEKFDRFKHLKTEAEMKASEDLKKHGVTVLTALGAILKKKGHHEAELKPLAQSHATKH---KIPIKYLE--FISEAIIHVLHSRHP- : 120
Strpur_416_D1 : AGLDERQKTLVENTWKTLEKNTELYGSIMFAKLTTDHPDIGKLFPFGGKNLTYGELLVDPDVRVHGKRVIETLGSVVEDLDDMEL-VIQILEDLGQRHNAYNAKKTHII--AVGGALLFTIEEALG- : 123
Strpur_416_D2 : --LRAREQELVQKTWGVLSLDTEQHGAAMFAKLISAHPAVAQMFPFG-ENLSYSQLVQNPTLRAHGKRVMETIGQTVGSLDDLDI-LVPILRDLARRHVGYSVTRQHFEVRP--------------- : 108
Strpur_2146_D1 : EVMSTTQKRLIQDTWKKLSKDPERHGSVMFAKLTTSYPEVGKVFPFGGKGFSYEQLLRNRDVKAHGRRVFETVGQAINGLNDLNL-LMPTLKDLAQRHVGYNVQKRYFV--PTGEAFLHAVRLGVGP : 124
Strpur_2146_D2 : GPITATHKSLVQKSWKTIGQDPTKHGSVMFARLITDNPHVGKLFPFGSMNLSYDQLLANKDLGAHGKRIIDTIGVAVSGLDDLEL-LIRILQDLAKRHVGYNVTKQHFK--PVGGALIHALRQGLG- : 123
Strpur_2146_D3 : -GLTTEQKNLIKGSWRSVAPEKAKCGAVMFAKLVTEHPHIGRLFPFGGLSKSYQSLLMDDKVKAHGLRVMQTISDAIRVLDNLDK-LLPLFRDLGKRHVAYGVTKEHFA--AVGRALMNSLKETLG- : 122
Strpur_2146_D4 : GLITRNQKEIVQRTWKMLEKDPGRHGAVMFARLLTDHPDVGHLFPFGNKGLSYHQLLWDDTVKAHGKRVMQTVGHAVDGLNDLDV-LVPILQDLARRHIEYNVNKEHFE--PVGKALLYAIEKGLG- : 123
Strpur_2146_D5 : GPLTREQKRLVKTTWKKLATNPTKHGAVMFAKLTTENPDVGHMFPFGGKNLSYQQLLKDPQAQAHGKRVMETVGTAVDGLDDLDL-LVPILRELATRHVGYKVTKQHFK--PVGAALIHAIKEGLG- : 123
Strpur_2146_D6 : GPLTSAQKELVKRTWQVLAPNPAKHGAVMFAQLTTENPDVGHLFPFGGKNLSYQQLLKDPQAQAHGKRVMETVGTAVDGLDDLDL-LVPILRELAMRHVGYKVTKQHFS--AVGAALIHAIKEGLG- : 123
Strpur_2146_D7 : GPLTSAQKELVKRTWQVLAPNSAKHGAVMFAKLTTENPDVGHLFPFGGKNLSYQQLLKDPQAQAHGKRVMETVGTAVNGLDDLEL-LVPILRELATRHIGYKVTKQHFS--AVGAALIHAIKEGLG- : 123
Strpur_2146_D8 : GPLTSVQKELVKKTWQVLAPNPVKHGAVMFAKLTTENPDVGHLFPFGGKNLSYQQLLKDPQAQAHGKRVMETVGTAVEGLDDLDL-LVPILQELATRHIGYKVTKQHFS--AVGAALIHAIKEGLG- : 123
Strpur_2146_D9 : GPLTSAQKELVKRTWQVLAPNPAKHGAVMFAKLLTRHPNVGKLFPFGKEDLSYEQLLKHAQVQAHGKKVMEKVGDAVDGLDDLDL-LVPILKELGGRHVGYGVNKQLFE--PVGEVLLETIKEALG- : 123
Strpur_2146_D10 : GSITAEHKRLVQKTWTKLSSNPAKHGATMFSKLVTDYPAVGTLLPFGNEGLSYDQLLVDPRVRAHGTKVMQTVGSAVDRLNDLES-VVPLLQELATRHISYGVTKQHFS--PVVESLMHAIKQGLD- : 123
Strpur_2146_D11 : GSLDDAKINLVRETWVTLSTNPEQHGAVMFAKLVTDNPTVGRLFPFGKKNLSYDQLLVDPQVKSHGKRVMDTVGHAVAGLDDLDL-LVPILEELAQRHHLYGVTKKNFK--PVGDALMHAIEKGLG- : 123
Strpur_2146_D12 : TILTQETKQLVKESWKILSTDPGKHGAVMFARLTTQNPIVGRLFPFGDKNLSYGQLLSNRMVREHGTKVMKTIGQAVDTLDNVDN-LVSALKDLGLRHTQYGVTKMHFE--PVGQALIYAIKEGLG- : 123
Strpur_2146_D13 : GPISSNHIELLKRTWKVLGQNPEQLGAVLFAKLASDHPLVSSLFPFGGKGLTYDQLLQNDDVKKHGRQFMATVGNAIENLENHEI-LIPMFEDLAKRHAGFGVTRQHIP--FAGEALLYAISDALG- : 123
Strpur_2146_D14 : DTMTKKQRELVESTWEMMASAPDKHGAVMFAKLVSDNPKVGRLFPFGKKKYPYKKLLNENEVISHGERFMTTLGQVVGGLDDPEF-LVPMLHQRTTRHSGYGVTKELFL--AVKSALMFTLKQGLGK : 124
Strpur_166 : TGLTKQQKALIKKSWTYVLEDKLRIGVIIFIKLFKAFPASQQLFEKLKDYTDFEELARNKKMKAHATRVMAALTSLVENIDQPDI-LDELLRNTSVTHYRMRMPPHYFE--DLGGVIIEALVENLG- : 123**

**hhhhhhHHhhHHhhHhhhHhhhhhhh**

**Mb fold ||..||..|...|..**

**1A6M : GDFGADAQGAMNKALELFRKDIAAKYKELGY : 151
Strpur_416_D1 : AGFTPEVKAAWAAVYNIVSDTMSTKLSGGPL : 154
Strpur_416_D2 : ------------------------------- : -
Strpur_2146_D1 : SSFTSDVRDAWAVLFKVVTDPMAEVVPD--- : 152
Strpur_2146_D2 : SRFTPELEAAWTAVYSVVADTMSPLLPAGGL : 154
Strpur_2146_D3 : SSFNDNTKNAWLTFWQLIEDTMSEQLVAVAE : 153
Strpur_2146_D4 : NTFDNETKEAWMAVFSIIVNTMSEILPSSTE : 154
Strpur_2146_D5 : SSYSTDIQSAWVAVFQLITDTMSDVLPNS-- : 152
Strpur_2146_D6 : SSYSPDIQSAWVAVFQLITNTMSDVL----- : 149
Strpur_2146_D7 : SSYSPDIQSAWVAVFQLITDTMSDVL----- : 149
Strpur_2146_D8 : SSYSPDIQGAWVAVFQLITNTMSDVLPDS-- : 152
Strpur_2146_D9 : DTFNEELRLAWTAVFKIISDTMSEPLNED-- : 152
Strpur_2146_D10 : SDYNNDVEKSWLAVLQVIVDTMSAELPD--- : 151
Strpur_2146_D11 : QRFTTEVEGAWKAVFTVITNTMSKILPEN-- : 152
Strpur_2146_D12 : QSFTSKVKGAWSSIFQLISETMSEELPE--- : 151
Strpur_2146_D13 : QDFTDDVRQAWETVFQLIVDVMSAKLPDGKK : 154
Strpur_2146_D14 : SVFSGEVQEAWAAAYQFIEDSMCEKL----- : 150
Strpur_166 : DKFTPKTKEAWLIYYGYMCRIMLEEMEELEP : 154**
