# Supplementary material for: Phylogeny of Echinoderm Hemoglobins
Source: PLoS One. 2015 Aug 6;10(8):e0129668. doi: 10.1371/journal.pone.0129668 (PMC4527676; doi:10.1371/journal.pone.0129668)
Supplement: S2 Fig — (DOC) [file pone.0129668.s002.doc]

Supplemental Fig. 2 .SIMILARITY MATRIX for A MAFFT L-INS-i alignment of the 52 Echinoderm Hbs, obtained using the Expasy decrease redundancy tool (Web.Expasy.Org/Decrease_ Redundancy/‎), set at 90% identity.

Ampfil_121_OPH_**JG390935.1** Vs Ampfil_165_OPH_**JG391025.1** : 98.35 % id

Ampfil_121_OPH_JG390935.1 Vs Apojap_217_Hb1_HOL : 27.97 % id

Ampfil_121_OPH_JG390935.1 Vs Apojap_167_Hb2_HOL : 30.21 % id

Ampfil_121_OPH_JG390935.1 Vs Apojap_217_Hb3_HOL : 27.97 % id

Ampfil_121_OPH_JG390935.1 Vs Apojap_167_Hb4 : 30.21 % id

Ampfil_121_OPH_JG390935.1 Vs Astrub_174_Hb1_AST : 15.97 % id

Ampfil_121_OPH_JG390935.1 Vs Astrub_175_Hb2_AST : 21.01 % id

Ampfil_121_OPH_JG390935.1 Vs Cauare_159_HOL_**P80018.3** : 26.27 % id

Ampfil_121_OPH_JG390935.1 Vs Cauare_159_HOL_**P80017.3** : 26.27 % id

Ampfil_121_OPH_JG390935.1 Vs Hemcor_144_Hb1_AST : 28.30 % id

Ampfil_121_OPH_JG390935.1 Vs Hemcor_144_Hb2_AST : 32.08 % id

Ampfil_121_OPH_JG390935.1 Vs Labann_206_AST : 30.25 % id

Ampfil_121_OPH_JG390935.1 Vs Labann_263_AST : 22.61 % id

Ampfil_121_OPH_JG390935.1 Vs Ophisim_158_Hba_AST : 33.91 % id

Ampfil_121_OPH_JG390935.1 Vs Ophsim_144_Hbb_AST : 29.25 % id

Ampfil_121_OPH_JG390935.1 Vs Parliv_229_ECH_**AM524155.1** : 31.58 % id

Ampfil_121_OPH_JG390935.1 Vs Parchi_158_HOL_**P15161.2** : 27.12 % id

Ampfil_121_OPH_JG390935.1 Vs Patmin_1144_Hb1_AST : 23.28 % id

Ampfil_121_OPH_JG390935.1 Vs Patmin_142_Hb2_AST : 16.07 % id

Ampfil_121_OPH_JG390935.1 Vs Pisoch_516_D1_AST : 22.41 % id

Ampfil_121_OPH_JG390935.1 Vs Pisoch_516_D2_AST : 21.93 % id

Ampfil_121_OPH_JG390935.1 Vs Remgou_206_AST : 30.25 % id

Ampfil_121_OPH_JG390935.1 Vs Psafra_138_CRI : 9.43 % id

Ampfil_121_OPH_JG390935.1 Vs Psicha_196_AST : 32.77 % id

Ampfil_121_OPH_JG390935.1 Vs Ptetes_D1_468_AST : 21.74 % id

Ampfil_121_OPH_JG390935.1 Vs Ptetes_D2_468_AST : 22.69 % id

Ampfil_121_OPH_JG390935.1 Vs Strpur_166_ECH_**XP_003729167.1** : 32.77 % id

Ampfil_121_OPH_JG390935.1 Vs Allfra_167_ECH : 31.93 % id

Ampfil_121_OPH_JG390935.1 Vs Psedep_155_ECH : 33.33 % id

Ampfil_121_OPH_JG390935.1 Vs Strdro_167_ECH : 31.93 % id

Ampfil_121_OPH_JG390935.1 Vs Strint¬167_ECH : 33.61 % id

Ampfil_121_OPH_JG390935.1 Vs Strnud_146_ECH : 35.29 % id

Ampfil_121_OPH_JG390935.1 Vs Strpal_167_ECH : 28.57 % id

Ampfil_121_OPH_JG390935.1 Vs Strpur_416_D1_**XP_003725467.1** : 26.09 % id

Ampfil_121_OPH_JG390935.1 Vs Strpur_416_D2_**XP_003725467.1** : 28.57 % id

Ampfil_121_OPH_JG390935.1 Vs Strpur_2146_D1_**XP_001199205.2** : 24.58 % id

Ampfil_121_OPH_JG390935.1 Vs Strpur_2146_D2_**XP_001199205.2** : 20.87 % id

Ampfil_121_OPH_JG390935.1 Vs Strpur_2146_D3_**XP_001199205.2** : 27.19 % id

Ampfil_121_OPH_JG390935.1 Vs Strpur_2146_D4_**XP_001199205.2** : 26.09 % id

Ampfil_121_OPH_JG390935.1 Vs Strpur_2146_D5_**XP_001199205.2** : 22.61 % id

Ampfil_121_OPH_JG390935.1 Vs Strpur_2146_D6_**XP_001199205.2** : 23.48 % id

Ampfil_121_OPH_JG390935.1 Vs Strpur_2146_D7_**XP_001199205.2** : 23.48 % id

Ampfil_121_OPH_JG390935.1 Vs Strpur_2146_D8_**XP_001199205.2** : 24.35 % id

Ampfil_121_OPH_JG390935.1 Vs Strpur_2146_D9_**XP_001199205.2** : 26.09 % id

Ampfil_121_OPH_JG390935.1 Vs Strpur_2146_D10_**XP_001199205.2** : 18.26 % id

Ampfil_121_OPH_JG390935.1 Vs Strpur_2146_D11_**XP_001199205.2** : 26.09 % id

Ampfil_121_OPH_JG390935.1 Vs Strpur_2146_D12_**XP_001199205.2** : 23.48 % id

Ampfil_121_OPH_JG390935.1 Vs Strpur_2146_D13_**XP_001199205.2** : 27.83 % id

Ampfil_121_OPH_JG390935.1 Vs Strpur_2146_D14_**XP_001199205.2** : 22.03 % id

Ampfil_121_OPH_JG390935.1 Vs Strpur_267_Adgb_**XP_001195639.2** : 15.32 % id

Ampfil_121_OPH_JG390935.1 Vs Xyljan_537_AST : 20.87 % id

Ampfil_165_OPH_JG391025.1 Vs Apojap_217_Hb1_HOL : 28.40 % id

Ampfil_165_OPH_JG391025.1 Vs Apojap_167_Hb2_HOL : 30.00 % id

Ampfil_165_OPH_JG391025.1 Vs Apojap_217_Hb3_HOL : 28.40 % id

Ampfil_165_OPH_JG391025.1 Vs Apojap_167_Hb4 : 30.00 % id

Ampfil_165_OPH_JG391025.1 Vs Astrub_174_Hb1_AST : 18.30 % id

Ampfil_165_OPH_JG391025.1 Vs Astrub_175_Hb2_AST : 20.13 % id

Ampfil_165_OPH_JG391025.1 Vs Cauare_159_HOL_P80018.3 : 27.45 % id

Ampfil_165_OPH_JG391025.1 Vs Cauare_159_HOL_P80017.3 : 24.18 % id

Ampfil_165_OPH_JG391025.1 Vs Hemcor_144_Hb1_AST : 32.87 % id

Ampfil_165_OPH_JG391025.1 Vs Hemcor_144_Hb2_AST : 34.27 % id

Ampfil_165_OPH_JG391025.1 Vs Labann_206_AST : 32.50 % id

Ampfil_165_OPH_JG391025.1 Vs Labann_263_AST : 21.33 % id

Ampfil_165_OPH_JG391025.1 Vs Ophisim_158_Hba_AST : 37.25 % id

Ampfil_165_OPH_JG391025.1 Vs Ophsim_144_Hbb_AST : 32.87 % id

Ampfil_165_OPH_JG391025.1 Vs Parliv_229_ECH_AM524155.1 : 31.37 % id

Ampfil_165_OPH_JG391025.1 Vs Parchi_158_HOL_P15161.2 : 27.63 % id

Ampfil_165_OPH_JG391025.1 Vs Patmin_1144_Hb1_AST : 25.00 % id

Ampfil_165_OPH_JG391025.1 Vs Patmin_142_Hb2_AST : 19.72 % id

Ampfil_165_OPH_JG391025.1 Vs Pisoch_516_D1_AST : 21.88 % id

Ampfil_165_OPH_JG391025.1 Vs Pisoch_516_D2_AST : 20.13 % id

Ampfil_165_OPH_JG391025.1 Vs Remgou_206_AST : 32.50 % id

Ampfil_165_OPH_JG391025.1 Vs Psafra_138_CRI : 8.70 % id

Ampfil_165_OPH_JG391025.1 Vs Psicha_196_AST : 33.75 % id

Ampfil_165_OPH_JG391025.1 Vs Ptetes_D1_468_AST : 20.25 % id

Ampfil_165_OPH_JG391025.1 Vs Ptetes_D2_468_AST : 23.38 % id

Ampfil_165_OPH_JG391025.1 Vs Strpur_166_ECH_XP_003729167.1 : 28.83 % id

Ampfil_165_OPH_JG391025.1 Vs Allfra_167_ECH : 28.22 % id

Ampfil_165_OPH_JG391025.1 Vs Psedep_155_ECH : 29.03 % id

Ampfil_165_OPH_JG391025.1 Vs Strdro_167_ECH : 28.22 % id

Ampfil_165_OPH_JG391025.1 Vs Strint¬167_ECH : 29.45 % id

Ampfil_165_OPH_JG391025.1 Vs Strnud_146_ECH : 30.14 % id

Ampfil_165_OPH_JG391025.1 Vs Strpal_167_ECH : 24.54 % id

Ampfil_165_OPH_JG391025.1 Vs Strpur_416_D1_XP_003725467.1 : 26.58 % id

Ampfil_165_OPH_JG391025.1 Vs Strpur_416_D2_XP_003725467.1 : 30.56 % id

Ampfil_165_OPH_JG391025.1 Vs Strpur_2146_D1_XP_001199205.2 : 24.68 % id

Ampfil_165_OPH_JG391025.1 Vs Strpur_2146_D2_XP_001199205.2 : 23.42 % id

Ampfil_165_OPH_JG391025.1 Vs Strpur_2146_D3_XP_001199205.2 : 25.32 % id

Ampfil_165_OPH_JG391025.1 Vs Strpur : 27.56 % id

Ampfil_165_OPH_JG391025.1 Vs Strpur : 26.32 % id

Ampfil_165_OPH_JG391025.1 Vs Strpur_2146_D6_XP_001199205.2 : 26.32 % id

Ampfil_165_OPH_JG391025.1 Vs Strpur_2146_D7_XP_001199205.2 : 26.32 % id

Ampfil_165_OPH_JG391025.1 Vs Strpur_2146_D8_XP_001199205.2 : 26.97 % id

Ampfil_165_OPH_JG391025.1 Vs Strpur_2146_D9_XP_001199205.2 : 28.95 % id

Ampfil_165_OPH_JG391025.1 Vs Strpur_2146_D10_XP_001199205.2 : 22.52 % id

Ampfil_165_OPH_JG391025.1 Vs Strpur_2146_D11_XP_001199205.2 : 27.63 % id

Ampfil_165_OPH_JG391025.1 Vs Strpur_2146_D12_XP_001199205.2 : 25.17 % id

Ampfil_165_OPH_JG391025.1 Vs Strpur_2146_D13_XP_001199205.2 : 25.16 % id

Ampfil_165_OPH_JG391025.1 Vs Strpur_2146_D14_XP_001199205.2 : 26.32 % id

Ampfil_165_OPH_JG391025.1 Vs Strpur_267_Adgb_XP_001195639.2 : 16.77 % id

Ampfil_165_OPH_JG391025.1 Vs Xyljan_537_AST : 22.22 % id

Apojap_217_Hb1_HOL Vs Apojap_167_Hb2_HOL : 100.00 % id

Apojap_217_Hb1_HOL Vs Apojap_217_Hb3_HOL : 100.00 % id

Apojap_217_Hb1_HOL Vs Apojap_167_Hb4 : 100.00 % id

Apojap_217_Hb1_HOL Vs Astrub_174_Hb1_AST : 16.86 % id

Apojap_217_Hb1_HOL Vs Astrub_175_Hb2_AST : 23.12 % id

Apojap_217_Hb1_HOL Vs Cauare_159_HOL_P80018.3 : 39.62 % id

Apojap_217_Hb1_HOL Vs Cauare_159_HOL_P80017.3 : 37.74 % id

Apojap_217_Hb1_HOL Vs Hemcor_144_Hb1_AST : 29.58 % id

Apojap_217_Hb1_HOL Vs Hemcor_144_Hb2_AST : 32.39 % id

Apojap_217_Hb1_HOL Vs Labann_206_AST : 38.38 % id

Apojap_217_Hb1_HOL Vs Labann_263_AST : 23.49 % id

Apojap_217_Hb1_HOL Vs Ophisim_158_Hba_AST : 29.49 % id

Apojap_217_Hb1_HOL Vs Ophsim_144_Hbb_AST : 26.76 % id

Apojap_217_Hb1_HOL Vs Parliv_229_ECH_AM524155.1 : 40.79 % id

Apojap_217_Hb1_HOL Vs Parchi_158_HOL_P15161.2 : 39.24 % id

Apojap_217_Hb1_HOL Vs Patmin : 24.50 % id

Apojap_217_Hb1_HOL Vs Patmin : 27.66 % id

Apojap_217_Hb1_HOL Vs Pisoch_516_D1_AST : 21.56 % id

Apojap_217_Hb1_HOL Vs Pisoch_516_D2_AST : 24.32 % id

Apojap_217_Hb1_HOL Vs Remgou_206_AST : 38.38 % id

Apojap_217_Hb1_HOL Vs Psafra_138_CRI : 9.49 % id

Apojap_217_Hb1_HOL Vs Psicha_196_AST : 36.76 % id

Apojap_217_Hb1_HOL Vs Ptetes_D1_468_AST : 24.20 % id

Apojap_217_Hb1_HOL Vs Ptetes_D2_468_AST : 26.09 % id

Apojap_217_Hb1_HOL Vs Strpur_166_ECH_XP_003729167.1 : 41.82 % id

Apojap_217_Hb1_HOL Vs Allfra_167_ECH : 40.36 % id

Apojap_217_Hb1_HOL Vs Psedep_155_ECH : 40.26 % id

Apojap_217_Hb1_HOL Vs Strdro_167_ECH : 40.96 % id

Apojap_217_Hb1_HOL Vs Strint¬167_ECH : 42.77 % id

Apojap_217_Hb1_HOL Vs Strnud_146_ECH : 39.31 % id

Apojap_217_Hb1_HOL Vs Strpal_167_ECH : 35.54 % id

Apojap_217_Hb1_HOL Vs Strpur_416_D1_XP_003725467.1 : 26.75 % id

Apojap_217_Hb1_HOL Vs Strpur_416_D2_XP_003725467.1 : 26.17 % id

Apojap_217_Hb1_HOL Vs Strpur_2146_D1_XP_001199205.2 : 23.53 % id

Apojap_217_Hb1_HOL Vs Strpur_2146_D2_XP_001199205.2 : 29.94 % id

Apojap_217_Hb1_HOL Vs Strpur_2146_D3_XP_001199205.2 : 28.76 % id

Apojap_217_Hb1_HOL Vs Strpur : 27.74 % id

Apojap_217_Hb1_HOL Vs Strpur : 25.83 % id

Apojap_217_Hb1_HOL Vs Strpur_2146_D6_XP_001199205.2 : 26.49 % id

Apojap_217_Hb1_HOL Vs Strpur_2146_D7_XP_001199205.2 : 26.49 % id

Apojap_217_Hb1_HOL Vs Strpur_2146_D8_XP_001199205.2 : 27.15 % id

Apojap_217_Hb1_HOL Vs Strpur_2146_D9_XP_001199205.2 : 26.49 % id

Apojap_217_Hb1_HOL Vs Strpur_2146_D10_XP_001199205.2 : 26.67 % id

Apojap_217_Hb1_HOL Vs Strpur_2146_D11_XP_001199205.2 : 28.48 % id

Apojap_217_Hb1_HOL Vs Strpur_2146_D12_XP_001199205.2 : 28.00 % id

Apojap_217_Hb1_HOL Vs Strpur_2146_D13_XP_001199205.2 : 25.32 % id

Apojap_217_Hb1_HOL Vs Strpur_2146_D14_XP_001199205.2 : 25.83 % id

Apojap_217_Hb1_HOL Vs Strpur_267_Adgb_XP_001195639.2 : 8.81 % id

Apojap_217_Hb1_HOL Vs Xyljan_537_AST : 26.32 % id

Apojap_167_Hb2_HOL Vs Apojap_217_Hb3_HOL : 100.00 % id

Apojap_167_Hb2_HOL Vs Apojap_167_Hb4 : 100.00 % id

Apojap_167_Hb2_HOL Vs Astrub_174_Hb1_AST : 18.46 % id

Apojap_167_Hb2_HOL Vs Astrub_175_Hb2_AST : 25.19 % id

Apojap_167_Hb2_HOL Vs Cauare_159_HOL_P80018.3 : 42.75 % id

Apojap_167_Hb2_HOL Vs Cauare_159_HOL_P80017.3 : 39.69 % id

Apojap_167_Hb2_HOL Vs Hemcor_144_Hb1_AST : 28.00 % id

Apojap_167_Hb2_HOL Vs Hemcor_144_Hb2_AST : 29.60 % id

Apojap_167_Hb2_HOL Vs Labann_206_AST : 40.15 % id

Apojap_167_Hb2_HOL Vs Labann_263_AST : 22.90 % id

Apojap_167_Hb2_HOL Vs Ophisim_158_Hba_AST : 31.54 % id

Apojap_167_Hb2_HOL Vs Ophsim_144_Hbb_AST : 27.20 % id

Apojap_167_Hb2_HOL Vs Parliv_229_ECH_AM524155.1 : 41.48 % id

Apojap_167_Hb2_HOL Vs Parchi_158_HOL_P15161.2 : 42.31 % id

Apojap_167_Hb2_HOL Vs Patmin : 23.48 % id

Apojap_167_Hb2_HOL Vs Patmin : 26.98 % id

Apojap_167_Hb2_HOL Vs Pisoch_516_D1_AST : 20.95 % id

Apojap_167_Hb2_HOL Vs Pisoch_516_D2_AST : 23.85 % id

Apojap_167_Hb2_HOL Vs Remgou_206_AST : 40.15 % id

Apojap_167_Hb2_HOL Vs Psafra_138_CRI : 8.59 % id

Apojap_167_Hb2_HOL Vs Psicha_196_AST : 37.96 % id

Apojap_167_Hb2_HOL Vs Ptetes_D1_468_AST : 24.46 % id

Apojap_167_Hb2_HOL Vs Ptetes_D2_468_AST : 25.95 % id

Apojap_167_Hb2_HOL Vs Strpur_166_ECH_XP_003729167.1 : 41.43 % id

Apojap_167_Hb2_HOL Vs Allfra_167_ECH : 39.29 % id

Apojap_167_Hb2_HOL Vs Psedep_155_ECH : 41.43 % id

Apojap_167_Hb2_HOL Vs Strdro_167_ECH : 40.00 % id

Apojap_167_Hb2_HOL Vs Strint¬167_ECH : 42.14 % id

Apojap_167_Hb2_HOL Vs Strnud_146_ECH : 40.00 % id

Apojap_167_Hb2_HOL Vs Strpal_167_ECH : 36.43 % id

Apojap_167_Hb2_HOL Vs Strpur_416_D1_XP_003725467.1 : 25.90 % id

Apojap_167_Hb2_HOL Vs Strpur_416_D2_XP_003725467.1 : 25.27 % id

Apojap_167_Hb2_HOL Vs Strpur_2146_D1_XP_001199205.2 : 24.24 % id

Apojap_167_Hb2_HOL Vs Strpur_2146_D2_XP_001199205.2 : 30.22 % id

Apojap_167_Hb2_HOL Vs Strpur_2146_D3_XP_001199205.2 : 27.21 % id

Apojap_167_Hb2_HOL Vs Strpur : 29.20 % id

Apojap_167_Hb2_HOL Vs Strpur : 25.56 % id

Apojap_167_Hb2_HOL Vs Strpur_2146_D6_XP_001199205.2 : 26.32 % id

Apojap_167_Hb2_HOL Vs Strpur_2146_D7_XP_001199205.2 : 26.32 % id

Apojap_167_Hb2_HOL Vs Strpur_2146_D8_XP_001199205.2 : 27.07 % id

Apojap_167_Hb2_HOL Vs Strpur_2146_D9_XP_001199205.2 : 26.32 % id

Apojap_167_Hb2_HOL Vs Strpur_2146_D10_XP_001199205.2 : 27.27 % id

Apojap_167_Hb2_HOL Vs Strpur_2146_D11_XP_001199205.2 : 27.82 % id

Apojap_167_Hb2_HOL Vs Strpur_2146_D12_XP_001199205.2 : 26.52 % id

Apojap_167_Hb2_HOL Vs Strpur_2146_D13_XP_001199205.2 : 26.47 % id

Apojap_167_Hb2_HOL Vs Strpur_2146_D14_XP_001199205.2 : 26.15 % id

Apojap_167_Hb2_HOL Vs Strpur_267_Adgb_XP_001195639.2 : 9.66 % id

Apojap_167_Hb2_HOL Vs Xyljan_537_AST : 25.37 % id

Apojap_217_Hb3_HOL Vs Apojap_167_Hb4 : 100.00 % id

Apojap_217_Hb3_HOL Vs Astrub_174_Hb1_AST : 16.86 % id

Apojap_217_Hb3_HOL Vs Astrub_175_Hb2_AST : 23.12 % id

Apojap_217_Hb3_HOL Vs Cauare_159_HOL_P80018.3 : 39.62 % id

Apojap_217_Hb3_HOL Vs Cauare_159_HOL_P80017.3 : 37.74 % id

Apojap_217_Hb3_HOL Vs Hemcor_144_Hb1_AST : 29.58 % id

Apojap_217_Hb3_HOL Vs Hemcor_144_Hb2_AST : 32.39 % id

Apojap_217_Hb3_HOL Vs Labann_206_AST : 38.38 % id

Apojap_217_Hb3_HOL Vs Labann_263_AST : 23.49 % id

Apojap_217_Hb3_HOL Vs Ophisim_158_Hba_AST : 29.49 % id

Apojap_217_Hb3_HOL Vs Ophsim_144_Hbb_AST : 26.76 % id

Apojap_217_Hb3_HOL Vs Parliv_229_ECH_AM524155.1 : 40.79 % id

Apojap_217_Hb3_HOL Vs Parchi_158_HOL_P15161.2 : 39.24 % id

Apojap_217_Hb3_HOL Vs Patmin : 24.50 % id

Apojap_217_Hb3_HOL Vs Patmin : 27.66 % id

Apojap_217_Hb3_HOL Vs Pisoch_516_D1_AST : 21.56 % id

Apojap_217_Hb3_HOL Vs Pisoch_516_D2_AST : 24.32 % id

Apojap_217_Hb3_HOL Vs Remgou_206_AST : 38.38 % id

Apojap_217_Hb3_HOL Vs Psafra_138_CRI : 9.49 % id

Apojap_217_Hb3_HOL Vs Psicha_196_AST : 36.76 % id

Apojap_217_Hb3_HOL Vs Ptetes_D1_468_AST : 24.20 % id

Apojap_217_Hb3_HOL Vs Ptetes_D2_468_AST : 26.09 % id

Apojap_217_Hb3_HOL Vs Strpur_166_ECH_XP_003729167.1 : 41.82 % id

Apojap_217_Hb3_HOL Vs Allfra_167_ECH : 40.36 % id

Apojap_217_Hb3_HOL Vs Psedep_155_ECH : 40.26 % id

Apojap_217_Hb3_HOL Vs Strdro_167_ECH : 40.96 % id

Apojap_217_Hb3_HOL Vs Strint¬167_ECH : 42.77 % id

Apojap_217_Hb3_HOL Vs Strnud_146_ECH : 39.31 % id

Apojap_217_Hb3_HOL Vs Strpal_167_ECH : 35.54 % id

Apojap_217_Hb3_HOL Vs Strpur_416_D1_XP_003725467.1 : 26.75 % id

Apojap_217_Hb3_HOL Vs Strpur_416_D2_XP_003725467.1 : 26.17 % id

Apojap_217_Hb3_HOL Vs Strpur_2146_D1_XP_001199205.2 : 23.53 % id

Apojap_217_Hb3_HOL Vs Strpur_2146_D2_XP_001199205.2 : 29.94 % id

Apojap_217_Hb3_HOL Vs Strpur_2146_D3_XP_001199205.2 : 28.76 % id

Apojap_217_Hb3_HOL Vs Strpur : 27.74 % id

Apojap_217_Hb3_HOL Vs Strpur : 25.83 % id

Apojap_217_Hb3_HOL Vs Strpur_2146_D6_XP_001199205.2 : 26.49 % id

Apojap_217_Hb3_HOL Vs Strpur_2146_D7_XP_001199205.2 : 26.49 % id

Apojap_217_Hb3_HOL Vs Strpur_2146_D8_XP_001199205.2 : 27.15 % id

Apojap_217_Hb3_HOL Vs Strpur_2146_D9_XP_001199205.2 : 26.49 % id

Apojap_217_Hb3_HOL Vs Strpur_2146_D10_XP_001199205.2 : 26.67 % id

Apojap_217_Hb3_HOL Vs Strpur_2146_D11_XP_001199205.2 : 28.48 % id

Apojap_217_Hb3_HOL Vs Strpur_2146_D12_XP_001199205.2 : 28.00 % id

Apojap_217_Hb3_HOL Vs Strpur_2146_D13_XP_001199205.2 : 25.32 % id

Apojap_217_Hb3_HOL Vs Strpur_2146_D14_XP_001199205.2 : 25.83 % id

Apojap_217_Hb3_HOL Vs Strpur_267_Adgb_XP_001195639.2 : 8.81 % id

Apojap_217_Hb3_HOL Vs Xyljan_537_AST : 26.32 % id

Apojap_167_Hb4 Vs Astrub_174_Hb1_AST : 18.46 % id

Apojap_167_Hb4 Vs Astrub_175_Hb2_AST : 25.19 % id

Apojap_167_Hb4 Vs Cauare_159_HOL_P80018.3 : 42.75 % id

Apojap_167_Hb4 Vs Cauare_159_HOL_P80017.3 : 39.69 % id

Apojap_167_Hb4 Vs Hemcor_144_Hb1_AST : 28.00 % id

Apojap_167_Hb4 Vs Hemcor_144_Hb2_AST : 29.60 % id

Apojap_167_Hb4 Vs Labann_206_AST : 40.15 % id

Apojap_167_Hb4 Vs Labann_263_AST : 22.90 % id

Apojap_167_Hb4 Vs Ophisim_158_Hba_AST : 31.54 % id

Apojap_167_Hb4 Vs Ophsim_144_Hbb_AST : 27.20 % id

Apojap_167_Hb4 Vs Parliv_229_ECH_AM524155.1 : 41.48 % id

Apojap_167_Hb4 Vs Parchi_158_HOL_P15161.2 : 42.31 % id

Apojap_167_Hb4 Vs Patmin : 23.48 % id

Apojap_167_Hb4 Vs Patmin : 26.98 % id

Apojap_167_Hb4 Vs Pisoch_516_D1_AST : 20.95 % id

Apojap_167_Hb4 Vs Pisoch_516_D2_AST : 23.85 % id

Apojap_167_Hb4 Vs Remgou_206_AST : 40.15 % id

Apojap_167_Hb4 Vs Psafra_138_CRI : 8.59 % id

Apojap_167_Hb4 Vs Psicha_196_AST : 37.96 % id

Apojap_167_Hb4 Vs Ptetes_D1_468_AST : 24.46 % id

Apojap_167_Hb4 Vs Ptetes_D2_468_AST : 25.95 % id

Apojap_167_Hb4 Vs Strpur_166_ECH_XP_003729167.1 : 41.43 % id

Apojap_167_Hb4 Vs Allfra_167_ECH : 39.29 % id

Apojap_167_Hb4 Vs Psedep_155_ECH : 41.43 % id

Apojap_167_Hb4 Vs Strdro_167_ECH : 40.00 % id

Apojap_167_Hb4 Vs Strint¬167_ECH : 42.14 % id

Apojap_167_Hb4 Vs Strnud_146_ECH : 40.00 % id

Apojap_167_Hb4 Vs Strpal_167_ECH : 36.43 % id

Apojap_167_Hb4 Vs Strpur_416_D1_XP_003725467.1 : 25.90 % id

Apojap_167_Hb4 Vs Strpur_416_D2_XP_003725467.1 : 25.27 % id

Apojap_167_Hb4 Vs Strpur_2146_D1_XP_001199205.2 : 24.24 % id

Apojap_167_Hb4 Vs Strpur_2146_D2_XP_001199205.2 : 30.22 % id

Apojap_167_Hb4 Vs Strpur_2146_D3_XP_001199205.2 : 27.21 % id

Apojap_167_Hb4 Vs Strpur : 29.20 % id

Apojap_167_Hb4 Vs Strpur : 25.56 % id

Apojap_167_Hb4 Vs Strpur_2146_D6_XP_001199205.2 : 26.32 % id

Apojap_167_Hb4 Vs Strpur_2146_D7_XP_001199205.2 : 26.32 % id

Apojap_167_Hb4 Vs Strpur_2146_D8_XP_001199205.2 : 27.07 % id

Apojap_167_Hb4 Vs Strpur_2146_D9_XP_001199205.2 : 26.32 % id

Apojap_167_Hb4 Vs Strpur_2146_D10_XP_001199205.2 : 27.27 % id

Apojap_167_Hb4 Vs Strpur_2146_D11_XP_001199205.2 : 27.82 % id

Apojap_167_Hb4 Vs Strpur_2146_D12_XP_001199205.2 : 26.52 % id

Apojap_167_Hb4 Vs Strpur_2146_D13_XP_001199205.2 : 26.47 % id

Apojap_167_Hb4 Vs Strpur_2146_D14_XP_001199205.2 : 26.15 % id

Apojap_167_Hb4 Vs Strpur_267_Adgb_XP_001195639.2 : 9.66 % id

Apojap_167_Hb4 Vs Xyljan_537_AST : 25.37 % id

Astrub_174_Hb1_AST Vs Astrub_175_Hb2_AST : 27.75 % id

Astrub_174_Hb1_AST Vs Cauare_159_HOL_P80018.3 : 22.29 % id

Astrub_174_Hb1_AST Vs Cauare_159_HOL_P80017.3 : 15.92 % id

Astrub_174_Hb1_AST Vs Hemcor_144_Hb1_AST : 15.11 % id

Astrub_174_Hb1_AST Vs Hemcor_144_Hb2_AST : 17.99 % id

Astrub_174_Hb1_AST Vs Labann_206_AST : 20.23 % id

Astrub_174_Hb1_AST Vs Labann_263_AST : 31.54 % id

Astrub_174_Hb1_AST Vs Ophisim_158_Hba_AST : 17.11 % id

Astrub_174_Hb1_AST Vs Ophsim_144_Hbb_AST : 15.11 % id

Astrub_174_Hb1_AST Vs Parliv_229_ECH_AM524155.1 : 21.62 % id

Astrub_174_Hb1_AST Vs Parchi_158_HOL_P15161.2 : 21.02 % id

Astrub_174_Hb1_AST Vs Patmin : 34.67 % id

Astrub_174_Hb1_AST Vs Patmin : 57.04 % id

Astrub_174_Hb1_AST Vs Pisoch_516_D1_AST : 38.67 % id

Astrub_174_Hb1_AST Vs Pisoch_516_D2_AST : 30.41 % id

Astrub_174_Hb1_AST Vs Remgou_206_AST : 20.23 % id

Astrub_174_Hb1_AST Vs Psafra_138_CRI : 10.22 % id

Astrub_174_Hb1_AST Vs Psicha_196_AST : 18.50 % id

Astrub_174_Hb1_AST Vs Ptetes_D1_468_AST : 34.90 % id

Astrub_174_Hb1_AST Vs Ptetes_D2_468_AST : 30.43 % id

Astrub_174_Hb1_AST Vs Strpur_166_ECH_XP_003729167.1 : 19.87 % id

Astrub_174_Hb1_AST Vs Allfra_167_ECH : 19.11 % id

Astrub_174_Hb1_AST Vs Psedep_155_ECH : 20.69 % id

Astrub_174_Hb1_AST Vs Strdro_167_ECH : 19.75 % id

Astrub_174_Hb1_AST Vs Strint¬167_ECH : 19.75 % id

Astrub_174_Hb1_AST Vs Strnud_146_ECH : 19.85 % id

Astrub_174_Hb1_AST Vs Strpal_167_ECH : 18.47 % id

Astrub_174_Hb1_AST Vs Strpur_416_D1_XP_003725467.1 : 28.19 % id

Astrub_174_Hb1_AST Vs Strpur_416_D2_XP_003725467.1 : 32.41 % id

Astrub_174_Hb1_AST Vs Strpur_2146_D1_XP_001199205.2 : 26.32 % id

Astrub_174_Hb1_AST Vs Strpur_2146_D2_XP_001199205.2 : 31.54 % id

Astrub_174_Hb1_AST Vs Strpur_2146_D3_XP_001199205.2 : 25.68 % id

Astrub_174_Hb1_AST Vs Strpur : 32.21 % id

Astrub_174_Hb1_AST Vs Strpur : 31.54 % id

Astrub_174_Hb1_AST Vs Strpur_2146_D6_XP_001199205.2 : 32.89 % id

Astrub_174_Hb1_AST Vs Strpur_2146_D7_XP_001199205.2 : 32.89 % id

Astrub_174_Hb1_AST Vs Strpur_2146_D8_XP_001199205.2 : 33.56 % id

Astrub_174_Hb1_AST Vs Strpur_2146_D9_XP_001199205.2 : 34.23 % id

Astrub_174_Hb1_AST Vs Strpur_2146_D10_XP_001199205.2 : 29.53 % id

Astrub_174_Hb1_AST Vs Strpur_2146_D11_XP_001199205.2 : 30.20 % id

Astrub_174_Hb1_AST Vs Strpur_2146_D12_XP_001199205.2 : 26.85 % id

Astrub_174_Hb1_AST Vs Strpur_2146_D13_XP_001199205.2 : 30.87 % id

Astrub_174_Hb1_AST Vs Strpur_2146_D14_XP_001199205.2 : 25.00 % id

Astrub_174_Hb1_AST Vs Strpur_267_Adgb_XP_001195639.2 : 15.07 % id

Astrub_174_Hb1_AST Vs Xyljan_537_AST : 34.90 % id

Astrub_175_Hb2_AST Vs Cauare_159_HOL_P80018.3 : 20.25 % id

Astrub_175_Hb2_AST Vs Cauare_159_HOL_P80017.3 : 18.99 % id

Astrub_175_Hb2_AST Vs Hemcor_144_Hb1_AST : 21.43 % id

Astrub_175_Hb2_AST Vs Hemcor_144_Hb2_AST : 22.14 % id

Astrub_175_Hb2_AST Vs Labann_206_AST : 20.69 % id

Astrub_175_Hb2_AST Vs Labann_263_AST : 82.78 % id

Astrub_175_Hb2_AST Vs Ophisim_158_Hba_AST : 18.30 % id

Astrub_175_Hb2_AST Vs Ophsim_144_Hbb_AST : 20.00 % id

Astrub_175_Hb2_AST Vs Parliv_229_ECH_AM524155.1 : 26.17 % id

Astrub_175_Hb2_AST Vs Parchi_158_HOL_P15161.2 : 18.35 % id

Astrub_175_Hb2_AST Vs Patmin : 59.87 % id

Astrub_175_Hb2_AST Vs Patmin : 35.92 % id

Astrub_175_Hb2_AST Vs Pisoch_516_D1_AST : 55.92 % id

Astrub_175_Hb2_AST Vs Pisoch_516_D2_AST : 90.67 % id

Astrub_175_Hb2_AST Vs Remgou_206_AST : 20.69 % id

Astrub_175_Hb2_AST Vs Psafra_138_CRI : 13.77 % id

Astrub_175_Hb2_AST Vs Psicha_196_AST : 21.84 % id

Astrub_175_Hb2_AST Vs Ptetes_D1_468_AST : 54.97 % id

Astrub_175_Hb2_AST Vs Ptetes_D2_468_AST : 61.96 % id

Astrub_175_Hb2_AST Vs Strpur_166_ECH_XP_003729167.1 : 26.75 % id

Astrub_175_Hb2_AST Vs Allfra_167_ECH : 25.32 % id

Astrub_175_Hb2_AST Vs Psedep_155_ECH : 27.40 % id

Astrub_175_Hb2_AST Vs Strdro_167_ECH : 26.58 % id

Astrub_175_Hb2_AST Vs Strint¬167_ECH : 26.58 % id

Astrub_175_Hb2_AST Vs Strnud_146_ECH : 28.47 % id

Astrub_175_Hb2_AST Vs Strpal_167_ECH : 24.05 % id

Astrub_175_Hb2_AST Vs Strpur_416_D1_XP_003725467.1 : 40.00 % id

Astrub_175_Hb2_AST Vs Strpur_416_D2_XP_003725467.1 : 39.81 % id

Astrub_175_Hb2_AST Vs Strpur_2146_D1_XP_001199205.2 : 35.06 % id

Astrub_175_Hb2_AST Vs Strpur_2146_D2_XP_001199205.2 : 44.67 % id

Astrub_175_Hb2_AST Vs Strpur_2146_D3_XP_001199205.2 : 35.57 % id

Astrub_175_Hb2_AST Vs Strpur : 41.33 % id

Astrub_175_Hb2_AST Vs Strpur : 39.33 % id

Astrub_175_Hb2_AST Vs Strpur_2146_D6_XP_001199205.2 : 38.00 % id

Astrub_175_Hb2_AST Vs Strpur_2146_D7_XP_001199205.2 : 38.00 % id

Astrub_175_Hb2_AST Vs Strpur_2146_D8_XP_001199205.2 : 38.00 % id

Astrub_175_Hb2_AST Vs Strpur_2146_D9_XP_001199205.2 : 40.00 % id

Astrub_175_Hb2_AST Vs Strpur_2146_D10_XP_001199205.2 : 38.67 % id

Astrub_175_Hb2_AST Vs Strpur_2146_D11_XP_001199205.2 : 40.00 % id

Astrub_175_Hb2_AST Vs Strpur_2146_D12_XP_001199205.2 : 42.67 % id

Astrub_175_Hb2_AST Vs Strpur_2146_D13_XP_001199205.2 : 32.00 % id

Astrub_175_Hb2_AST Vs Strpur_2146_D14_XP_001199205.2 : 33.33 % id

Astrub_175_Hb2_AST Vs Strpur_267_Adgb_XP_001195639.2 : 19.18 % id

Astrub_175_Hb2_AST Vs Xyljan_537_AST : 54.97 % id

Cauare_159_HOL_P80018.3 Vs Cauare_159_HOL_P80017.3 : 60.38 % id

Cauare_159_HOL_P80018.3 Vs Hemcor_144_Hb1_AST : 26.43 % id

Cauare_159_HOL_P80018.3 Vs Hemcor_144_Hb2_AST : 27.86 % id

Cauare_159_HOL_P80018.3 Vs Labann_206_AST : 28.93 % id

Cauare_159_HOL_P80018.3 Vs Labann_263_AST : 19.59 % id

Cauare_159_HOL_P80018.3 Vs Ophisim_158_Hba_AST : 26.14 % id

Cauare_159_HOL_P80018.3 Vs Ophsim_144_Hbb_AST : 23.57 % id

Cauare_159_HOL_P80018.3 Vs Parliv_229_ECH_AM524155.1 : 32.43 % id

Cauare_159_HOL_P80018.3 Vs Parchi_158_HOL_P15161.2 : 93.04 % id

Cauare_159_HOL_P80018.3 Vs Patmin : 23.33 % id

Cauare_159_HOL_P80018.3 Vs Patmin : 18.57 % id

Cauare_159_HOL_P80018.3 Vs Pisoch_516_D1_AST : 22.67 % id

Cauare_159_HOL_P80018.3 Vs Pisoch_516_D2_AST : 21.77 % id

Cauare_159_HOL_P80018.3 Vs Remgou_206_AST : 28.93 % id

Cauare_159_HOL_P80018.3 Vs Psafra_138_CRI : 10.29 % id

Cauare_159_HOL_P80018.3 Vs Psicha_196_AST : 28.30 % id

Cauare_159_HOL_P80018.3 Vs Ptetes_D1_468_AST : 21.48 % id

Cauare_159_HOL_P80018.3 Vs Ptetes_D2_468_AST : 23.42 % id

Cauare_159_HOL_P80018.3 Vs Strpur_166_ECH_XP_003729167.1 : 28.21 % id

Cauare_159_HOL_P80018.3 Vs Allfra_167_ECH : 26.11 % id

Cauare_159_HOL_P80018.3 Vs Psedep_155_ECH : 28.28 % id

Cauare_159_HOL_P80018.3 Vs Strdro_167_ECH : 27.39 % id

Cauare_159_HOL_P80018.3 Vs Strint¬167_ECH : 28.03 % id

Cauare_159_HOL_P80018.3 Vs Strnud_146_ECH : 27.21 % id

Cauare_159_HOL_P80018.3 Vs Strpal_167_ECH : 25.48 % id

Cauare_159_HOL_P80018.3 Vs Strpur_416_D1_XP_003725467.1 : 22.15 % id

Cauare_159_HOL_P80018.3 Vs Strpur_416_D2_XP_003725467.1 : 29.25 % id

Cauare_159_HOL_P80018.3 Vs Strpur_2146_D1_XP_001199205.2 : 24.34 % id

Cauare_159_HOL_P80018.3 Vs Strpur_2146_D2_XP_001199205.2 : 26.85 % id

Cauare_159_HOL_P80018.3 Vs Strpur_2146_D3_XP_001199205.2 : 25.00 % id

Cauare_159_HOL_P80018.3 Vs Strpur : 29.53 % id

Cauare_159_HOL_P80018.3 Vs Strpur : 30.20 % id

Cauare_159_HOL_P80018.3 Vs Strpur_2146_D6_XP_001199205.2 : 29.53 % id

Cauare_159_HOL_P80018.3 Vs Strpur_2146_D7_XP_001199205.2 : 29.53 % id

Cauare_159_HOL_P80018.3 Vs Strpur_2146_D8_XP_001199205.2 : 30.20 % id

Cauare_159_HOL_P80018.3 Vs Strpur_2146_D9_XP_001199205.2 : 29.53 % id

Cauare_159_HOL_P80018.3 Vs Strpur_2146_D10_XP_001199205.2 : 24.83 % id

Cauare_159_HOL_P80018.3 Vs Strpur_2146_D11_XP_001199205.2 : 27.52 % id

Cauare_159_HOL_P80018.3 Vs Strpur_2146_D12_XP_001199205.2 : 24.83 % id

Cauare_159_HOL_P80018.3 Vs Strpur_2146_D13_XP_001199205.2 : 22.82 % id

Cauare_159_HOL_P80018.3 Vs Strpur_2146_D14_XP_001199205.2 : 20.67 % id

Cauare_159_HOL_P80018.3 Vs Strpur_267_Adgb_XP_001195639.2 : 11.03 % id

Cauare_159_HOL_P80018.3 Vs Xyljan_537_AST : 24.83 % id

Cauare_159_HOL_P80017.3 Vs Hemcor_144_Hb1_AST : 26.43 % id

Cauare_159_HOL_P80017.3 Vs Hemcor_144_Hb2_AST : 27.14 % id

Cauare_159_HOL_P80017.3 Vs Labann_206_AST : 28.30 % id

Cauare_159_HOL_P80017.3 Vs Labann_263_AST : 21.62 % id

Cauare_159_HOL_P80017.3 Vs Ophisim_158_Hba_AST : 22.88 % id

Cauare_159_HOL_P80017.3 Vs Ophsim_144_Hbb_AST : 21.43 % id

Cauare_159_HOL_P80017.3 Vs Parliv_229_ECH_AM524155.1 : 27.03 % id

Cauare_159_HOL_P80017.3 Vs Parchi_158_HOL_P15161.2 : 62.03 % id

Cauare_159_HOL_P80017.3 Vs Patmin : 22.67 % id

Cauare_159_HOL_P80017.3 Vs Patmin : 15.00 % id

Cauare_159_HOL_P80017.3 Vs Pisoch_516_D1_AST : 20.00 % id

Cauare_159_HOL_P80017.3 Vs Pisoch_516_D2_AST : 20.41 % id

Cauare_159_HOL_P80017.3 Vs Remgou_206_AST : 28.30 % id

Cauare_159_HOL_P80017.3 Vs Psafra_138_CRI : 9.56 % id

Cauare_159_HOL_P80017.3 Vs Psicha_196_AST : 25.79 % id

Cauare_159_HOL_P80017.3 Vs Ptetes_D1_468_AST : 20.81 % id

Cauare_159_HOL_P80017.3 Vs Ptetes_D2_468_AST : 21.52 % id

Cauare_159_HOL_P80017.3 Vs Strpur_166_ECH_XP_003729167.1 : 25.00 % id

Cauare_159_HOL_P80017.3 Vs Allfra_167_ECH : 22.93 % id

Cauare_159_HOL_P80017.3 Vs Psedep_155_ECH : 25.52 % id

Cauare_159_HOL_P80017.3 Vs Strdro_167_ECH : 24.84 % id

Cauare_159_HOL_P80017.3 Vs Strint¬167_ECH : 24.84 % id

Cauare_159_HOL_P80017.3 Vs Strnud_146_ECH : 25.00 % id

Cauare_159_HOL_P80017.3 Vs Strpal_167_ECH : 22.29 % id

Cauare_159_HOL_P80017.3 Vs Strpur_416_D1_XP_003725467.1 : 25.50 % id

Cauare_159_HOL_P80017.3 Vs Strpur_416_D2_XP_003725467.1 : 23.58 % id

Cauare_159_HOL_P80017.3 Vs Strpur_2146_D1_XP_001199205.2 : 24.34 % id

Cauare_159_HOL_P80017.3 Vs Strpur_2146_D2_XP_001199205.2 : 23.49 % id

Cauare_159_HOL_P80017.3 Vs Strpur_2146_D3_XP_001199205.2 : 23.65 % id

Cauare_159_HOL_P80017.3 Vs Strpur : 28.19 % id

Cauare_159_HOL_P80017.3 Vs Strpur : 28.19 % id

Cauare_159_HOL_P80017.3 Vs Strpur_2146_D6_XP_001199205.2 : 27.52 % id

Cauare_159_HOL_P80017.3 Vs Strpur_2146_D7_XP_001199205.2 : 27.52 % id

Cauare_159_HOL_P80017.3 Vs Strpur_2146_D8_XP_001199205.2 : 26.17 % id

Cauare_159_HOL_P80017.3 Vs Strpur_2146_D9_XP_001199205.2 : 28.19 % id

Cauare_159_HOL_P80017.3 Vs Strpur_2146_D10_XP_001199205.2 : 22.82 % id

Cauare_159_HOL_P80017.3 Vs Strpur_2146_D11_XP_001199205.2 : 30.20 % id

Cauare_159_HOL_P80017.3 Vs Strpur_2146_D12_XP_001199205.2 : 27.52 % id

Cauare_159_HOL_P80017.3 Vs Strpur_2146_D13_XP_001199205.2 : 23.49 % id

Cauare_159_HOL_P80017.3 Vs Strpur_2146_D14_XP_001199205.2 : 22.00 % id

Cauare_159_HOL_P80017.3 Vs Strpur_267_Adgb_XP_001195639.2 : 14.48 % id

Cauare_159_HOL_P80017.3 Vs Xyljan_537_AST : 22.82 % id

Hemcor_144_Hb1_AST Vs Hemcor_144_Hb2_AST : 89.58 % id

Hemcor_144_Hb1_AST Vs Labann_206_AST : 26.76 % id

Hemcor_144_Hb1_AST Vs Labann_263_AST : 20.00 % id

Hemcor_144_Hb1_AST Vs Ophisim_158_Hba_AST : 55.56 % id

Hemcor_144_Hb1_AST Vs Ophsim_144_Hbb_AST : 68.75 % id

Hemcor_144_Hb1_AST Vs Parliv_229_ECH_AM524155.1 : 25.35 % id

Hemcor_144_Hb1_AST Vs Parchi_158_HOL_P15161.2 : 25.18 % id

Hemcor_144_Hb1_AST Vs Patmin : 23.40 % id

Hemcor_144_Hb1_AST Vs Patmin : 18.05 % id

Hemcor_144_Hb1_AST Vs Pisoch_516_D1_AST : 21.83 % id

Hemcor_144_Hb1_AST Vs Pisoch_516_D2_AST : 20.14 % id

Hemcor_144_Hb1_AST Vs Remgou_206_AST : 26.76 % id

Hemcor_144_Hb1_AST Vs Psafra_138_CRI : 9.30 % id

Hemcor_144_Hb1_AST Vs Psicha_196_AST : 23.94 % id

Hemcor_144_Hb1_AST Vs Ptetes_D1_468_AST : 21.13 % id

Hemcor_144_Hb1_AST Vs Ptetes_D2_468_AST : 25.71 % id

Hemcor_144_Hb1_AST Vs Strpur_166_ECH_XP_003729167.1 : 26.76 % id

Hemcor_144_Hb1_AST Vs Allfra_167_ECH : 26.06 % id

Hemcor_144_Hb1_AST Vs Psedep_155_ECH : 27.34 % id

Hemcor_144_Hb1_AST Vs Strdro_167_ECH : 26.76 % id

Hemcor_144_Hb1_AST Vs Strint¬167_ECH : 26.76 % id

Hemcor_144_Hb1_AST Vs Strnud_146_ECH : 25.38 % id

Hemcor_144_Hb1_AST Vs Strpal_167_ECH : 22.54 % id

Hemcor_144_Hb1_AST Vs Strpur_416_D1_XP_003725467.1 : 21.13 % id

Hemcor_144_Hb1_AST Vs Strpur_416_D2_XP_003725467.1 : 29.52 % id

Hemcor_144_Hb1_AST Vs Strpur_2146_D1_XP_001199205.2 : 19.15 % id

Hemcor_144_Hb1_AST Vs Strpur_2146_D2_XP_001199205.2 : 22.54 % id

Hemcor_144_Hb1_AST Vs Strpur_2146_D3_XP_001199205.2 : 21.13 % id

Hemcor_144_Hb1_AST Vs Strpur : 19.72 % id

Hemcor_144_Hb1_AST Vs Strpur : 20.42 % id

Hemcor_144_Hb1_AST Vs Strpur_2146_D6_XP_001199205.2 : 20.42 % id

Hemcor_144_Hb1_AST Vs Strpur_2146_D7_XP_001199205.2 : 20.42 % id

Hemcor_144_Hb1_AST Vs Strpur_2146_D8_XP_001199205.2 : 19.72 % id

Hemcor_144_Hb1_AST Vs Strpur_2146_D9_XP_001199205.2 : 20.42 % id

Hemcor_144_Hb1_AST Vs Strpur_2146_D10_XP_001199205.2 : 22.70 % id

Hemcor_144_Hb1_AST Vs Strpur_2146_D11_XP_001199205.2 : 20.42 % id

Hemcor_144_Hb1_AST Vs Strpur_2146_D12_XP_001199205.2 : 19.86 % id

Hemcor_144_Hb1_AST Vs Strpur_2146_D13_XP_001199205.2 : 20.42 % id

Hemcor_144_Hb1_AST Vs Strpur_2146_D14_XP_001199205.2 : 22.30 % id

Hemcor_144_Hb1_AST Vs Strpur_267_Adgb_XP_001195639.2 : 11.51 % id

Hemcor_144_Hb1_AST Vs Xyljan_537_AST : 21.83 % id

Hemcor_144_Hb2_AST Vs Labann_206_AST : 26.76 % id

Hemcor_144_Hb2_AST Vs Labann_263_AST : 20.71 % id

Hemcor_144_Hb2_AST Vs Ophisim_158_Hba_AST : 59.72 % id

Hemcor_144_Hb2_AST Vs Ophsim_144_Hbb_AST : 66.67 % id

Hemcor_144_Hb2_AST Vs Parliv_229_ECH_AM524155.1 : 26.76 % id

Hemcor_144_Hb2_AST Vs Parchi_158_HOL_P15161.2 : 26.62 % id

Hemcor_144_Hb2_AST Vs Patmin : 25.53 % id

Hemcor_144_Hb2_AST Vs Patmin : 18.05 % id

Hemcor_144_Hb2_AST Vs Pisoch_516_D1_AST : 23.24 % id

Hemcor_144_Hb2_AST Vs Pisoch_516_D2_AST : 20.86 % id

Hemcor_144_Hb2_AST Vs Remgou_206_AST : 26.76 % id

Hemcor_144_Hb2_AST Vs Psafra_138_CRI : 10.85 % id

Hemcor_144_Hb2_AST Vs Psicha_196_AST : 25.35 % id

Hemcor_144_Hb2_AST Vs Ptetes_D1_468_AST : 22.54 % id

Hemcor_144_Hb2_AST Vs Ptetes_D2_468_AST : 27.14 % id

Hemcor_144_Hb2_AST Vs Strpur_166_ECH_XP_003729167.1 : 28.17 % id

Hemcor_144_Hb2_AST Vs Allfra_167_ECH : 27.46 % id

Hemcor_144_Hb2_AST Vs Psedep_155_ECH : 28.06 % id

Hemcor_144_Hb2_AST Vs Strdro_167_ECH : 28.17 % id

Hemcor_144_Hb2_AST Vs Strint¬167_ECH : 28.17 % id

Hemcor_144_Hb2_AST Vs Strnud_146_ECH : 26.15 % id

Hemcor_144_Hb2_AST Vs Strpal_167_ECH : 23.24 % id

Hemcor_144_Hb2_AST Vs Strpur_416_D1_XP_003725467.1 : 21.13 % id

Hemcor_144_Hb2_AST Vs Strpur_416_D2_XP_003725467.1 : 30.48 % id

Hemcor_144_Hb2_AST Vs Strpur_2146_D1_XP_001199205.2 : 19.86 % id

Hemcor_144_Hb2_AST Vs Strpur_2146_D2_XP_001199205.2 : 24.65 % id

Hemcor_144_Hb2_AST Vs Strpur_2146_D3_XP_001199205.2 : 23.24 % id

Hemcor_144_Hb2_AST Vs Strpur : 19.72 % id

Hemcor_144_Hb2_AST Vs Strpur : 20.42 % id

Hemcor_144_Hb2_AST Vs Strpur_2146_D6_XP_001199205.2 : 20.42 % id

Hemcor_144_Hb2_AST Vs Strpur_2146_D7_XP_001199205.2 : 20.42 % id

Hemcor_144_Hb2_AST Vs Strpur_2146_D8_XP_001199205.2 : 19.72 % id

Hemcor_144_Hb2_AST Vs Strpur_2146_D9_XP_001199205.2 : 21.83 % id

Hemcor_144_Hb2_AST Vs Strpur_2146_D10_XP_001199205.2 : 22.70 % id

Hemcor_144_Hb2_AST Vs Strpur_2146_D11_XP_001199205.2 : 21.83 % id

Hemcor_144_Hb2_AST Vs Strpur_2146_D12_XP_001199205.2 : 20.57 % id

Hemcor_144_Hb2_AST Vs Strpur_2146_D13_XP_001199205.2 : 19.72 % id

Hemcor_144_Hb2_AST Vs Strpur_2146_D14_XP_001199205.2 : 25.18 % id

Hemcor_144_Hb2_AST Vs Strpur_267_Adgb_XP_001195639.2 : 10.79 % id

Hemcor_144_Hb2_AST Vs Xyljan_537_AST : 23.94 % id

Labann_206_AST Vs Labann_263_AST : 21.33 % id

Labann_206_AST Vs Ophisim_158_Hba_AST : 28.21 % id

Labann_206_AST Vs Ophsim_144_Hbb_AST : 23.94 % id

Labann_206_AST Vs Parliv_229_ECH_AM524155.1 : 47.71 % id

Labann_206_AST Vs Parchi_158_HOL_P15161.2 : 27.22 % id

Labann_206_AST Vs Patmin : 25.00 % id

Labann_206_AST Vs Patmin : 23.94 % id

Labann_206_AST Vs Pisoch_516_D1_AST : 23.57 % id

Labann_206_AST Vs Pisoch_516_D2_AST : 22.15 % id

Labann_206_AST Vs Remgou_206_AST : 100.00 % id

Labann_206_AST Vs Psafra_138_CRI : 9.42 % id

Labann_206_AST Vs Psicha_196_AST : 72.16 % id

Labann_206_AST Vs Ptetes_D1_468_AST : 22.44 % id

Labann_206_AST Vs Ptetes_D2_468_AST : 25.31 % id

Labann_206_AST Vs Strpur_166_ECH_XP_003729167.1 : 49.69 % id

Labann_206_AST Vs Allfra_167_ECH : 48.78 % id

Labann_206_AST Vs Psedep_155_ECH : 48.68 % id

Labann_206_AST Vs Strdro_167_ECH : 48.78 % id

Labann_206_AST Vs Strint¬167_ECH : 50.00 % id

Labann_206_AST Vs Strnud_146_ECH : 45.45 % id

Labann_206_AST Vs Strpal_167_ECH : 43.29 % id

Labann_206_AST Vs Strpur_416_D1_XP_003725467.1 : 25.64 % id

Labann_206_AST Vs Strpur_416_D2_XP_003725467.1 : 22.22 % id

Labann_206_AST Vs Strpur_2146_D1_XP_001199205.2 : 23.38 % id

Labann_206_AST Vs Strpur_2146_D2_XP_001199205.2 : 26.28 % id

Labann_206_AST Vs Strpur_2146_D3_XP_001199205.2 : 25.32 % id

Labann_206_AST Vs Strpur : 22.44 % id

Labann_206_AST Vs Strpur : 25.00 % id

Labann_206_AST Vs Strpur_2146_D6_XP_001199205.2 : 23.68 % id

Labann_206_AST Vs Strpur_2146_D7_XP_001199205.2 : 23.68 % id

Labann_206_AST Vs Strpur_2146_D8_XP_001199205.2 : 25.00 % id

Labann_206_AST Vs Strpur_2146_D9_XP_001199205.2 : 26.97 % id

Labann_206_AST Vs Strpur_2146_D10_XP_001199205.2 : 19.21 % id

Labann_206_AST Vs Strpur_2146_D11_XP_001199205.2 : 25.00 % id

Labann_206_AST Vs Strpur_2146_D12_XP_001199205.2 : 27.81 % id

Labann_206_AST Vs Strpur_2146_D13_XP_001199205.2 : 23.23 % id

Labann_206_AST Vs Strpur_2146_D14_XP_001199205.2 : 25.00 % id

Labann_206_AST Vs Strpur_267_Adgb_XP_001195639.2 : 17.76 % id

Labann_206_AST Vs Xyljan_537_AST : 25.49 % id

Labann_263_AST Vs Ophisim_158_Hba_AST : 18.62 % id

Labann_263_AST Vs Ophsim_144_Hbb_AST : 19.29 % id

Labann_263_AST Vs Parliv_229_ECH_AM524155.1 : 22.82 % id

Labann_263_AST Vs Parchi_158_HOL_P15161.2 : 19.59 % id

Labann_263_AST Vs Patmin : 61.59 % id

Labann_263_AST Vs Patmin : 37.32 % id

Labann_263_AST Vs Pisoch_516_D1_AST : 59.60 % id

Labann_263_AST Vs Pisoch_516_D2_AST : 84.67 % id

Labann_263_AST Vs Remgou_206_AST : 21.33 % id

Labann_263_AST Vs Psafra_138_CRI : 10.87 % id

Labann_263_AST Vs Psicha_196_AST : 23.33 % id

Labann_263_AST Vs Ptetes_D1_468_AST : 56.29 % id

Labann_263_AST Vs Ptetes_D2_468_AST : 68.21 % id

Labann_263_AST Vs Strpur_166_ECH_XP_003729167.1 : 24.67 % id

Labann_263_AST Vs Allfra_167_ECH : 23.33 % id

Labann_263_AST Vs Psedep_155_ECH : 25.34 % id

Labann_263_AST Vs Strdro_167_ECH : 24.67 % id

Labann_263_AST Vs Strint¬167_ECH : 24.67 % id

Labann_263_AST Vs Strnud_146_ECH : 25.55 % id

Labann_263_AST Vs Strpal_167_ECH : 23.33 % id

Labann_263_AST Vs Strpur_416_D1_XP_003725467.1 : 38.00 % id

Labann_263_AST Vs Strpur_416_D2_XP_003725467.1 : 41.67 % id

Labann_263_AST Vs Strpur_2146_D1_XP_001199205.2 : 35.10 % id

Labann_263_AST Vs Strpur_2146_D2_XP_001199205.2 : 46.00 % id

Labann_263_AST Vs Strpur_2146_D3_XP_001199205.2 : 33.56 % id

Labann_263_AST Vs Strpur : 42.67 % id

Labann_263_AST Vs Strpur : 41.33 % id

Labann_263_AST Vs Strpur_2146_D6_XP_001199205.2 : 41.33 % id

Labann_263_AST Vs Strpur_2146_D7_XP_001199205.2 : 41.33 % id

Labann_263_AST Vs Strpur_2146_D8_XP_001199205.2 : 41.33 % id

Labann_263_AST Vs Strpur_2146_D9_XP_001199205.2 : 42.67 % id

Labann_263_AST Vs Strpur_2146_D10_XP_001199205.2 : 42.00 % id

Labann_263_AST Vs Strpur_2146_D11_XP_001199205.2 : 41.33 % id

Labann_263_AST Vs Strpur_2146_D12_XP_001199205.2 : 42.67 % id

Labann_263_AST Vs Strpur_2146_D13_XP_001199205.2 : 35.33 % id

Labann_263_AST Vs Strpur_2146_D14_XP_001199205.2 : 34.67 % id

Labann_263_AST Vs Strpur_267_Adgb_XP_001195639.2 : 19.18 % id

Labann_263_AST Vs Xyljan_537_AST : 56.29 % id

Ophisim_158_Hba_AST Vs Ophsim_144_Hbb_AST : 59.03 % id

Ophisim_158_Hba_AST Vs Parliv_229_ECH_AM524155.1 : 25.17 % id

Ophisim_158_Hba_AST Vs Parchi_158_HOL_P15161.2 : 25.00 % id

Ophisim_158_Hba_AST Vs Patmin : 20.41 % id

Ophisim_158_Hba_AST Vs Patmin : 18.25 % id

Ophisim_158_Hba_AST Vs Pisoch_516_D1_AST : 21.48 % id

Ophisim_158_Hba_AST Vs Pisoch_516_D2_AST : 18.75 % id

Ophisim_158_Hba_AST Vs Remgou_206_AST : 28.21 % id

Ophisim_158_Hba_AST Vs Psafra_138_CRI : 7.52 % id

Ophisim_158_Hba_AST Vs Psicha_196_AST : 30.13 % id

Ophisim_158_Hba_AST Vs Ptetes_D1_468_AST : 16.89 % id

Ophisim_158_Hba_AST Vs Ptetes_D2_468_AST : 20.26 % id

Ophisim_158_Hba_AST Vs Strpur_166_ECH_XP_003729167.1 : 25.16 % id

Ophisim_158_Hba_AST Vs Allfra_167_ECH : 24.36 % id

Ophisim_158_Hba_AST Vs Psedep_155_ECH : 27.08 % id

Ophisim_158_Hba_AST Vs Strdro_167_ECH : 25.00 % id

Ophisim_158_Hba_AST Vs Strint¬167_ECH : 25.00 % id

Ophisim_158_Hba_AST Vs Strnud_146_ECH : 25.19 % id

Ophisim_158_Hba_AST Vs Strpal_167_ECH : 21.15 % id

Ophisim_158_Hba_AST Vs Strpur_416_D1_XP_003725467.1 : 25.68 % id

Ophisim_158_Hba_AST Vs Strpur_416_D2_XP_003725467.1 : 26.67 % id

Ophisim_158_Hba_AST Vs Strpur_2146_D1_XP_001199205.2 : 21.48 % id

Ophisim_158_Hba_AST Vs Strpur_2146_D2_XP_001199205.2 : 21.62 % id

Ophisim_158_Hba_AST Vs Strpur_2146_D3_XP_001199205.2 : 20.41 % id

Ophisim_158_Hba_AST Vs Strpur : 25.00 % id

Ophisim_158_Hba_AST Vs Strpur : 23.81 % id

Ophisim_158_Hba_AST Vs Strpur_2146_D6_XP_001199205.2 : 23.81 % id

Ophisim_158_Hba_AST Vs Strpur_2146_D7_XP_001199205.2 : 23.81 % id

Ophisim_158_Hba_AST Vs Strpur_2146_D8_XP_001199205.2 : 23.13 % id

Ophisim_158_Hba_AST Vs Strpur_2146_D9_XP_001199205.2 : 23.81 % id

Ophisim_158_Hba_AST Vs Strpur_2146_D10_XP_001199205.2 : 22.60 % id

Ophisim_158_Hba_AST Vs Strpur_2146_D11_XP_001199205.2 : 22.45 % id

Ophisim_158_Hba_AST Vs Strpur_2146_D12_XP_001199205.2 : 21.92 % id

Ophisim_158_Hba_AST Vs Strpur_2146_D13_XP_001199205.2 : 22.97 % id

Ophisim_158_Hba_AST Vs Strpur_2146_D14_XP_001199205.2 : 26.53 % id

Ophisim_158_Hba_AST Vs Strpur_267_Adgb_XP_001195639.2 : 16.67 % id

Ophisim_158_Hba_AST Vs Xyljan_537_AST : 21.62 % id

Ophsim_144_Hbb_AST Vs Parliv_229_ECH_AM524155.1 : 23.94 % id

Ophsim_144_Hbb_AST Vs Parchi_158_HOL_P15161.2 : 22.30 % id

Ophsim_144_Hbb_AST Vs Patmin : 21.99 % id

Ophsim_144_Hbb_AST Vs Patmin : 18.80 % id

Ophsim_144_Hbb_AST Vs Pisoch_516_D1_AST : 21.83 % id

Ophsim_144_Hbb_AST Vs Pisoch_516_D2_AST : 20.14 % id

Ophsim_144_Hbb_AST Vs Remgou_206_AST : 23.94 % id

Ophsim_144_Hbb_AST Vs Psafra_138_CRI : 10.08 % id

Ophsim_144_Hbb_AST Vs Psicha_196_AST : 23.94 % id

Ophsim_144_Hbb_AST Vs Ptetes_D1_468_AST : 19.72 % id

Ophsim_144_Hbb_AST Vs Ptetes_D2_468_AST : 21.43 % id

Ophsim_144_Hbb_AST Vs Strpur_166_ECH_XP_003729167.1 : 25.35 % id

Ophsim_144_Hbb_AST Vs Allfra_167_ECH : 23.94 % id

Ophsim_144_Hbb_AST Vs Psedep_155_ECH : 25.90 % id

Ophsim_144_Hbb_AST Vs Strdro_167_ECH : 25.35 % id

Ophsim_144_Hbb_AST Vs Strint¬167_ECH : 25.35 % id

Ophsim_144_Hbb_AST Vs Strnud_146_ECH : 23.85 % id

Ophsim_144_Hbb_AST Vs Strpal_167_ECH : 22.54 % id

Ophsim_144_Hbb_AST Vs Strpur_416_D1_XP_003725467.1 : 19.72 % id

Ophsim_144_Hbb_AST Vs Strpur_416_D2_XP_003725467.1 : 28.57 % id

Ophsim_144_Hbb_AST Vs Strpur_2146_D1_XP_001199205.2 : 18.44 % id

Ophsim_144_Hbb_AST Vs Strpur_2146_D2_XP_001199205.2 : 21.13 % id

Ophsim_144_Hbb_AST Vs Strpur_2146_D3_XP_001199205.2 : 22.54 % id

Ophsim_144_Hbb_AST Vs Strpur : 19.72 % id

Ophsim_144_Hbb_AST Vs Strpur : 19.72 % id

Ophsim_144_Hbb_AST Vs Strpur_2146_D6_XP_001199205.2 : 19.01 % id

Ophsim_144_Hbb_AST Vs Strpur_2146_D7_XP_001199205.2 : 19.01 % id

Ophsim_144_Hbb_AST Vs Strpur_2146_D8_XP_001199205.2 : 19.01 % id

Ophsim_144_Hbb_AST Vs Strpur_2146_D9_XP_001199205.2 : 19.72 % id

Ophsim_144_Hbb_AST Vs Strpur_2146_D10_XP_001199205.2 : 20.57 % id

Ophsim_144_Hbb_AST Vs Strpur_2146_D11_XP_001199205.2 : 17.61 % id

Ophsim_144_Hbb_AST Vs Strpur_2146_D12_XP_001199205.2 : 19.15 % id

Ophsim_144_Hbb_AST Vs Strpur_2146_D13_XP_001199205.2 : 19.72 % id

Ophsim_144_Hbb_AST Vs Strpur_2146_D14_XP_001199205.2 : 23.02 % id

Ophsim_144_Hbb_AST Vs Strpur_267_Adgb_XP_001195639.2 : 13.67 % id

Ophsim_144_Hbb_AST Vs Xyljan_537_AST : 23.94 % id

Parliv_229_ECH_AM524155.1 Vs Parchi_158_HOL_P15161.2 : 32.65 % id

Parliv_229_ECH_AM524155.1 Vs Patmin : 26.67 % id

Parliv_229_ECH_AM524155.1 Vs Patmin : 19.01 % id

Parliv_229_ECH_AM524155.1 Vs Pisoch_516_D1_AST : 24.18 % id

Parliv_229_ECH_AM524155.1 Vs Pisoch_516_D2_AST : 24.32 % id

Parliv_229_ECH_AM524155.1 Vs Remgou_206_AST : 47.71 % id

Parliv_229_ECH_AM524155.1 Vs Psafra_138_CRI : 9.42 % id

Parliv_229_ECH_AM524155.1 Vs Psicha_196_AST : 49.02 % id

Parliv_229_ECH_AM524155.1 Vs Ptetes_D1_468_AST : 22.22 % id

Parliv_229_ECH_AM524155.1 Vs Ptetes_D2_468_AST : 25.50 % id

Parliv_229_ECH_AM524155.1 Vs Strpur_166_ECH_XP_003729167.1 : 83.01 % id

Parliv_229_ECH_AM524155.1 Vs Allfra_167_ECH : 79.74 % id

Parliv_229_ECH_AM524155.1 Vs Psedep_155_ECH : 80.67 % id

Parliv_229_ECH_AM524155.1 Vs Strdro_167_ECH : 81.70 % id

Parliv_229_ECH_AM524155.1 Vs Strint¬167_ECH : 82.35 % id

Parliv_229_ECH_AM524155.1 Vs Strnud_146_ECH : 79.43 % id

Parliv_229_ECH_AM524155.1 Vs Strpal_167_ECH : 75.16 % id

Parliv_229_ECH_AM524155.1 Vs Strpur_416_D1_XP_003725467.1 : 24.18 % id

Parliv_229_ECH_AM524155.1 Vs Strpur_416_D2_XP_003725467.1 : 27.78 % id

Parliv_229_ECH_AM524155.1 Vs Strpur_2146_D1_XP_001199205.2 : 26.00 % id

Parliv_229_ECH_AM524155.1 Vs Strpur_2146_D2_XP_001199205.2 : 26.80 % id

Parliv_229_ECH_AM524155.1 Vs Strpur_2146_D3_XP_001199205.2 : 32.03 % id

Parliv_229_ECH_AM524155.1 Vs Strpur : 25.49 % id

Parliv_229_ECH_AM524155.1 Vs Strpur : 23.84 % id

Parliv_229_ECH_AM524155.1 Vs Strpur_2146_D6_XP_001199205.2 : 24.50 % id

Parliv_229_ECH_AM524155.1 Vs Strpur_2146_D7_XP_001199205.2 : 24.50 % id

Parliv_229_ECH_AM524155.1 Vs Strpur_2146_D8_XP_001199205.2 : 25.83 % id

Parliv_229_ECH_AM524155.1 Vs Strpur_2146_D9_XP_001199205.2 : 29.14 % id

Parliv_229_ECH_AM524155.1 Vs Strpur_2146_D10_XP_001199205.2 : 22.00 % id

Parliv_229_ECH_AM524155.1 Vs Strpur_2146_D11_XP_001199205.2 : 21.85 % id

Parliv_229_ECH_AM524155.1 Vs Strpur_2146_D12_XP_001199205.2 : 29.33 % id

Parliv_229_ECH_AM524155.1 Vs Strpur_2146_D13_XP_001199205.2 : 20.26 % id

Parliv_229_ECH_AM524155.1 Vs Strpur_2146_D14_XP_001199205.2 : 23.65 % id

Parliv_229_ECH_AM524155.1 Vs Strpur_267_Adgb_XP_001195639.2 : 14.67 % id

Parliv_229_ECH_AM524155.1 Vs Xyljan_537_AST : 23.03 % id

Parchi_158_HOL_P15161.2 Vs Patmin : 24.16 % id

Parchi_158_HOL_P15161.2 Vs Patmin : 18.57 % id

Parchi_158_HOL_P15161.2 Vs Pisoch_516_D1_AST : 22.82 % id

Parchi_158_HOL_P15161.2 Vs Pisoch_516_D2_AST : 21.77 % id

Parchi_158_HOL_P15161.2 Vs Remgou_206_AST : 27.22 % id

Parchi_158_HOL_P15161.2 Vs Psafra_138_CRI : 10.29 % id

Parchi_158_HOL_P15161.2 Vs Psicha_196_AST : 27.22 % id

Parchi_158_HOL_P15161.2 Vs Ptetes_D1_468_AST : 20.27 % id

Parchi_158_HOL_P15161.2 Vs Ptetes_D2_468_AST : 21.52 % id

Parchi_158_HOL_P15161.2 Vs Strpur_166_ECH_XP_003729167.1 : 28.39 % id

Parchi_158_HOL_P15161.2 Vs Allfra_167_ECH : 26.28 % id

Parchi_158_HOL_P15161.2 Vs Psedep_155_ECH : 28.47 % id

Parchi_158_HOL_P15161.2 Vs Strdro_167_ECH : 27.56 % id

Parchi_158_HOL_P15161.2 Vs Strint¬167_ECH : 28.21 % id

Parchi_158_HOL_P15161.2 Vs Strnud_146_ECH : 27.41 % id

Parchi_158_HOL_P15161.2 Vs Strpal_167_ECH : 25.64 % id

Parchi_158_HOL_P15161.2 Vs Strpur_416_D1_XP_003725467.1 : 21.62 % id

Parchi_158_HOL_P15161.2 Vs Strpur_416_D2_XP_003725467.1 : 29.25 % id

Parchi_158_HOL_P15161.2 Vs Strpur_2146_D1_XP_001199205.2 : 23.84 % id

Parchi_158_HOL_P15161.2 Vs Strpur_2146_D2_XP_001199205.2 : 25.00 % id

Parchi_158_HOL_P15161.2 Vs Strpur_2146_D3_XP_001199205.2 : 25.17 % id

Parchi_158_HOL_P15161.2 Vs Strpur : 29.05 % id

Parchi_158_HOL_P15161.2 Vs Strpur : 28.38 % id

Parchi_158_HOL_P15161.2 Vs Strpur_2146_D6_XP_001199205.2 : 28.38 % id

Parchi_158_HOL_P15161.2 Vs Strpur_2146_D7_XP_001199205.2 : 28.38 % id

Parchi_158_HOL_P15161.2 Vs Strpur_2146_D8_XP_001199205.2 : 29.05 % id

Parchi_158_HOL_P15161.2 Vs Strpur_2146_D9_XP_001199205.2 : 28.38 % id

Parchi_158_HOL_P15161.2 Vs Strpur_2146_D10_XP_001199205.2 : 25.00 % id

Parchi_158_HOL_P15161.2 Vs Strpur_2146_D11_XP_001199205.2 : 25.68 % id

Parchi_158_HOL_P15161.2 Vs Strpur_2146_D12_XP_001199205.2 : 23.65 % id

Parchi_158_HOL_P15161.2 Vs Strpur_2146_D13_XP_001199205.2 : 23.65 % id

Parchi_158_HOL_P15161.2 Vs Strpur_2146_D14_XP_001199205.2 : 19.33 % id

Parchi_158_HOL_P15161.2 Vs Strpur_267_Adgb_XP_001195639.2 : 11.11 % id

Parchi_158_HOL_P15161.2 Vs Xyljan_537_AST : 23.65 % id

Patmin Vs Patmin : 31.69 % id

Patmin Vs Pisoch_516_D1_AST : 60.78 % id

Patmin Vs Pisoch_516_D2_AST : 59.33 % id

Patmin Vs Remgou_206_AST : 25.00 % id

Patmin Vs Psafra_138_CRI : 8.70 % id

Patmin Vs Psicha_196_AST : 27.63 % id

Patmin Vs Ptetes_D1_468_AST : 53.29 % id

Patmin Vs Ptetes_D2_468_AST : 55.92 % id

Patmin Vs Strpur_166_ECH_XP_003729167.1 : 27.63 % id

Patmin Vs Allfra_167_ECH : 26.32 % id

Patmin Vs Psedep_155_ECH : 27.89 % id

Patmin Vs Strdro_167_ECH : 27.63 % id

Patmin Vs Strint¬167_ECH : 27.63 % id

Patmin Vs Strnud_146_ECH : 28.26 % id

Patmin Vs Strpal_167_ECH : 25.66 % id

Patmin Vs Strpur_416_D1_XP_003725467.1 : 39.07 % id

Patmin Vs Strpur_416_D2_XP_003725467.1 : 44.44 % id

Patmin Vs Strpur_2146_D1_XP_001199205.2 : 35.95 % id

Patmin Vs Strpur_2146_D2_XP_001199205.2 : 44.37 % id

Patmin Vs Strpur_2146_D3_XP_001199205.2 : 38.67 % id

Patmin Vs Strpur : 39.07 % id

Patmin Vs Strpur : 38.41 % id

Patmin Vs Strpur_2146_D6_XP_001199205.2 : 37.75 % id

Patmin Vs Strpur_2146_D7_XP_001199205.2 : 37.75 % id

Patmin Vs Strpur_2146_D8_XP_001199205.2 : 38.41 % id

Patmin Vs Strpur_2146_D9_XP_001199205.2 : 41.06 % id

Patmin Vs Strpur_2146_D10_XP_001199205.2 : 37.75 % id

Patmin Vs Strpur_2146_D11_XP_001199205.2 : 37.75 % id

Patmin Vs Strpur_2146_D12_XP_001199205.2 : 44.37 % id

Patmin Vs Strpur_2146_D13_XP_001199205.2 : 34.44 % id

Patmin Vs Strpur_2146_D14_XP_001199205.2 : 35.10 % id

Patmin Vs Strpur_267_Adgb_XP_001195639.2 : 20.41 % id

Patmin Vs Xyljan_537_AST : 55.92 % id

Patmin Vs Pisoch_516_D1_AST : 35.21 % id

Patmin Vs Pisoch_516_D2_AST : 36.17 % id

Patmin Vs Remgou_206_AST : 23.94 % id

Patmin Vs Psafra_138_CRI : 12.03 % id

Patmin Vs Psicha_196_AST : 20.42 % id

Patmin Vs Ptetes_D1_468_AST : 29.58 % id

Patmin Vs Ptetes_D2_468_AST : 34.51 % id

Patmin Vs Strpur_166_ECH_XP_003729167.1 : 21.13 % id

Patmin Vs Allfra_167_ECH : 19.72 % id

Patmin Vs Psedep_155_ECH : 21.28 % id

Patmin Vs Strdro_167_ECH : 21.13 % id

Patmin Vs Strint¬167_ECH : 21.13 % id

Patmin Vs Strnud_146_ECH : 21.97 % id

Patmin Vs Strpal_167_ECH : 19.01 % id

Patmin Vs Strpur_416_D1_XP_003725467.1 : 30.28 % id

Patmin Vs Strpur_416_D2_XP_003725467.1 : 28.97 % id

Patmin Vs Strpur_2146_D1_XP_001199205.2 : 28.17 % id

Patmin Vs Strpur_2146_D2_XP_001199205.2 : 30.99 % id

Patmin Vs Strpur_2146_D3_XP_001199205.2 : 28.17 % id

Patmin Vs Strpur : 34.51 % id

Patmin Vs Strpur : 30.99 % id

Patmin Vs Strpur_2146_D6_XP_001199205.2 : 30.99 % id

Patmin Vs Strpur_2146_D7_XP_001199205.2 : 30.99 % id

Patmin Vs Strpur_2146_D8_XP_001199205.2 : 31.69 % id

Patmin Vs Strpur_2146_D9_XP_001199205.2 : 33.80 % id

Patmin Vs Strpur_2146_D10_XP_001199205.2 : 31.69 % id

Patmin Vs Strpur_2146_D11_XP_001199205.2 : 31.69 % id

Patmin Vs Strpur_2146_D12_XP_001199205.2 : 27.46 % id

Patmin Vs Strpur_2146_D13_XP_001199205.2 : 29.58 % id

Patmin Vs Strpur_2146_D14_XP_001199205.2 : 24.65 % id

Patmin Vs Strpur_267_Adgb_XP_001195639.2 : 12.06 % id

Patmin Vs Xyljan_537_AST : 34.51 % id

Pisoch_516_D1_AST Vs Pisoch_516_D2_AST : 54.67 % id

Pisoch_516_D1_AST Vs Remgou_206_AST : 23.57 % id

Pisoch_516_D1_AST Vs Psafra_138_CRI : 6.52 % id

Pisoch_516_D1_AST Vs Psicha_196_AST : 26.75 % id

Pisoch_516_D1_AST Vs Ptetes_D1_468_AST : 53.46 % id

Pisoch_516_D1_AST Vs Ptetes_D2_468_AST : 63.16 % id

Pisoch_516_D1_AST Vs Strpur_166_ECH_XP_003729167.1 : 22.50 % id

Pisoch_516_D1_AST Vs Allfra_167_ECH : 21.88 % id

Pisoch_516_D1_AST Vs Psedep_155_ECH : 22.58 % id

Pisoch_516_D1_AST Vs Strdro_167_ECH : 22.50 % id

Pisoch_516_D1_AST Vs Strint¬167_ECH : 22.50 % id

Pisoch_516_D1_AST Vs Strnud_146_ECH : 22.60 % id

Pisoch_516_D1_AST Vs Strpal_167_ECH : 19.38 % id

Pisoch_516_D1_AST Vs Strpur_416_D1_XP_003725467.1 : 37.97 % id

Pisoch_516_D1_AST Vs Strpur_416_D2_XP_003725467.1 : 44.44 % id

Pisoch_516_D1_AST Vs Strpur_2146_D1_XP_001199205.2 : 38.56 % id

Pisoch_516_D1_AST Vs Strpur_2146_D2_XP_001199205.2 : 41.77 % id

Pisoch_516_D1_AST Vs Strpur_2146_D3_XP_001199205.2 : 32.47 % id

Pisoch_516_D1_AST Vs Strpur : 38.46 % id

Pisoch_516_D1_AST Vs Strpur : 38.82 % id

Pisoch_516_D1_AST Vs Strpur_2146_D6_XP_001199205.2 : 40.13 % id

Pisoch_516_D1_AST Vs Strpur_2146_D7_XP_001199205.2 : 40.13 % id

Pisoch_516_D1_AST Vs Strpur_2146_D8_XP_001199205.2 : 40.79 % id

Pisoch_516_D1_AST Vs Strpur_2146_D9_XP_001199205.2 : 41.45 % id

Pisoch_516_D1_AST Vs Strpur_2146_D10_XP_001199205.2 : 37.09 % id

Pisoch_516_D1_AST Vs Strpur_2146_D11_XP_001199205.2 : 39.47 % id

Pisoch_516_D1_AST Vs Strpur_2146_D12_XP_001199205.2 : 41.06 % id

Pisoch_516_D1_AST Vs Strpur_2146_D13_XP_001199205.2 : 32.90 % id

Pisoch_516_D1_AST Vs Strpur_2146_D14_XP_001199205.2 : 32.45 % id

Pisoch_516_D1_AST Vs Strpur_267_Adgb_XP_001195639.2 : 15.62 % id

Pisoch_516_D1_AST Vs Xyljan_537_AST : 57.14 % id

Pisoch_516_D2_AST Vs Remgou_206_AST : 22.15 % id

Pisoch_516_D2_AST Vs Psafra_138_CRI : 13.14 % id

Pisoch_516_D2_AST Vs Psicha_196_AST : 23.49 % id

Pisoch_516_D2_AST Vs Ptetes_D1_468_AST : 55.33 % id

Pisoch_516_D2_AST Vs Ptetes_D2_468_AST : 66.00 % id

Pisoch_516_D2_AST Vs Strpur_166_ECH_XP_003729167.1 : 26.17 % id

Pisoch_516_D2_AST Vs Allfra_167_ECH : 24.83 % id

Pisoch_516_D2_AST Vs Psedep_155_ECH : 26.90 % id

Pisoch_516_D2_AST Vs Strdro_167_ECH : 26.17 % id

Pisoch_516_D2_AST Vs Strint¬167_ECH : 26.17 % id

Pisoch_516_D2_AST Vs Strnud_146_ECH : 27.94 % id

Pisoch_516_D2_AST Vs Strpal_167_ECH : 24.83 % id

Pisoch_516_D2_AST Vs Strpur_416_D1_XP_003725467.1 : 37.58 % id

Pisoch_516_D2_AST Vs Strpur_416_D2_XP_003725467.1 : 42.99 % id

Pisoch_516_D2_AST Vs Strpur_2146_D1_XP_001199205.2 : 35.33 % id

Pisoch_516_D2_AST Vs Strpur_2146_D2_XP_001199205.2 : 45.64 % id

Pisoch_516_D2_AST Vs Strpur_2146_D3_XP_001199205.2 : 33.11 % id

Pisoch_516_D2_AST Vs Strpur : 40.94 % id

Pisoch_516_D2_AST Vs Strpur : 40.27 % id

Pisoch_516_D2_AST Vs Strpur_2146_D6_XP_001199205.2 : 38.93 % id

Pisoch_516_D2_AST Vs Strpur_2146_D7_XP_001199205.2 : 38.93 % id

Pisoch_516_D2_AST Vs Strpur_2146_D8_XP_001199205.2 : 39.60 % id

Pisoch_516_D2_AST Vs Strpur_2146_D9_XP_001199205.2 : 40.27 % id

Pisoch_516_D2_AST Vs Strpur_2146_D10_XP_001199205.2 : 41.61 % id

Pisoch_516_D2_AST Vs Strpur_2146_D11_XP_001199205.2 : 41.61 % id

Pisoch_516_D2_AST Vs Strpur_2146_D12_XP_001199205.2 : 40.27 % id

Pisoch_516_D2_AST Vs Strpur_2146_D13_XP_001199205.2 : 32.21 % id

Pisoch_516_D2_AST Vs Strpur_2146_D14_XP_001199205.2 : 34.90 % id

Pisoch_516_D2_AST Vs Strpur_267_Adgb_XP_001195639.2 : 18.62 % id

Pisoch_516_D2_AST Vs Xyljan_537_AST : 54.67 % id

Remgou_206_AST Vs Psafra_138_CRI : 9.42 % id

Remgou_206_AST Vs Psicha_196_AST : 72.16 % id

Remgou_206_AST Vs Ptetes_D1_468_AST : 22.44 % id

Remgou_206_AST Vs Ptetes_D2_468_AST : 25.31 % id

Remgou_206_AST Vs Strpur_166_ECH_XP_003729167.1 : 49.69 % id

Remgou_206_AST Vs Allfra_167_ECH : 48.78 % id

Remgou_206_AST Vs Psedep_155_ECH : 48.68 % id

Remgou_206_AST Vs Strdro_167_ECH : 48.78 % id

Remgou_206_AST Vs Strint¬167_ECH : 50.00 % id

Remgou_206_AST Vs Strnud_146_ECH : 45.45 % id

Remgou_206_AST Vs Strpal_167_ECH : 43.29 % id

Remgou_206_AST Vs Strpur_416_D1_XP_003725467.1 : 25.64 % id

Remgou_206_AST Vs Strpur_416_D2_XP_003725467.1 : 22.22 % id

Remgou_206_AST Vs Strpur_2146_D1_XP_001199205.2 : 23.38 % id

Remgou_206_AST Vs Strpur_2146_D2_XP_001199205.2 : 26.28 % id

Remgou_206_AST Vs Strpur_2146_D3_XP_001199205.2 : 25.32 % id

Remgou_206_AST Vs Strpur : 22.44 % id

Remgou_206_AST Vs Strpur : 25.00 % id

Remgou_206_AST Vs Strpur_2146_D6_XP_001199205.2 : 23.68 % id

Remgou_206_AST Vs Strpur_2146_D7_XP_001199205.2 : 23.68 % id

Remgou_206_AST Vs Strpur_2146_D8_XP_001199205.2 : 25.00 % id

Remgou_206_AST Vs Strpur_2146_D9_XP_001199205.2 : 26.97 % id

Remgou_206_AST Vs Strpur_2146_D10_XP_001199205.2 : 19.21 % id

Remgou_206_AST Vs Strpur_2146_D11_XP_001199205.2 : 25.00 % id

Remgou_206_AST Vs Strpur_2146_D12_XP_001199205.2 : 27.81 % id

Remgou_206_AST Vs Strpur_2146_D13_XP_001199205.2 : 23.23 % id

Remgou_206_AST Vs Strpur_2146_D14_XP_001199205.2 : 25.00 % id

Remgou_206_AST Vs Strpur_267_Adgb_XP_001195639.2 : 17.76 % id

Remgou_206_AST Vs Xyljan_537_AST : 25.49 % id

Psafra_138_CRI Vs Psicha_196_AST : 7.97 % id

Psafra_138_CRI Vs Ptetes_D1_468_AST : 5.07 % id

Psafra_138_CRI Vs Ptetes_D2_468_AST : 7.25 % id

Psafra_138_CRI Vs Strpur_166_ECH_XP_003729167.1 : 10.14 % id

Psafra_138_CRI Vs Allfra_167_ECH : 10.14 % id

Psafra_138_CRI Vs Psedep_155_ECH : 10.87 % id

Psafra_138_CRI Vs Strdro_167_ECH : 10.14 % id

Psafra_138_CRI Vs Strint¬167_ECH : 10.14 % id

Psafra_138_CRI Vs Strnud_146_ECH : 9.70 % id

Psafra_138_CRI Vs Strpal_167_ECH : 10.87 % id

Psafra_138_CRI Vs Strpur_416_D1_XP_003725467.1 : 9.42 % id

Psafra_138_CRI Vs Strpur_416_D2_XP_003725467.1 : 11.22 % id

Psafra_138_CRI Vs Strpur_2146_D1_XP_001199205.2 : 6.52 % id

Psafra_138_CRI Vs Strpur_2146_D2_XP_001199205.2 : 8.70 % id

Psafra_138_CRI Vs Strpur_2146_D3_XP_001199205.2 : 9.42 % id

Psafra_138_CRI Vs Strpur_2146_D4_XP_001199205.2 : 8.70 % id

Psafra_138_CRI Vs Strpur_2146_D5_XP_001199205.2 : 10.87 % id

Psafra_138_CRI Vs Strpur_2146_D6_XP_001199205.2 : 9.42 % id

Psafra_138_CRI Vs Strpur_2146_D7_XP_001199205.2 : 9.42 % id

Psafra_138_CRI Vs Strpur_2146_D8_XP_001199205.2 : 9.42 % id

Psafra_138_CRI Vs Strpur_2146_D9_XP_001199205.2 : 6.52 % id

Psafra_138_CRI Vs Strpur_2146_D10_XP_001199205.2 : 12.32 % id

Psafra_138_CRI Vs Strpur_2146_D11_XP_001199205.2 : 11.59 % id

Psafra_138_CRI Vs Strpur_2146_D12_XP_001199205.2 : 11.59 % id

Psafra_138_CRI Vs Strpur_2146_D13_XP_001199205.2 : 8.70 % id

Psafra_138_CRI Vs Strpur_2146_D14_XP_001199205.2 : 8.03 % id

Psafra_138_CRI Vs Strpur_267_Adgb_XP_001195639.2 : 5.80 % id

Psafra_138_CRI Vs Xyljan_537_AST : 9.42 % id

Psicha_196_AST Vs Ptetes_D1_468_AST : 24.36 % id

Psicha_196_AST Vs Ptetes_D2_468_AST : 27.16 % id

Psicha_196_AST Vs Strpur_166_ECH_XP_003729167.1 : 50.31 % id

Psicha_196_AST Vs Allfra_167_ECH : 48.78 % id

Psicha_196_AST Vs Psedep_155_ECH : 48.03 % id

Psicha_196_AST Vs Strdro_167_ECH : 49.39 % id

Psicha_196_AST Vs Strint¬167_ECH : 50.61 % id

Psicha_196_AST Vs Strnud_146_ECH : 46.15 % id

Psicha_196_AST Vs Strpal_167_ECH : 43.90 % id

Psicha_196_AST Vs Strpur_416_D1_XP_003725467.1 : 26.92 % id

Psicha_196_AST Vs Strpur_416_D2_XP_003725467.1 : 23.15 % id

Psicha_196_AST Vs Strpur_2146_D1_XP_001199205.2 : 19.48 % id

Psicha_196_AST Vs Strpur_2146_D2_XP_001199205.2 : 23.72 % id

Psicha_196_AST Vs Strpur_2146_D3_XP_001199205.2 : 24.68 % id

Psicha_196_AST Vs Strpur : 21.79 % id

Psicha_196_AST Vs Strpur : 23.03 % id

Psicha_196_AST Vs Strpur_2146_D6_XP_001199205.2 : 23.03 % id

Psicha_196_AST Vs Strpur_2146_D7_XP_001199205.2 : 23.03 % id

Psicha_196_AST Vs Strpur_2146_D8_XP_001199205.2 : 24.34 % id

Psicha_196_AST Vs Strpur_2146_D9_XP_001199205.2 : 25.66 % id

Psicha_196_AST Vs Strpur_2146_D10_XP_001199205.2 : 21.85 % id

Psicha_196_AST Vs Strpur_2146_D11_XP_001199205.2 : 23.03 % id

Psicha_196_AST Vs Strpur_2146_D12_XP_001199205.2 : 25.17 % id

Psicha_196_AST Vs Strpur_2146_D13_XP_001199205.2 : 20.65 % id

Psicha_196_AST Vs Strpur_2146_D14_XP_001199205.2 : 23.68 % id

Psicha_196_AST Vs Strpur_267_Adgb_XP_001195639.2 : 16.88 % id

Psicha_196_AST Vs Xyljan_537_AST : 26.80 % id

Ptetes_D1_468_AST Vs Ptetes_D2_468_AST : 62.91 % id

Ptetes_D1_468_AST Vs Strpur_166_ECH_XP_003729167.1 : 20.89 % id

Ptetes_D1_468_AST Vs Allfra_167_ECH : 20.25 % id

Ptetes_D1_468_AST Vs Psedep_155_ECH : 21.43 % id

Ptetes_D1_468_AST Vs Strdro_167_ECH : 20.89 % id

Ptetes_D1_468_AST Vs Strint¬167_ECH : 20.89 % id

Ptetes_D1_468_AST Vs Strnud_146_ECH : 21.38 % id

Ptetes_D1_468_AST Vs Strpal_167_ECH : 19.62 % id

Ptetes_D1_468_AST Vs Strpur_416_D1_XP_003725467.1 : 34.18 % id

Ptetes_D1_468_AST Vs Strpur_416_D2_XP_003725467.1 : 39.81 % id

Ptetes_D1_468_AST Vs Strpur_2146_D1_XP_001199205.2 : 31.58 % id

Ptetes_D1_468_AST Vs Strpur_2146_D2_XP_001199205.2 : 38.61 % id

Ptetes_D1_468_AST Vs Strpur_2146_D3_XP_001199205.2 : 34.42 % id

Ptetes_D1_468_AST Vs Strpur : 39.74 % id

Ptetes_D1_468_AST Vs Strpur : 34.21 % id

Ptetes_D1_468_AST Vs Strpur_2146_D6_XP_001199205.2 : 34.87 % id

Ptetes_D1_468_AST Vs Strpur_2146_D7_XP_001199205.2 : 34.87 % id

Ptetes_D1_468_AST Vs Strpur_2146_D8_XP_001199205.2 : 33.55 % id

Ptetes_D1_468_AST Vs Strpur_2146_D9_XP_001199205.2 : 36.18 % id

Ptetes_D1_468_AST Vs Strpur_2146_D10_XP_001199205.2 : 33.77 % id

Ptetes_D1_468_AST Vs Strpur_2146_D11_XP_001199205.2 : 35.53 % id

Ptetes_D1_468_AST Vs Strpur_2146_D12_XP_001199205.2 : 38.41 % id

Ptetes_D1_468_AST Vs Strpur_2146_D13_XP_001199205.2 : 29.68 % id

Ptetes_D1_468_AST Vs Strpur_2146_D14_XP_001199205.2 : 29.33 % id

Ptetes_D1_468_AST Vs Strpur_267_Adgb_XP_001195639.2 : 15.58 % id

Ptetes_D1_468_AST Vs Xyljan_537_AST : 55.84 % id

Ptetes_D2_468_AST Vs Strpur_166_ECH_XP_003729167.1 : 26.11 % id

Ptetes_D2_468_AST Vs Allfra_167_ECH : 25.32 % id

Ptetes_D2_468_AST Vs Psedep_155_ECH : 26.03 % id

Ptetes_D2_468_AST Vs Strdro_167_ECH : 25.95 % id

Ptetes_D2_468_AST Vs Strint¬167_ECH : 25.95 % id

Ptetes_D2_468_AST Vs Strnud_146_ECH : 25.55 % id

Ptetes_D2_468_AST Vs Strpal_167_ECH : 22.15 % id

Ptetes_D2_468_AST Vs Strpur_416_D1_XP_003725467.1 : 36.67 % id

Ptetes_D2_468_AST Vs Strpur_416_D2_XP_003725467.1 : 41.67 % id

Ptetes_D2_468_AST Vs Strpur_2146_D1_XP_001199205.2 : 35.71 % id

Ptetes_D2_468_AST Vs Strpur_2146_D2_XP_001199205.2 : 42.67 % id

Ptetes_D2_468_AST Vs Strpur_2146_D3_XP_001199205.2 : 35.57 % id

Ptetes_D2_468_AST Vs Strpur : 44.00 % id

Ptetes_D2_468_AST Vs Strpur : 38.67 % id

Ptetes_D2_468_AST Vs Strpur_2146_D6_XP_001199205.2 : 41.33 % id

Ptetes_D2_468_AST Vs Strpur_2146_D7_XP_001199205.2 : 41.33 % id

Ptetes_D2_468_AST Vs Strpur_2146_D8_XP_001199205.2 : 40.67 % id

Ptetes_D2_468_AST Vs Strpur_2146_D9_XP_001199205.2 : 38.00 % id

Ptetes_D2_468_AST Vs Strpur_2146_D10_XP_001199205.2 : 36.67 % id

Ptetes_D2_468_AST Vs Strpur_2146_D11_XP_001199205.2 : 41.33 % id

Ptetes_D2_468_AST Vs Strpur_2146_D12_XP_001199205.2 : 40.00 % id

Ptetes_D2_468_AST Vs Strpur_2146_D13_XP_001199205.2 : 31.33 % id

Ptetes_D2_468_AST Vs Strpur_2146_D14_XP_001199205.2 : 33.33 % id

Ptetes_D2_468_AST Vs Strpur_267_Adgb_XP_001195639.2 : 17.81 % id

Ptetes_D2_468_AST Vs Xyljan_537_AST : 56.95 % id

Strpur_166_ECH_XP_003729167.1 Vs Allfra_167_ECH : 96.39 % id

Strpur_166_ECH_XP_003729167.1 Vs Psedep_155_ECH : 97.42 % id

Strpur_166_ECH_XP_003729167.1 Vs Strdro_167_ECH : 97.59 % id

Strpur_166_ECH_XP_003729167.1 Vs Strint¬167_ECH : 99.40 % id

Strpur_166_ECH_XP_003729167.1 Vs Strnud_146_ECH : 93.84 % id

Strpur_166_ECH_XP_003729167.1 Vs Strpal_167_ECH : 89.16 % id

Strpur_166_ECH_XP_003729167.1 Vs Strpur_416_D1_XP_003725467.1 : 24.68 % id

Strpur_166_ECH_XP_003729167.1 Vs Strpur_416_D2_XP_003725467.1 : 28.70 % id

Strpur_166_ECH_XP_003729167.1 Vs Strpur_2146_D1_XP_001199205.2 : 23.38 % id

Strpur_166_ECH_XP_003729167.1 Vs Strpur_2146_D2_XP_001199205.2 : 24.68 % id

Strpur_166_ECH_XP_003729167.1 Vs Strpur_2146_D3_XP_001199205.2 : 30.52 % id

Strpur_166_ECH_XP_003729167.1 Vs Strpur : 24.36 % id

Strpur_166_ECH_XP_003729167.1 Vs Strpur : 23.68 % id

Strpur_166_ECH_XP_003729167.1 Vs Strpur_2146_D6_XP_001199205.2 : 24.34 % id

Strpur_166_ECH_XP_003729167.1 Vs Strpur_2146_D7_XP_001199205.2 : 24.34 % id

Strpur_166_ECH_XP_003729167.1 Vs Strpur_2146_D8_XP_001199205.2 : 25.66 % id

Strpur_166_ECH_XP_003729167.1 Vs Strpur_2146_D9_XP_001199205.2 : 27.63 % id

Strpur_166_ECH_XP_003729167.1 Vs Strpur_2146_D10_XP_001199205.2 : 20.53 % id

Strpur_166_ECH_XP_003729167.1 Vs Strpur_2146_D11_XP_001199205.2 : 22.37 % id

Strpur_166_ECH_XP_003729167.1 Vs Strpur_2146_D12_XP_001199205.2 : 29.80 % id

Strpur_166_ECH_XP_003729167.1 Vs Strpur_2146_D13_XP_001199205.2 : 19.35 % id

Strpur_166_ECH_XP_003729167.1 Vs Strpur_2146_D14_XP_001199205.2 : 23.03 % id

Strpur_166_ECH_XP_003729167.1 Vs Strpur_267_Adgb_XP_001195639.2 : 12.90 % id

Strpur_166_ECH_XP_003729167.1 Vs Xyljan_537_AST : 24.18 % id

Allfra_167_ECH Vs Psedep_155_ECH : 95.48 % id

Allfra_167_ECH Vs Strdro_167_ECH : 94.01 % id

Allfra_167_ECH Vs Strint¬167_ECH : 95.81 % id

Allfra_167_ECH Vs Strnud_146_ECH : 92.47 % id

Allfra_167_ECH Vs Strpal_167_ECH : 87.43 % id

Allfra_167_ECH Vs Strpur_416_D1_XP_003725467.1 : 24.05 % id

Allfra_167_ECH Vs Strpur_416_D2_XP_003725467.1 : 26.85 % id

Allfra_167_ECH Vs Strpur_2146_D1_XP_001199205.2 : 21.43 % id

Allfra_167_ECH Vs Strpur_2146_D2_XP_001199205.2 : 22.78 % id

Allfra_167_ECH Vs Strpur_2146_D3_XP_001199205.2 : 29.22 % id

Allfra_167_ECH Vs Strpur_2146_D4_XP_001199205.2 : 22.44 % id

Allfra_167_ECH Vs Strpur_2146_D5_XP_001199205.2 : 21.71 % id

Allfra_167_ECH Vs Strpur_2146_D6_XP_001199205.2 : 22.37 % id

Allfra_167_ECH Vs Strpur_2146_D7_XP_001199205.2 : 22.37 % id

Allfra_167_ECH Vs Strpur_2146_D8_XP_001199205.2 : 23.68 % id

Allfra_167_ECH Vs Strpur_2146_D9_XP_001199205.2 : 26.32 % id

Allfra_167_ECH Vs Strpur_2146_D10_XP_001199205.2 : 19.21 % id

Allfra_167_ECH Vs Strpur_2146_D11_XP_001199205.2 : 20.39 % id

Allfra_167_ECH Vs Strpur_2146_D12_XP_001199205.2 : 27.81 % id

Allfra_167_ECH Vs Strpur_2146_D13_XP_001199205.2 : 17.42 % id

Allfra_167_ECH Vs Strpur_2146_D14_XP_001199205.2 : 21.71 % id

Allfra_167_ECH Vs Strpur_267_Adgb_XP_001195639.2 : 12.26 % id

Allfra_167_ECH Vs Xyljan_537_AST : 23.53 % id

Psedep_155_ECH Vs Strdro_167_ECH : 94.84 % id

Psedep_155_ECH Vs Strint¬167_ECH : 96.77 % id

Psedep_155_ECH Vs Strnud_146_ECH : 95.21 % id

Psedep_155_ECH Vs Strpal_167_ECH : 90.97 % id

Psedep_155_ECH Vs Strpur_416_D1_XP_003725467.1 : 24.03 % id

Psedep_155_ECH Vs Strpur_416_D2_XP_003725467.1 : 28.30 % id

Psedep_155_ECH Vs Strpur_2146_D1_XP_001199205.2 : 23.81 % id

Psedep_155_ECH Vs Strpur_2146_D2_XP_001199205.2 : 24.68 % id

Psedep_155_ECH Vs Strpur_2146_D3_XP_001199205.2 : 28.48 % id

Psedep_155_ECH Vs Strpur_2146_D4_XP_001199205.2 : 23.68 % id

Psedep_155_ECH Vs Strpur_2146_D5_XP_001199205.2 : 23.65 % id

Psedep_155_ECH Vs Strpur_2146_D6_XP_001199205.2 : 24.32 % id

Psedep_155_ECH Vs Strpur_2146_D7_XP_001199205.2 : 24.32 % id

Psedep_155_ECH Vs Strpur_2146_D8_XP_001199205.2 : 25.68 % id

Psedep_155_ECH Vs Strpur_2146_D9_XP_001199205.2 : 26.35 % id

Psedep_155_ECH Vs Strpur_2146_D10_XP_001199205.2 : 20.41 % id

Psedep_155_ECH Vs Strpur_2146_D11_XP_001199205.2 : 22.30 % id

Psedep_155_ECH Vs Strpur_2146_D12_XP_001199205.2 : 27.89 % id

Psedep_155_ECH Vs Strpur_2146_D13_XP_001199205.2 : 19.87 % id

Psedep_155_ECH Vs Strpur_2146_D14_XP_001199205.2 : 22.76 % id

Psedep_155_ECH Vs Strpur_267_Adgb_XP_001195639.2 : 12.90 % id

Psedep_155_ECH Vs Xyljan_537_AST : 24.83 % id

Strdro_167_ECH Vs Strint¬167_ECH : 97.60 % id

Strdro_167_ECH Vs Strnud_146_ECH : 92.47 % id

Strdro_167_ECH Vs Strpal_167_ECH : 88.02 % id

Strdro_167_ECH Vs Strpur_416_D1_XP_003725467.1 : 24.05 % id

Strdro_167_ECH Vs Strpur_416_D2_XP_003725467.1 : 28.70 % id

Strdro_167_ECH Vs Strpur_2146_D1_XP_001199205.2 : 23.38 % id

Strdro_167_ECH Vs Strpur_2146_D2_XP_001199205.2 : 24.68 % id

Strdro_167_ECH Vs Strpur_2146_D3_XP_001199205.2 : 29.87 % id

Strdro_167_ECH Vs Strpur_2146_D4_XP_001199205.2 : 23.72 % id

Strdro_167_ECH Vs Strpur_2146_D5_XP_001199205.2 : 23.68 % id

Strdro_167_ECH Vs Strpur_2146_D6_XP_001199205.2 : 24.34 % id

Strdro_167_ECH Vs Strpur_2146_D7_XP_001199205.2 : 24.34 % id

Strdro_167_ECH Vs Strpur_2146_D8_XP_001199205.2 : 25.66 % id

Strdro_167_ECH Vs Strpur_2146_D9_XP_001199205.2 : 27.63 % id

Strdro_167_ECH Vs Strpur_2146_D10_XP_001199205.2 : 19.87 % id

Strdro_167_ECH Vs Strpur_2146_D11_XP_001199205.2 : 22.37 % id

Strdro_167_ECH Vs Strpur_2146_D12_XP_001199205.2 : 29.80 % id

Strdro_167_ECH Vs Strpur_2146_D13_XP_001199205.2 : 19.35 % id

Strdro_167_ECH Vs Strpur_2146_D14_XP_001199205.2 : 23.03 % id

Strdro_167_ECH Vs Strpur_267_Adgb_XP_001195639.2 : 12.90 % id

Strdro_167_ECH Vs Xyljan_537_AST : 24.18 % id

Strint¬167_ECH Vs Strnud_146_ECH : 93.15 % id

Strint¬167_ECH Vs Strpal_167_ECH : 88.62 % id

Strint¬167_ECH Vs Strpur_416_D1_XP_003725467.1 : 24.68 % id

Strint¬167_ECH Vs Strpur_416_D2_XP_003725467.1 : 28.70 % id

Strint¬167_ECH Vs Strpur_2146_D1_XP_001199205.2 : 23.38 % id

Strint¬167_ECH Vs Strpur_2146_D2_XP_001199205.2 : 24.68 % id

Strint¬167_ECH Vs Strpur_2146_D3_XP_001199205.2 : 30.52 % id

Strint¬167_ECH Vs Strpur_2146_D4_XP_001199205.2 : 24.36 % id

Strint¬167_ECH Vs Strpur_2146_D5_XP_001199205.2 : 23.68 % id

Strint¬167_ECH Vs Strpur_2146_D6_XP_001199205.2 : 24.34 % id

Strint¬167_ECH Vs Strpur_2146_D7_XP_001199205.2 : 24.34 % id

Strint¬167_ECH Vs Strpur_2146_D8_XP_001199205.2 : 25.66 % id

Strint¬167_ECH Vs Strpur_2146_D9_XP_001199205.2 : 27.63 % id

Strint¬167_ECH Vs Strpur_2146_D10_XP_001199205.2 : 20.53 % id

Strint¬167_ECH Vs Strpur_2146_D11_XP_001199205.2 : 22.37 % id

Strint¬167_ECH Vs Strpur_2146_D12_XP_001199205.2 : 29.80 % id

Strint¬167_ECH Vs Strpur_2146_D13_XP_001199205.2 : 19.35 % id

Strint¬167_ECH Vs Strpur_2146_D14_XP_001199205.2 : 23.03 % id

Strint¬167_ECH Vs Strpur_267_Adgb_XP_001195639.2 : 12.90 % id

Strint¬167_ECH Vs Xyljan_537_AST : 24.18 % id

Strnud_146_ECH Vs Strpal_167_ECH : 86.99 % id

Strnud_146_ECH Vs Strpur_416_D1_XP_003725467.1 : 23.45 % id

Strnud_146_ECH Vs Strpur_416_D2_XP_003725467.1 : 27.84 % id

Strnud_146_ECH Vs Strpur_2146_D1_XP_001199205.2 : 21.74 % id

Strnud_146_ECH Vs Strpur_2146_D2_XP_001199205.2 : 22.76 % id

Strnud_146_ECH Vs Strpur_2146_D3_XP_001199205.2 : 26.06 % id

Strnud_146_ECH Vs Strpur_2146_D4_XP_001199205.2 : 23.08 % id

Strnud_146_ECH Vs Strpur_2146_D5_XP_001199205.2 : 21.58 % id

Strnud_146_ECH Vs Strpur_2146_D6_XP_001199205.2 : 22.30 % id

Strnud_146_ECH Vs Strpur_2146_D7_XP_001199205.2 : 22.30 % id

Strnud_146_ECH Vs Strpur_2146_D8_XP_001199205.2 : 23.02 % id

Strnud_146_ECH Vs Strpur_2146_D9_XP_001199205.2 : 25.90 % id

Strnud_146_ECH Vs Strpur_2146_D10_XP_001199205.2 : 18.12 % id

Strnud_146_ECH Vs Strpur_2146_D11_XP_001199205.2 : 22.30 % id

Strnud_146_ECH Vs Strpur_2146_D12_XP_001199205.2 : 26.09 % id

Strnud_146_ECH Vs Strpur_2146_D13_XP_001199205.2 : 19.01 % id

Strnud_146_ECH Vs Strpur_2146_D14_XP_001199205.2 : 22.79 % id

Strnud_146_ECH Vs Strpur_267_Adgb_XP_001195639.2 : 14.38 % id

Strnud_146_ECH Vs Xyljan_537_AST : 24.29 % id

Strpal_167_ECH Vs Strpur_416_D1_XP_003725467.1 : 21.52 % id

Strpal_167_ECH Vs Strpur_416_D2_XP_003725467.1 : 24.07 % id

Strpal_167_ECH Vs Strpur_2146_D1_XP_001199205.2 : 20.78 % id

Strpal_167_ECH Vs Strpur_2146_D2_XP_001199205.2 : 23.42 % id

Strpal_167_ECH Vs Strpur_2146_D3_XP_001199205.2 : 27.27 % id

Strpal_167_ECH Vs Strpur_2146_D4_XP_001199205.2 : 22.44 % id

Strpal_167_ECH Vs Strpur_2146_D5_XP_001199205.2 : 21.05 % id

Strpal_167_ECH Vs Strpur_2146_D6_XP_001199205.2 : 22.37 % id

Strpal_167_ECH Vs Strpur_2146_D7_XP_001199205.2 : 22.37 % id

Strpal_167_ECH Vs Strpur_2146_D8_XP_001199205.2 : 23.03 % id

Strpal_167_ECH Vs Strpur_2146_D9_XP_001199205.2 : 25.66 % id

Strpal_167_ECH Vs Strpur_2146_D10_XP_001199205.2 : 18.54 % id

Strpal_167_ECH Vs Strpur_2146_D11_XP_001199205.2 : 19.74 % id

Strpal_167_ECH Vs Strpur_2146_D12_XP_001199205.2 : 27.81 % id

Strpal_167_ECH Vs Strpur_2146_D13_XP_001199205.2 : 18.71 % id

Strpal_167_ECH Vs Strpur_2146_D14_XP_001199205.2 : 21.05 % id

Strpal_167_ECH Vs Strpur_267_Adgb_XP_001195639.2 : 12.26 % id

Strpal_167_ECH Vs Xyljan_537_AST : 21.57 % id

Strpur_416_D1_XP_003725467.1 Vs Strpur_416_D2_XP_003725467.1 : 43.52 % id

Strpur_416_D1_XP_003725467.1 Vs Strpur_2146_D1_XP_001199205.2 : 42.38 % id

Strpur_416_D1_XP_003725467.1 Vs Strpur_2146_D2_XP_001199205.2 : 48.73 % id

Strpur_416_D1_XP_003725467.1 Vs Strpur_2146_D3_XP_001199205.2 : 39.61 % id

Strpur_416_D1_XP_003725467.1 Vs Strpur_2146_D4_XP_001199205.2 : 46.79 % id

Strpur_416_D1_XP_003725467.1 Vs Strpur_2146_D5_XP_001199205.2 : 47.37 % id

Strpur_416_D1_XP_003725467.1 Vs Strpur_2146_D6_XP_001199205.2 : 47.37 % id

Strpur_416_D1_XP_003725467.1 Vs Strpur_2146_D7_XP_001199205.2 : 47.37 % id

Strpur_416_D1_XP_003725467.1 Vs Strpur_2146_D8_XP_001199205.2 : 48.68 % id

Strpur_416_D1_XP_003725467.1 Vs Strpur_2146_D9_XP_001199205.2 : 47.37 % id

Strpur_416_D1_XP_003725467.1 Vs Strpur_2146_D10_XP_001199205.2 : 39.74 % id

Strpur_416_D1_XP_003725467.1 Vs Strpur_2146_D11_XP_001199205.2 : 50.00 % id

Strpur_416_D1_XP_003725467.1 Vs Strpur_2146_D12_XP_001199205.2 : 43.71 % id

Strpur_416_D1_XP_003725467.1 Vs Strpur_2146_D13_XP_001199205.2 : 41.29 % id

Strpur_416_D1_XP_003725467.1 Vs Strpur_2146_D14_XP_001199205.2 : 41.61 % id

Strpur_416_D1_XP_003725467.1 Vs Strpur_267_Adgb_XP_001195639.2 : 20.13 % id

Strpur_416_D1_XP_003725467.1 Vs Xyljan_537_AST : 35.95 % id

Strpur_416_D2_XP_003725467.1 Vs Strpur_2146_D1_XP_001199205.2 : 47.22 % id

Strpur_416_D2_XP_003725467.1 Vs Strpur_2146_D2_XP_001199205.2 : 52.78 % id

Strpur_416_D2_XP_003725467.1 Vs Strpur_2146_D5_XP_001199205.2 : 40.74 % id

Strpur_416_D2_XP_003725467.1 Vs Strpur : 52.78 % id

Strpur_416_D2_XP_003725467.1 Vs Strpur_2146_D4_XP_001199205.2 : 57.41 % id

Strpur_416_D2_XP_003725467.1 Vs Strpur_2146_D6_XP_001199205.2 : 57.41 % id

Strpur_416_D2_XP_003725467.1 Vs Strpur_2146_D7_XP_001199205.2 : 57.41 % id

Strpur_416_D2_XP_003725467.1 Vs Strpur_2146_D8_XP_001199205.2 : 57.41 % id

Strpur_416_D2_XP_003725467.1 Vs Strpur_2146_D9_XP_001199205.2 : 53.70 % id

Strpur_416_D2_XP_003725467.1 Vs Strpur_2146_D10_XP_001199205.2 : 50.00 % id

Strpur_416_D2_XP_003725467.1 Vs Strpur_2146_D11_XP_001199205.2 : 52.78 % id

Strpur_416_D2_XP_003725467.1 Vs Strpur_2146_D12_XP_001199205.2 : 49.07 % id

Strpur_416_D2_XP_003725467.1 Vs Strpur_2146_D13_XP_001199205.2 : 44.44 % id

Strpur_416_D2_XP_003725467.1 Vs Strpur_2146_D14_XP_001199205.2 : 41.67 % id

Strpur_416_D2_XP_003725467.1 Vs Strpur_267_Adgb_XP_001195639.2 : 16.98 % id

Strpur_416_D2_XP_003725467.1 Vs Xyljan_537_AST : 44.44 % id

Strpur_2146_D1_XP_001199205.2 Vs Strpur_2146_D2_XP_001199205.2 : 50.33 % id

Strpur_2146_D1_XP_001199205.2 Vs Strpur_2146_D3_XP_001199205.2 : 39.33 % id

Strpur_2146_D1_XP_001199205.2 Vs Strpur_2146_D4_XP_001199205.2 : 50.33 % id

Strpur_2146_D1_XP_001199205.2 Vs Strpur_2146_D5_XP_001199205.2 : 52.98 % id

Strpur_2146_D1_XP_001199205.2 Vs Strpur_2146_D6_XP_001199205.2 : 49.67 % id

Strpur_2146_D1_XP_001199205.2 Vs Strpur_2146_D7_XP_001199205.2 : 49.67 % id

Strpur_2146_D1_XP_001199205.2 Vs Strpur_2146_D8_XP_001199205.2 : 49.67 % id

Strpur_2146_D1_XP_001199205.2 Vs Strpur_2146_D9_XP_001199205.2 : 47.02 % id

Strpur_2146_D1_XP_001199205.2 Vs Strpur_2146_D10_XP_001199205.2 : 44.37 % id

Strpur_2146_D1_XP_001199205.2 Vs Strpur_2146_D11_XP_001199205.2 : 48.34 % id

Strpur_2146_D1_XP_001199205.2 Vs Strpur_2146_D12_XP_001199205.2 : 45.70 % id

Strpur_2146_D1_XP_001199205.2 Vs Strpur_2146_D13_XP_001199205.2 : 44.37 % id

Strpur_2146_D1_XP_001199205.2 Vs Strpur_2146_D14_XP_001199205.2 : 37.25 % id

Strpur_2146_D1_XP_001199205.2 Vs Strpur_267_Adgb_XP_001195639.2 : 16.33 % id

Strpur_2146_D1_XP_001199205.2 Vs Xyljan_537_AST : 38.16 % id

Strpur_2146_D2_XP_001199205.2 Vs Strpur_2146_D3_XP_001199205.2 : 42.21 % id

Strpur_2146_D2_XP_001199205.2 Vs Strpur_2146_D4_XP_001199205.2 : 53.21 % id

Strpur_2146_D2_XP_001199205.2 Vs Strpur_2146_D5_XP_001199205.2 : 57.89 % id

Strpur_2146_D2_XP_001199205.2 Vs Strpur_2146_D6_XP_001199205.2 : 55.92 % id

Strpur_2146_D2_XP_001199205.2 Vs Strpur_2146_D7_XP_001199205.2 : 55.92 % id

Strpur_2146_D2_XP_001199205.2 Vs Strpur_2146_D8_XP_001199205.2 : 56.58 % id

Strpur_2146_D2_XP_001199205.2 Vs Strpur_2146_D9_XP_001199205.2 : 50.66 % id

Strpur_2146_D2_XP_001199205.2 Vs Strpur_2146_D10_XP_001199205.2 : 51.66 % id

Strpur_2146_D2_XP_001199205.2 Vs Strpur_2146_D11_XP_001199205.2 : 55.92 % id

Strpur_2146_D2_XP_001199205.2 Vs Strpur_2146_D12_XP_001199205.2 : 50.33 % id

Strpur_2146_D2_XP_001199205.2 Vs Strpur_2146_D13_XP_001199205.2 : 43.87 % id

Strpur_2146_D2_XP_001199205.2 Vs Strpur_2146_D14_XP_001199205.2 : 43.62 % id

Strpur_2146_D2_XP_001199205.2 Vs Strpur_267_Adgb_XP_001195639.2 : 18.83 % id

Strpur_2146_D2_XP_001199205.2 Vs Xyljan_537_AST : 43.14 % id

Strpur_2146_D3_XP_001199205.2 Vs Strpur_2146_D4_XP_001199205.2 : 45.45 % id

Strpur_2146_D3_XP_001199205.2 Vs Strpur_2146_D5_XP_001199205.2 : 51.66 % id

Strpur_2146_D3_XP_001199205.2 Vs Strpur_2146_D6_XP_001199205.2 : 52.32 % id

Strpur_2146_D3_XP_001199205.2 Vs Strpur_2146_D7_XP_001199205.2 : 52.32 % id

Strpur_2146_D3_XP_001199205.2 Vs Strpur_2146_D8_XP_001199205.2 : 50.99 % id

Strpur_2146_D3_XP_001199205.2 Vs Strpur_2146_D9_XP_001199205.2 : 49.01 % id

Strpur_2146_D3_XP_001199205.2 Vs Strpur_2146_D10_XP_001199205.2 : 43.33 % id

Strpur_2146_D3_XP_001199205.2 Vs Strpur_2146_D11_XP_001199205.2 : 43.71 % id

Strpur_2146_D3_XP_001199205.2 Vs Strpur_2146_D12_XP_001199205.2 : 50.00 % id

Strpur_2146_D3_XP_001199205.2 Vs Strpur_2146_D13_XP_001199205.2 : 39.61 % id

Strpur_2146_D3_XP_001199205.2 Vs Strpur_2146_D14_XP_001199205.2 : 43.92 % id

Strpur_2146_D3_XP_001199205.2 Vs Strpur_267_Adgb_XP_001195639.2 : 19.21 % id

Strpur_2146_D3_XP_001199205.2 Vs Xyljan_537_AST : 32.24 % id

Strpur_2146_D4_XP_001199205.2 Vs Strpur_2146_D5_XP_001199205.2 : 59.87 % id

Strpur_2146_D4_XP_001199205.2 Vs Strpur_2146_D6_XP_001199205.2 : 60.53 % id

Strpur_2146_D4_XP_001199205.2 Vs Strpur_2146_D7_XP_001199205.2 : 60.53 % id

Strpur_2146_D4_XP_001199205.2 Vs Strpur_2146_D8_XP_001199205.2 : 60.53 % id

Strpur_2146_D4_XP_001199205.2 Vs Strpur_2146_D9_XP_001199205.2 : 59.21 % id

Strpur_2146_D4_XP_001199205.2 Vs Strpur_2146_D10_XP_001199205.2 : 54.97 % id

Strpur_2146_D4_XP_001199205.2 Vs Strpur_2146_D11_XP_001199205.2 : 59.87 % id

Strpur_2146_D4_XP_001199205.2 Vs Strpur_2146_D12_XP_001199205.2 : 54.30 % id

Strpur_2146_D4_XP_001199205.2 Vs Strpur_2146_D13_XP_001199205.2 : 46.45 % id

Strpur_2146_D4_XP_001199205.2 Vs Strpur_2146_D14_XP_001199205.2 : 44.97 % id

Strpur_2146_D4_XP_001199205.2 Vs Strpur_267_Adgb_XP_001195639.2 : 17.76 % id

Strpur_2146_D4_XP_001199205.2 Vs Xyljan_537_AST : 38.56 % id

Strpur_2146_D5_XP_001199205.2 Vs Strpur_2146_D6_XP_001199205.2 : 89.47 % id

Strpur_2146_D5_XP_001199205.2 Vs Strpur_2146_D7_XP_001199205.2 : 89.47 % id

Strpur_2146_D5_XP_001199205.2 Vs Strpur_2146_D8_XP_001199205.2 : 88.16 % id

Strpur_2146_D5_XP_001199205.2 Vs Strpur_2146_D9_XP_001199205.2 : 63.82 % id

Strpur_2146_D5_XP_001199205.2 Vs Strpur_2146_D10_XP_001199205.2 : 58.94 % id

Strpur_2146_D5_XP_001199205.2 Vs Strpur_2146_D11_XP_001199205.2 : 63.82 % id

Strpur_2146_D5_XP_001199205.2 Vs Strpur_2146_D12_XP_001199205.2 : 58.28 % id

Strpur_2146_D5_XP_001199205.2 Vs Strpur_2146_D13_XP_001199205.2 : 45.39 % id

Strpur_2146_D5_XP_001199205.2 Vs Strpur_2146_D14_XP_001199205.2 : 48.32 % id

Strpur_2146_D5_XP_001199205.2 Vs Strpur_267_Adgb_XP_001195639.2 : 18.92 % id

Strpur Vs Xyljan_537_AST : 38.16 % id

Strpur_2146_D6_XP_001199205.2 Vs Strpur_2146_D7_XP_001199205.2 : 100.00 % id

Strpur_2146_D6_XP_001199205.2 Vs Strpur_2146_D8_XP_001199205.2 : 94.08 % id

Strpur_2146_D6_XP_001199205.2 Vs Strpur_2146_D9_XP_001199205.2 : 67.76 % id

Strpur_2146_D6_XP_001199205.2 Vs Strpur_2146_D10_XP_001199205.2 : 56.95 % id

Strpur_2146_D6_XP_001199205.2 Vs Strpur_2146_D11_XP_001199205.2 : 62.50 % id

Strpur_2146_D6_XP_001199205.2 Vs Strpur_2146_D12_XP_001199205.2 : 56.29 % id

Strpur_2146_D6_XP_001199205.2 Vs Strpur_2146_D13_XP_001199205.2 : 47.37 % id

Strpur_2146_D6_XP_001199205.2 Vs Strpur_2146_D14_XP_001199205.2 : 48.32 % id

Strpur_2146_D6_XP_001199205.2 Vs Strpur_267_Adgb_XP_001195639.2 : 19.59 % id

Strpur_2146_D6_XP_001199205.2 Vs Xyljan_537_AST : 37.50 % id

Strpur_2146_D7_XP_001199205.2 Vs Strpur_2146_D8_XP_001199205.2 : 94.08 % id

Strpur_2146_D7_XP_001199205.2 Vs Strpur_2146_D9_XP_001199205.2 : 67.76 % id

Strpur_2146_D7_XP_001199205.2 Vs Strpur_2146_D10_XP_001199205.2 : 56.95 % id

Strpur_2146_D7_XP_001199205.2 Vs Strpur_2146_D11_XP_001199205.2 : 62.50 % id

Strpur_2146_D7_XP_001199205.2 Vs Strpur_2146_D12_XP_001199205.2 : 56.29 % id

Strpur_2146_D7_XP_001199205.2 Vs Strpur_2146_D13_XP_001199205.2 : 47.37 % id

Strpur_2146_D7_XP_001199205.2 Vs Strpur_2146_D14_XP_001199205.2 : 48.32 % id

Strpur_2146_D7_XP_001199205.2 Vs Strpur_267_Adgb_XP_001195639.2 : 19.59 % id

Strpur_2146_D7_XP_001199205.2 Vs Xyljan_537_AST : 37.50 % id

Strpur_2146_D8_XP_001199205.2 Vs Strpur_2146_D9_XP_001199205.2 : 65.13 % id

Strpur_2146_D8_XP_001199205.2 Vs Strpur_2146_D10_XP_001199205.2 : 58.94 % id

Strpur_2146_D8_XP_001199205.2 Vs Strpur_2146_D11_XP_001199205.2 : 63.16 % id

Strpur_2146_D8_XP_001199205.2 Vs Strpur_2146_D12_XP_001199205.2 : 56.29 % id

Strpur_2146_D8_XP_001199205.2 Vs Strpur_2146_D13_XP_001199205.2 : 48.03 % id

Strpur_2146_D8_XP_001199205.2 Vs Strpur_2146_D14_XP_001199205.2 : 49.66 % id

Strpur_2146_D8_XP_001199205.2 Vs Strpur_267_Adgb_XP_001195639.2 : 19.59 % id

Strpur_2146_D8_XP_001199205.2 Vs Xyljan_537_AST : 38.16 % id

Strpur_2146_D9_XP_001199205.2 Vs Strpur_2146_D10_XP_001199205.2 : 52.32 % id

Strpur_2146_D9_XP_001199205.2 Vs Strpur_2146_D11_XP_001199205.2 : 57.24 % id

Strpur_2146_D9_XP_001199205.2 Vs Strpur_2146_D12_XP_001199205.2 : 53.64 % id

Strpur_2146_D9_XP_001199205.2 Vs Strpur_2146_D13_XP_001199205.2 : 45.39 % id

Strpur_2146_D9_XP_001199205.2 Vs Strpur_2146_D14_XP_001199205.2 : 46.98 % id

Strpur_2146_D9_XP_001199205.2 Vs Strpur_267_Adgb_XP_001195639.2 : 19.59 % id

Strpur_2146_D9_XP_001199205.2 Vs Xyljan_537_AST : 38.16 % id

Strpur_2146_D10_XP_001199205.2 Vs Strpur_2146_D11_XP_001199205.2 : 56.29 % id

Strpur_2146_D10_XP_001199205.2 Vs Strpur_2146_D12_XP_001199205.2 : 49.01 % id

Strpur_2146_D10_XP_001199205.2 Vs Strpur_2146_D13_XP_001199205.2 : 45.03 % id

Strpur_2146_D10_XP_001199205.2 Vs Strpur_2146_D14_XP_001199205.2 : 43.62 % id

Strpur_2146_D10_XP_001199205.2 Vs Strpur_267_Adgb_XP_001195639.2 : 15.65 % id

Strpur_2146_D10_XP_001199205.2 Vs Xyljan_537_AST : 35.76 % id

Strpur_2146_D11_XP_001199205.2 Vs Strpur_2146_D12_XP_001199205.2 : 56.29 % id

Strpur_2146_D11_XP_001199205.2 Vs Strpur_2146_D13_XP_001199205.2 : 47.37 % id

Strpur_2146_D11_XP_001199205.2 Vs Strpur_2146_D14_XP_001199205.2 : 48.32 % id

Strpur_2146_D11_XP_001199205.2 Vs Strpur_267_Adgb_XP_001195639.2 : 20.95 % id

Strpur_2146_D11_XP_001199205.2 Vs Xyljan_537_AST : 36.84 % id

Strpur_2146_D12_XP_001199205.2 Vs Strpur_2146_D13_XP_001199205.2 : 43.71 % id

Strpur_2146_D12_XP_001199205.2 Vs Strpur_2146_D14_XP_001199205.2 : 43.62 % id

Strpur_2146_D12_XP_001199205.2 Vs Strpur_267_Adgb_XP_001195639.2 : 21.09 % id

Strpur_2146_D12_XP_001199205.2 Vs Xyljan_537_AST : 37.09 % id

Strpur_2146_D13_XP_001199205.2 Vs Strpur_2146_D14_XP_001199205.2 : 38.26 % id

Strpur_2146_D13_XP_001199205.2 Vs Strpur_267_Adgb_XP_001195639.2 : 18.54 % id

Strpur_2146_D13_XP_001199205.2 Vs Xyljan_537_AST : 30.07 % id

Strpur_2146_D14_XP_001199205.2 Vs Strpur_267_Adgb_XP_001195639.2 : 15.17 % id

Strpur_2146_D14_XP_001199205.2 Vs Xyljan_537_AST : 28.67 % id

Strpur_267_Adgb_XP_001195639.2 Vs Xyljan_537_AST : 16.11 % id
