# Supplementary figures and images for: Phylogeny of Echinoderm Hemoglobins
Source: PLoS One. 2015 Aug 6;10(8):e0129668. doi: 10.1371/journal.pone.0129668 (PMC4527676; doi:10.1371/journal.pone.0129668)

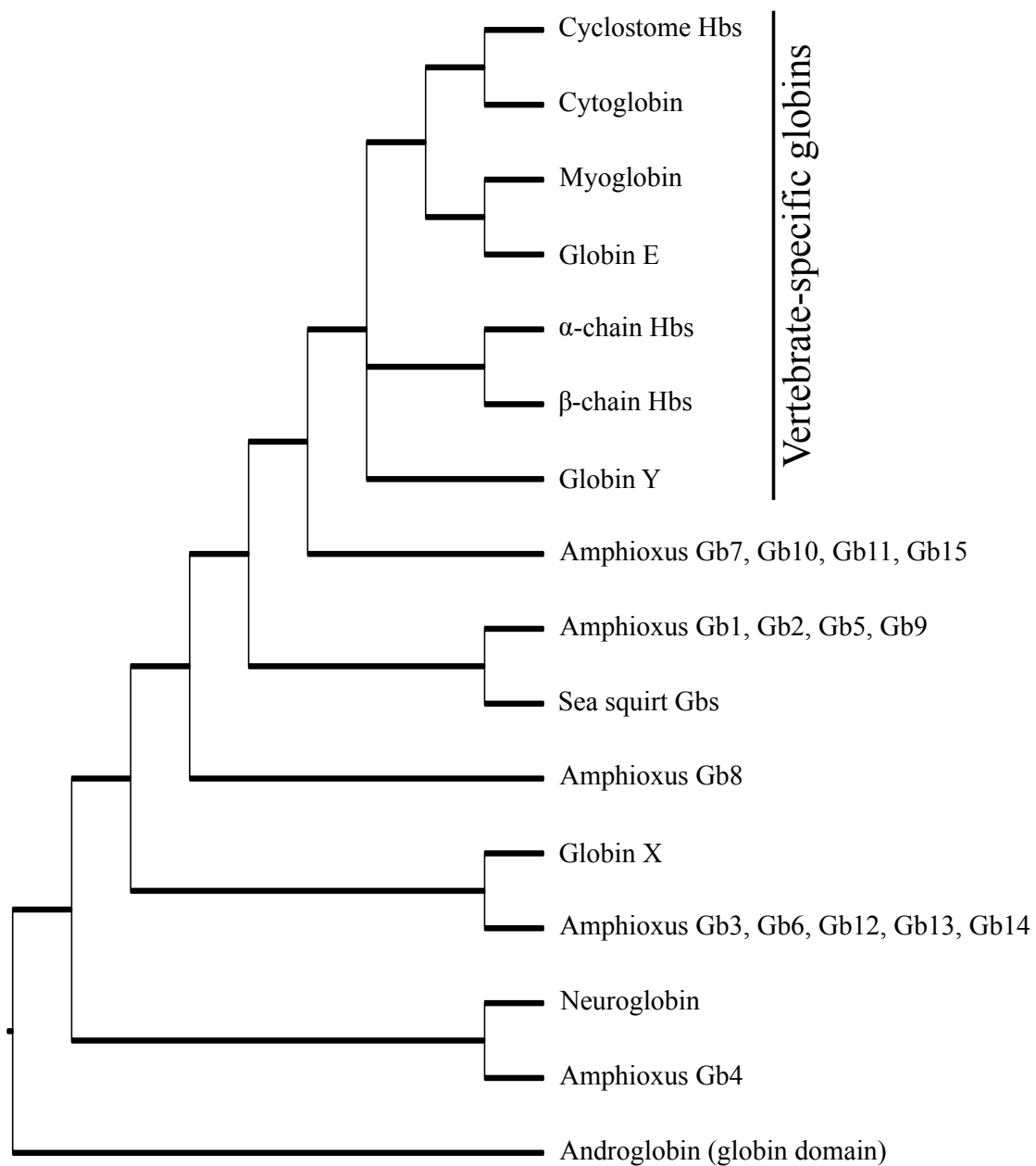

Supplement: S3 Fig — (PDF) [file pone.0129668.s003.pdf]

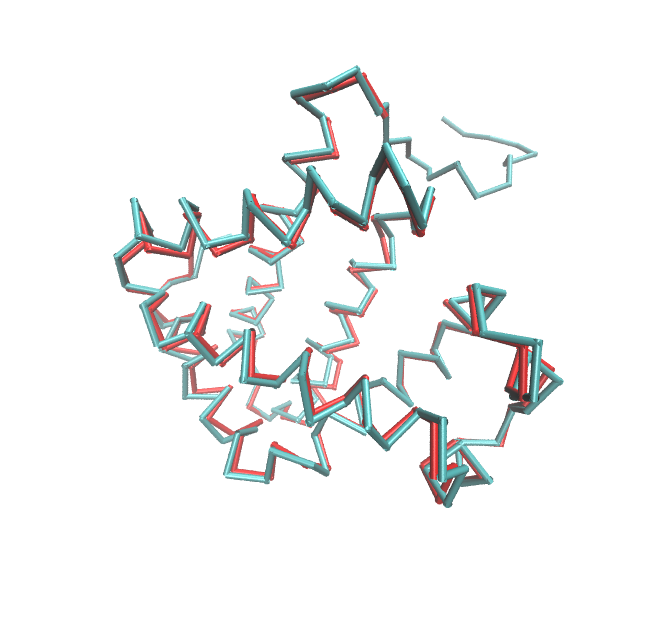

Supplement: S4 Fig — (BMP) [file pone.0129668.s004.bmp]
