# Supplementary material for: Phylogeny of Echinoderm Hemoglobins
Source: PLoS One. 2015 Aug 6;10(8):e0129668. doi: 10.1371/journal.pone.0129668 (PMC4527676; doi:10.1371/journal.pone.0129668)
Supplement: S1 Table — (DOC) [file pone.0129668.s005.doc]

Table S1. Echinoderm species with genomes/transcriptomes showing hits with homologs of the *S. purpuratus* globins.

| Species | Class | 2146 residues XP_001199205.2  416 residues  XP_003725467.1 | 166 residues  XP_003729167.1 | Androglobin isoforms 1&2  XP_003724134.1  XP_001186225.2  XP_001195639.2 |
| --- | --- | --- | --- | --- |
| *Allocentrotus fragilis* | Crinoidea | Yes, 1124 residues |  | Yes |
| *Amphiura filiformis* | Ophiuroidea | Yes | Yes |  |
| *Apostichopus japonicus* | Holothuroidea | Yes | Yes | Yes |
| *Asterias rubens* | Asteroidea | Yes | Yes | Yes |
| *Echinaster spinulosis* | Echinoidea | Yes |  | Yes |
| *Gephyroccrinus messingi* | Crinoidea |  |  | Yes |
| *Hemicentrotus pulcherrimus* | Crinoidea | Yes, 1143 residues |  |  |
| *Labidiaster annulatus* | Asteroidea | Yes | Yes | yes |
| *Olligometra serripina* | Crinoidea |  |  | Yes |
| *Ophiothrix spiculata* | Ophiuroide |  |  | Yes |
| *Peribolaster folliculatus* | Asteroidea |  | Yes |  |
| *Pisaster ochraceus* | Asteroidea | Yes | Yes | Yes |
| *Psilaster charcoti* | Asteroidea |  | Yes | Yes |
| *Pseudocentrotus depressus* | Crinoidea | Yes, 1123 residues |  |  |
| *Psathryometra fragilis* | Crinoidea |  |  | Yes |
| *Psolus spp* | Holothuroidea |  |  | Yes |
| *Pteraster tesselatus* | Asteroidea | Yes |  | Yes |
| *Remaster gourdoni* | Asteroidea |  | Yes |  |
| *Stichopus chloronatus* | Holothuroidea |  | Yes | Yes |
| *Strongylocentrotus droebachiensis* | Crinoidea | Yes |  |  |
| *Strongylocentrotus franciscanus* | Crinoidea | Yes, 1071 residues |  |  |
| *Strongylocentrotus intermedius* | Crinoidea | Yes, 1134 residues |  |  |
| *Strongylocentrotus nudus* | Crinoidea | Yes, 1134 residues |  |  |
| *Strongylocentrotus pallidus* | Crinoidea | Yes |  |  |
| *Synapta maculate* | Echinoidea |  |  | Yes |
| *Xyloplac janetae* | Asteroidea | Yes |  | Yes |
