# Supplementary material for: Phylogeny of Echinoderm Hemoglobins
Source: PLoS One. 2015 Aug 6;10(8):e0129668. doi: 10.1371/journal.pone.0129668 (PMC4527676; doi:10.1371/journal.pone.0129668)
Supplement: S2 Table — (DOCX) [file pone.0129668.s006.docx]

Supplemental Table 2. Intron locations in selected echinoderm Hbs

| Species | Hb identification | Locations in Mb fold and phase |
| --- | --- | --- |
| *Hemipholis cordifera*  *(elongate)* | Hb1, Kj1027890  Hb2, KJ027591 | B12.2, G7.0  B12.2, G7.0 |
| *Ophiactis simplex* | HbA, KJ1027592  HbB, Kj027593 | B12.2, G7.0  B12.2, G7.0 |
| *Caudina arenicola* | Hb C (PDB: 1hlb)  Hb D (PDB: 1hlm) | B12.2, G7.0  B12.2, G7.0 |
| *S. purpuratus* | 166, XP_003729167.1  2146, XP_001199205.2  416, XP_003725467.1 | B12.2, G7.0  B12.2, G7.0  B12.2, G7.0 |
