# Supplementary material for: Phylogeny of Echinoderm Hemoglobins
Source: PLoS One. 2015 Aug 6;10(8):e0129668. doi: 10.1371/journal.pone.0129668 (PMC4527676; doi:10.1371/journal.pone.0129668)
Supplement: S3 Table — (DOC) [file pone.0129668.s007.doc]

Table S3. Vertebrate, plant and outgroup sequences used in the phylogenetic analyses.

| **Species** | **Accession number** |
| --- | --- |
| *Gallus gallus* GbE | NP_001008786.1 |
| *Meleagris gallopavo* GbE | XP_003202379.1 |
| *Xenopus tropicalis* GbX | NP_001011196.1 |
| *Carassius auratus* GbX | CAG25724.1 |
| *Tetraodon nigroviridis* GbX | CAG25726.1 |
| *Anolis carolinensis* GbX | XP_003228427.1 |
| *Meleagris gallopavo* GbY | XP_003211469.1 |
| *Xenopus laevis* GbY | NP_001089155.1 |
| *Danio rerio* Mb | NP_956880.1 |
| *Bos taurus* Mb | NP_776306.1 |
| *Gallus gallus* Mb | NP_001161224.1 |
| *Bovichtus variegatus* Ngb | CAR57912.1 |
| *Carassius auratus* Ngb | CAP69822.1 |
| *Gymnodraco acuticeps* Ngb | CAR57913.1 |
| *Chelonia mydas* Cygb | EMP23772.1 |
| *Phodopus sungorus* Cygb | CAX11509.1 |
| *Columba livia* Cygb_ | EMC89810.1 |
| *Ictalurus punctatus* HbA | ADO29166.1 |
| *Homo sapiens* HbA | AAK37554.1 |
| *Gallus gallus* HbA | NP_001004376.1 |
| *Homo sapiens* HbB | CAA23758.1 |
| *Anser indicus* HbB | ACT81104.1 |
| *Ictalurus punctatus* HbB | ADO29344.1 |
| *Mordacia mordax* Cyc Hb | P21197.1 |
| *Lethenteron japonicum* Cyc Hb | BAF47286.1 |
| *Petromyzon marinus* Cyc Hb | P02208.1 |
| *Arabidopsis thaliana* Plant Hb | NP_179204.1 |
| *Arabidopsis thaliana* Plant Hb | NP_187663.1 |
| *Lupinus luteus* Plant Hb | P02239 |
| *Bacillus subtilis* NONHEME | NP_388348.1 |
| *Bacillus amyloliquefaciens* NONHEME | YP_001420131.1 |
